# Poly(U) polymerase activity in *Caenorhabditis elegans* regulates abundance and tailing of sRNA and mRNA

**DOI:** 10.1093/genetics/iyae120

**Published:** 2024-07-28

**Authors:** Leanne H Kelley, Ian V Caldas, Matthew T Sullenberger, Kevin E Yongblah, Adnan M Niazi, Anoop Iyer, Yini Li, Patrick Minty Tran, Eivind Valen, Yasir H Ahmed-Braimah, Eleanor M Maine

**Affiliations:** Department of Biology, Syracuse University, 107 College Place, Syracuse, NY 13244, USA; Department of Biology, Syracuse University, 107 College Place, Syracuse, NY 13244, USA; Department of Biology, Syracuse University, 107 College Place, Syracuse, NY 13244, USA; Department of Biology, Syracuse University, 107 College Place, Syracuse, NY 13244, USA; Computational Biology Unit, Department of Informatics, University of Bergen, 5008 Bergen, Norway; Department of Biology, Syracuse University, 107 College Place, Syracuse, NY 13244, USA; Department of Biology, Syracuse University, 107 College Place, Syracuse, NY 13244, USA; Department of Biology, Syracuse University, 107 College Place, Syracuse, NY 13244, USA; Computational Biology Unit, Department of Informatics, University of Bergen, 5008 Bergen, Norway; Department of Biology, Syracuse University, 107 College Place, Syracuse, NY 13244, USA; Department of Biology, Syracuse University, 107 College Place, Syracuse, NY 13244, USA

**Keywords:** *Caenorhabditis elegans*, uridylation, 3′ tailing, small RNA, poly(U) polymerase

## Abstract

Terminal nucleotidyltransferases add nucleotides to the 3′ end of RNA to modify their stability and function. In *Caenorhabditis elegans*, the terminal uridyltransferases/poly(U) polymerases PUP-1 (aka CID-1, CDE-1), PUP-2, and PUP-3 affect germline identity, survival, and development. Here, we identify small RNA (sRNA) and mRNA targets of these PUPs and of a fourth predicted poly(U) polymerase, F43E2.1/PUP-4. Using genetic and RNA sequencing approaches, we identify RNA targets of each PUP and the U-tail frequency and length of those targets. At the whole organism level, PUP-1 is responsible for most sRNA U-tailing, and other PUPs contribute to modifying discrete subsets of sRNAs. Moreover, the expression of PUP-2, PUP-3, and especially PUP-4 limits uridylation on some sRNAs. The relationship between uridylation status and sRNA abundance suggests that U-tailing can have a negative or positive effect on abundance depending on context. sRNAs modified by PUP activity primarily target mRNAs that are ubiquitously expressed or most highly expressed in the germline. mRNA data obtained with a Nanopore-based method reveal that the addition of U-tails to nonadenylated mRNA is substantially reduced in the absence of PUP-3. Overall, this work identifies PUP RNA targets, defines the effect of uridylation loss on RNA abundance, and reveals the complexity of PUP regulation in *C. elegans* development.

## Introduction

Numerous posttranscriptional mechanisms regulate mRNA stability and function, including covalent modifications, such as phosphorylation, methylation, ubiquitination, nucleobase modifications, mRNA 5′ capping, and 3′ tailing, as well as RNA interference (RNAi)-related mechanisms. In RNAi, small RNAs (sRNAs) associate with an Argonaute effector protein to form RNA-induced silencing complexes (RISCs) and target mRNAs by sequence complementarity to silence or, in some cases, promote their activity (Billi *et al.* 2014). The best studied classes of sRNAs in the context of RNAi-related mechanisms are short interfering (si) RNAs, micro (mi) RNAs, and PIWI (pi) RNAs. While sRNAs function in posttranscriptional regulation of mRNAs, they themselves can also be posttranscriptionally modified, e.g. by the addition of 3′ nontemplated nucleotide tails (De Almeida *et al*. 2018).

3′ tailing is a common phenomenon whose function and regulation are well understood in some contexts and poorly understood in others. One class of 3′ modification, uridylation, is accomplished by a family of terminal nucleotidyltransferases (TENTs) called poly(U) polymerases or terminal uridyltransferases (PUPs, TUTases) (reviewed by Scheer *et al.* 2016; De Almeida *et al.* 2018; Warkocki *et al.* 2018). PUPs/TUTases are conserved across eukaryotes, including algae, protozoa, yeast, plants, flies, nematodes, and vertebrates (Liudkovska and Dziembowski 2021).

Uridylation is classically described as negatively regulating mRNA stability; the U-tail in these cases is often added 3′ to a poly(A) tail and typically contains fewer than 5 nucleotides (Rissland *et al*. 2007; Rissland and Norbury 2009; Schmidt *et al.* 2010; Lim *et al.* 2014; Lackey *et al.* 2016; Morgan *et al.* 2017; Chang *et al.* 2018; Lipińska-Zubrycka *et al.* 2023). In vertebrates, fission yeast, and *Arabidopsis*, uridylation of polyadenylated mRNAs triggers the recruitment of conserved 3′–5′ exonucleases, such as Dis3L2, or 5′–3′ exonucleases, such as Lsm1-7 (Rissland and Norbury 2009; Scheer *et al.* 2021; Wu *et al.* 2023). Uridylation of histone mRNAs, which lack polyadenylation, has also been described and linked to degradation (Mullen and Marzluff 2008; Lackey *et al.* 2016). In addition, mRNA cleavage products produced by miRNA-mediated RISC activity are reportedly uridylated (Shen and Goodman 2004). Interestingly, uridylation can protect mRNA from 3′–5′ degradation in some cases and ensure that 5′–3′ degradation occurs instead (Sement *et al*. 2013; Scheer *et al.* 2021).

As is the case for mRNA, 3′ uridylation of miRNA and siRNA has also been implicated in promoting turnover (Li *et al.* 2005; Lehrbach *et al.* 2009; van Wolfswinkel *et al.* 2009; Ibrahim *et al.* 2010; Kim *et al.* 2015; see Liudkovska and Dziembowski 2021) and, in at least some cases, U-tailed miRNAs are degraded by Dis3L2 exonuclease (Chang *et al*. 2013; Faehnle *et al.* 2014; Reimão-Pinto *et al.* 2015, 2016; Yang *et al.* 2020). In *Caenorhabditis elegans*, increased abundance of (at least some) CSR-1 class siRNAs has been observed in the absence of PUP-1 (van Wolfswinkel *et al.* 2009; Xu *et al.* 2018). However, U-tailing is not a global mechanism for miRNA decay in *C. elegans* or human cells (Vieux *et al.* 2021; Yang *et al.* 2022). Notably, Yang *et al*. (2022) found that U-tailing correlates with increased abundance of some miRNAs in HEK293T cells, a result they interpret to mean the uridylation positively regulates abundance of those miRNAs. In addition, 3′ uridylation can regulate sRNA biogenesis and activity by altering miRNA target specificity (Yang *et al.* 2019, 2022), regulating which arm is selected for miRNA maturation (Kim *et al.* 2020), limiting production of siRNAs from deadenylated mRNAs (Scheer *et al.* 2021), and preferentially routing siRNAs to specific Argonaute protein(s) (de Albuquerque *et al.* 2015; Xu *et al.* 2018).

PUPs/TUTases are critical for development, particularly in the vertebrate and invertebrate germline during gamete formation and in the early embryo (Thornton *et al.* 2014; Morgan *et al.* 2017, 2019; Spracklin *et al.* 2017; Chang *et al.* 2018; Li and Maine 2018; Li *et al.* 2021). Uridylation has been shown to promote widespread turnover of mRNAs during specific developmental windows, e.g. during oocyte maturation (Morgan *et al.* 2017; Wu *et al.* 2023), during spermatogenesis (Morgan *et al.* 2019), and in the early embryo to remove maternal mRNA and facilitate the transition to embryonic gene expression (Chang *et al.* 2018; Zhao *et al.* 2022). In plants, uridylation promotes development by limiting inappropriate sRNA production to allow correct expression of an essential photosynthesis regulator (Wang *et al.* 2022).

*Caenorhabditis elegans* PUP-encoding genes, *pup-1* (aka *cid-1*, *cde-1*), *pup-2*, and *pup-3*, promote germline and early embryonic development (Li and Maine 2018; Li *et al.* 2021). Loss of *pup-1* function reduces U-tailing of miRNAs and siRNAs (van Wolfswinkel *et al.* 2009; Vieux *et al.* 2021) as well as viral transcripts produced upon Orsay virus infection (Le Pen *et al.* 2018). We hypothesize that *pup* developmental defects, which are exacerbated at high culture temperatures, arise due to inappropriate gene expression resulting from reduced U-tailing of sRNA and mRNA. Here, we used Illumina sRNA-seq and Oxford Nanopore Technologies (ONT) direct cDNA sequencing (Nano3P-seq; Begik *et al.* 2023) to identify sRNAs and mRNAs that are U-tailed by 1 or more PUP. We included PUP-1, PUP-2, PUP-3, and a fourth gene, F43E2.1, with PUP activity in a heterologous system (Preston *et al.* 2019) in our analyses. We show that F43E2.1 protein is expressed in both germline and soma, and expression promotes aspects of germline development. Given its role in U-tailing certain RNAs, as well as the Preston *et al.* (2019) findings, we refer to *F43E2.1* as *pup-4*. PUP-1 is primarily responsible for uridylating siRNAs, miRNAs, and piRNAs, whereas PUP-2, PUP-3, and PUP-4 uridylate distinct subsets of those sRNAs. Moreover, uridylation frequency of certain sRNAs increases in the absence of PUP-2, PUP-3, and especially PUP-4, suggesting these enzymes may interfere with PUP-1 activity. For mRNA, we observe reduced U-tailing of nonadenylated mRNAs in the absence of PUP-3. Our data suggest that uridylation can positively and negatively regulate sRNA and mRNA abundance, depending on context. Overall, this work establishes the global molecular consequences of uridylation loss and sets the stage for future work on uridylation's specific functions in *C. elegans* development.

## Materials and methods

### *Caenorhabditis elegans* strains and culture

Nematodes were cultured using standard methods (Epstein and Shakes 1995). We used the following mutations: linkage group (LG) I: *pup-3(tm5089), omIs10[3xflag::pup-3]*; LGII: *pup-4(om140)*, *pup-4(om141)*, *omIs12[pup-4::3xflag]*; LGIII: *pup-1(tm1021)*, *pup-2(tm4344)*, *pup-1/-2(om129)*, *omIs7[pup-2::3xflag]*, *omIs8[pup-1::3xmyc]* (Li and Maine 2018), *glp-1(q231ts)*. *glp-1(q231)* was maintained over the balancer chromosome *hT2 [bli-4(e937) let-?(q272) qIs48]*. *pup-1/-2(om129)*, *pup-1(tm1021)*, and *pup-2(tm4344)* were maintained over the balancer chromosome *qC1[dpy-19(e1259ts) glp-1(q339) nIs189[myo-2::gfp]]* (abbreviated *qC1gfp* below). We used co-CRISPR-Cas9 gene editing to isolate *pup-4(om140)* and *pup-4(om141)* deletion alleles and generate *omIs12[pup-4::3xflag]* (Arribere *et al.* 2014; Paix *et al.* 2014) (see Supplementary Fig. 1). *dpy-10* and *pup-4* genome edits were simultaneously induced; visible *dpy-10* mutants were recovered and screened for a *pup-4* edit via DNA amplification; candidate edits were confirmed by DNA sequencing. The *dpy-10* mutation was either repaired to wild type using CRISPR or removed by recombination; strains were backcrossed to wild type at least 4 times prior to use. Strains used in this study are listed in Supplementary Table 1.

### sRNA sequencing and bioinformatic analysis

Strains were grown in parallel at 22°C, and F2 generation individuals were harvested. For strains carrying the *qC1gfp* balancer, we passaged F1 *pup* M+Z- individuals and harvested their F2 *pup* M-Z- offspring as 1-day old adults. “-” indicates the absence of the functional gene product; M and Z indicate maternally contributed and zygotically generated gene products, respectively. RNA was isolated from whole animals using the TRIzol (Invitrogen) method. sRNA libraries were constructed and sequenced at the Biotechnology Resource Center (BRC) Genomics Facility (RRID: SCR_021727) at the Cornell University Institute of Biotechnology. Prior to library construction, low molecular RNA was column purified and treated with RNA 5′ polyphosphatase (Epicentre) to convert triphosphate to monophosphate. Libraries were generated using the NEBNext Small Library Prep Kit; 75 bp, single-ended reads were sequenced to a minimum depth of 10M on an Illumina MiSeq. We generated 6 independent replicates for wild-type samples and 3 independent replicates for mutant samples.

For bioinformatic analysis, 3′ adapters were trimmed, and 15–26-nt-long reads with a 3′ quality cutoff of 20 were retained (Andrews 2010; Martin 2011) and mapped to the *C. elegans* WS284 reference genome. sRNAs and their 3′ nontemplated tails were identified with our smalldisco pipeline (Caldas *et al.* 2023; github.com/ianvcaldas/smalldisco). In brief, using the *C. elegans* WS272 canonical gene set GTF, miRNAs and piRNAs were selected from the ninth column (gene_biotype) and converted into BED files using BEDOPS’ *gtf2bed* (RRID:SCR_012865) (Neph *et al.* 2012). Using the tail command, 3′ tails on miRNAs and piRNAs were identified by implementing both the sense and antisense read parameters (Chou *et al.* 2015). We defined siRNAs by the gene to which they map antisense; the sirna mode was used to identify reads that mapped antisense to CDS regions. All siRNA reads mapping to exons of a single gene were grouped together and referred to as a single siRNA species, labeled by the gene ID. This list of siRNAs was used as input to identify 3′ tails using the antisense-only parameter (see Supplementary File 1).

U-tail frequency is defined as the number of U-tailed reads summed across replicates divided by the number of total reads summed across replicates, resulting in a proportion of uridylation activity for each sRNA (Supplementary Fig. 2). sRNAs with tails of any length containing a single nucleotide type were collapsed into one tail type group, i.e. A-tails, C-tails, G-tails, or U-tails of varying lengths. Tail types containing a mix of different nucleotides (e.g. UG) were categorized as “other.” The log_2_FC of uridylation frequency for mutant vs wild-type comparisons was calculated by taking the log_2_ of (uridylation frequency in mutant/uridylation frequency in wild type). There was little variation in proportion across replicates of any given genotype. Of note, the values used to calculate tailing were raw reads and not CPM because uridylation frequency is quantified as a proportion of the total reads.

To investigate U-tail proportions rather than read counts, conventional differential expression (abundance) workflows were not appropriate. Instead, as additional criteria to select the most meaningful data points, we implemented 2 strategies: (1) an abundance cutoff filter to select sRNAs with a robust number of counts and (2) a stringent overlap filter based on uridylation frequencies in mutants vs wild type. For the abundance cutoff, we selected sRNAs with a minimum count of 50 CPM in a worthwhile number of samples using edgeR::filterByExpr() (RRID:SCR_012802) (Robinson *et al.* 2010); this retained 6,439 sRNAs with sufficient counts for uridylation analysis. The overlap filter determines if the range of values among 3 wild-type replicates overlaps with the range of values among 3 mutant replicates run in parallel (Supplementary Fig. 2). Here, we calculated the frequency of U-tails for each sRNA in each replicate. If the range of replicate values for an sRNA in wild type did not overlap with those in a mutant, then we considered that uridylation was meaningfully altered and included that sRNA in downstream analyses. The overlap filter was imposed with a custom script (conceptually demonstrated in Supplementary Fig. 2). Four thousand seven hundred and seventy-six sRNAs passed the overlap filter and were retained for further analysis; we refer to these sRNAs as high-confidence uridylation targets.

For analyses pertaining to sRNA abundance (expression), the overlap filter was not imposed and sRNAs with a minimum count of 10 CPM were included. Libraries were normalized using EDASeq (RRID:SCR_006751) (Risso *et al.* 2011) and RUVSeq (RRID:SCR_006263) (with *k* = 6) (Risso *et al.* 2014), and differential abundance (DA) was assessed using edgeR (RRID:SCR_012802) (Robinson *et al.* 2010).

### Nano3P-seq, reagents, and data analysis

Animal staging was the same as for sRNA-seq experiments. mRNA 3′ tails were sequenced using Nano3P-seq, a Nanopore-based method that is a modification of direct cDNA-seq (Begik *et al.* 2023). To calibrate *tailfindr* to distinguish poly(U) and poly(A)+poly(U) from poly(A) sequences, we generated tail standards containing 3′ poly(U), poly(A), and poly(A)+poly(U) sequences of different lengths (Krause *et al.* 2019) (see Supplementary File 2 for tail standard synthesis protocol and Supplementary File 3 for tail standard sequences).

Total RNA was isolated using the TRIzol method; rRNA was depleted using RNase H as described (Duan *et al.* 2020), and samples were subjected to Nano3P-seq. Modifications to the Nano3P-seq protocol are detailed in Supplementary File 2. Briefly, an RNA–DNA hybrid oligo that can accommodate any 3′ nucleotide was used to prime reverse transcription with TGIRT-III (InGex or Lambowitz Lab, UT-Austin). The RNA strand was removed with RNase (Invitrogen, AM2286), and the remaining single-stranded cDNA was prepped for sequencing using the Nanopore Direct cDNA (SQK-DCS109) Kit. Replicates were barcoded (EXP-NBD104) and pooled. Seven runs were performed, each containing 4 (or, in one case, 2) pooled replicates, on a MinION using R9.4.1 flow cells (Supplementary File 3). FAST5 files were basecalled using *guppy_basecaller*, and sequence quality was assessed with NanoPlot (RRID:SCR_024128) (De Coster *et al.* 2018). To annotate reads, FASTQ files were demultiplexed with *guppy_barcoder* and mapped to the *C. elegans* transcriptome (RefSeq GCF_000002985.6) using Minimap2 (RRID:SCR_018550) (Li 2018). The *tailfindr* pipeline is described on GitHub under the polyu branch (https://github.com/adnaniazi/tailfindr/tree/polyu). The resulting count tables were used for analysis. The Nano3P-seq run summary is included in Supplementary File 3.

We selected transcripts with a minimum count of 10 CPM in a worthwhile number of samples using edgeR::filterByExpr() as having adequate representation in our data set; this process identified 1,337 mRNAs that we carried forward in downstream tailing analyses (RRID:SCR_012802) (Robinson *et al*. 2010). U-tail and A-tail frequencies were calculated with *tailfindr*. The same group of mRNAs was used in DA analyses. Raw counts of those mRNAs were normalized using RUVSeq with *k* = 6 (RRID:SCR_006263) (Risso *et al.* 2014) and analyzed with edgeR to identify differentially abundant mRNAs.

### Protein immunoblotting reagents and protocol

*pup-1/-2(om129)* and *glp-1(q231ts)* mutations were maintained over a balancer chromosome. Protein extracts were generated from synchronized populations of F2 generation (M-Z-) 1-day old (1d) mutants. For experiments eliminating the germline, all *glp-1(q231ts)* and control strains were grown in parallel. F2 L1 larvae of genotypes *pup-4::3xflag*, *pup-4::3xflag; pup-1/-2(om129)*, *pup-4::3xflag; glp-1(q231ts)*, and *pup-4::3xflag; glp-1(q231ts)pup-1/-2(om129)* were shifted from 15°C [permissive temperature for *glp-1(q231ts)*] to 25°C [restrictive temperature for *glp-1(q231ts)*] and harvested as 1d adults. *glp-1(q231ts)* adults were visually inspected to confirm they lacked germ cells prior to preparing protein extracts. For 22°C cultures, *pup-4::3xflag* and *pup-4::3xflag; pup-1/-2(om129)/qC1gfp* balanced heterozygotes were moved from 20 to 22°C; F1 (M+Z-) *pup-4::3xflag; pup-1/-2(om129)* hermaphrodites were picked to fresh plates, and a synchronized population of F2 (M-Z-) embryos was collected. F2 animals were harvested as 1d adults. To compare abundance of PUP-1, PUP-2, and PUP-3 in *pup-4(+)* vs *pup-4(om141)* backgrounds, strains were grown at 22°C for 2 generations, and synchronized populations of F2 (M-Z-) 1d adults were harvested. Gonads were dissected from adult hermaphrodites, and extracts were prepared as described (Guo *et al.* 2015).

Protein immunoblotting was performed as described (Li and Maine 2018). Proteins were resolved on a 10% polyacrylamide gel for PUP-4::3xFLAG analysis and on a 4–15% polyacrylamide gradient gel (Bio-Rad, #4561086) for PUP-1::3xMYC, 3xFLAG::PUP-2, and 3xFLAG::PUP-3 analyses. Antibodies and dilutions used were anti-FLAG (Sigma-Aldrich Cat# F1804, RRID:AB_262044, 1:1,000), anti-MYC (Thermo Fisher Scientific Cat# PA1-981, RRID:AB_325961, 1:1,000), anti-actin (DSHB Cat# jla20, RRID:AB_528068, 1:500 or 1:1,000), and anti-beta-tubulin (DSHB Cat# E7, RRID:AB_528499, 1:1,000). Immunolabeling was visualized using Pierce SuperSignal West Pico (PUP-1, PUP-2, and PUP-4) or Femto (PUP-3). Fiji (RRID:SCR_002285) software was used to quantify signal intensity (Schindelin *et al.* 2012). Background signal was subtracted from each PUP signal, which was then normalized to the loading control signal in the same lane. Values in each lane were then normalized to the average PUP value in the appropriate control strain carrying the epitope tag and wild-type alleles of *pup* and *glp-1* genes.

### DAPI staining and germline analysis

Intact animals were fixed with −20°C methanol, stained with DAPI, processed, and observed with a Zeiss Axioscope or Leica DM5500 as described (Li and Maine 2018). Nuclear morphology was used to identify mitotic and meiotic nuclei, including developing sperm and oocytes.

### Alignment and phylogenetic analysis

The top F43E3.1-related proteins were aligned, and neighbor-joining trees were built using Geneious software (www.geneious.com) (Kearse *et al*. 2012). Conserved domains were identified with programs available at NCBI. Intrinsically disordered regions were predicted using AlphaFold (https://alphafold.ebi.ac.uk/), DisoRDPbind (http://biomine.cs.vcu.edu/servers/DisoRDPbind/), and IUPred2a (https://iupred2a.elte.hu/).

### Statistical analysis summary

**Fig. 1.**
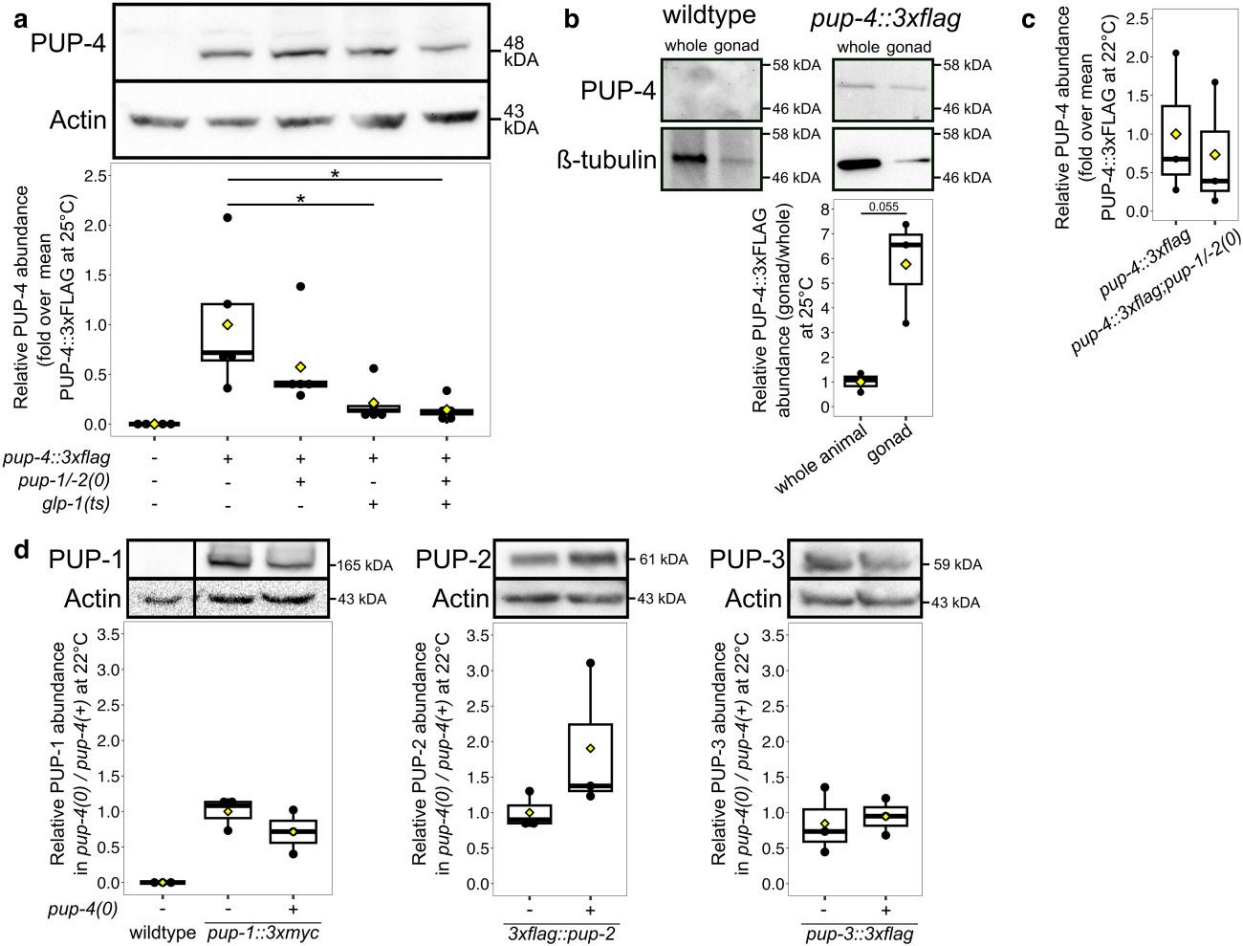
PUP-4 is expressed in germline and somatic tissues. a) Representative protein blot containing whole-protein extracts from adult hermaphrodites raised at 25°C (see *Materials and methods*). Blot was probed with anti-FLAG to visualize PUP-4::3xFLAG and anti-actin as a loading control. “+” indicates the listed allele is present in the strain. Quantification is shown for *n* = 5 biological replicates. Statistical significance was evaluated with a Dunnett's test followed by Tukey's multiple correction. *P < 0.05. PUP-4::3xFLAG signal in each strain was normalized to the average *pup-4::3xflag; pup-1/-2(+) glp-1(+)* control value. b) Representative protein blot containing extracts prepared from 25°C F2 adult hermaphrodites and dissected adult hermaphrodite gonads. Protein extract was prepared from 52 wild-type animals, 102 wild-type gonad arms, 92 *pup-4::3xflag* animals, and 193 *pup-4::3xflag* gonad arms. Blot was probed with anti-FLAG and reprobed with anti-beta-tubulin. Quantification is shown for *n* = 3 biological replicates; PUP-4::3xFLAG signal in gonads was normalized to the signal in *pup-4::3xflag* whole animals. Note that gonad tissue is primarily germline. c) Representative protein blot of extracts from adult hermaphrodites raised at 22°C. Quantification is shown for *n* = 3 biological replicates. d) Representative protein blots containing whole-protein extracts prepared from adult hermaphrodites of the genotype indicated raised at 22°C. “+” indicates the listed allele is present in the strain. *n* = 3 biological replicates of each blot. Wild-type and PUP-1 lanes are from the same blot. Statistical significance in b–d) was evaluated using a 2-sided Student's *t*-test. For all boxplots, box indicates the middle 50% of values, bar indicates the median, and diamond indicates the average.

**Fig. 2.**
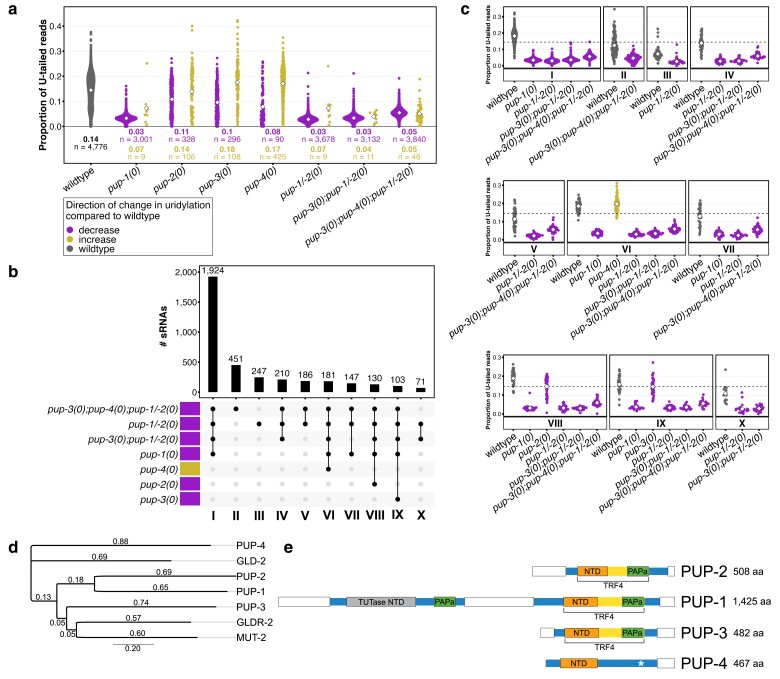
Altered sRNA U-tailing in *pup* mutants. a) Proportion of U-tailed reads for high-confidence uridylation targets in wild type and each *pup* mutant. sRNAs that pass both the abundance cutoff and overlap filter criteria (4,776) are included (see Supplementary Fig. 2). Each point represents an sRNA. Wild-type uridylation frequencies for all sRNAs are indicated; only high-confidence uridylation targets in each mutant strain (*n*) are shown. Diamond, average U-tail frequency for each group of sRNAs; value is listed below. b) UpSet plot (Lex *et al.* 2014) indicates shared and unique sRNA uridylation targets identified in different mutant strains; the top 10 intersections (out of 171) are shown. Purple, down-uridylated in mutant; gold, up-uridylated in mutant. c) Proportion of U-tailed reads is plotted for sRNAs indicated in individual columns in b) as indicated by Roman numerals. *X*-axis, genotype. *Y*-axis, proportion of U-tailed reads. Diamond, average U-tail frequency for each group of sRNAs; value is listed below. Dotted line, average uridylation frequency of the 4,776 sRNAs in wild type (∼14%). The siRNAs shown here correspond to protein-coding genes; U-tail frequencies of siRNAs corresponding to transposons are shown in Supplementary Fig. 5. d) Protein sequence alignment and neighbor-joining analysis of F43E2.1/PUP-4 and closely related *C. elegans* proteins. Value on each branch indicates the average number of substitutions per site. e) Schematic of PUP proteins showing relative positions of conserved domains. NTD, nucleotidyltransferase domain of poly(A) polymerase and TUTase; contains catalytic residues for transferring a nucleotide to RNA 3′ end. “TUTase NTD” in PUP-1 lacks catalytic residues. PAPa, poly(A) polymerase-associated domain containing a NRM. TRF4, yeast DNA polymerase sigma homology region including an NTD and NRM. *Degenerate NRM. Open boxed regions are predicted to be intrinsically disordered.

Statistical analyses and data visualization were performed using R Project for Statistical Computing (RRID:SCR_001905) and Jupyter Notebook (RRID:SCR_018315). To assess significant differences for protein quantifications, we used a Dunnett's test followed by a Tukey multiple comparisons test (Fig. 1a) or a 2-sided Student's *t*-test (Fig. 1b–d). At least 3 biological replicates were obtained. Differences in uridylation frequencies were determined by Kruskal–Wallis test followed by a Dunn's test with Bonferroni correction (Fig. 2). At least 3 biological replicates were obtained for each genotype in both sRNA-seq and Nano3P-seq experiments. For DA analysis, edgeR was used to identify significant differences with an arbitrary cutoff of fold change greater than 2 and false discovery rate (FDR) < 0.05 for sRNA data sets and a fold change of greater than 2 and FDR < 0.01 for mRNA data sets. Tissue enrichment was calculated using a tissue specificity index based on cummeRbund's csSpecificity function; *χ*^2^ tests and corrected *P*-values are reported. Gene Ontology (GO) analyses were performed using the WormBase Enrichment Tool with a *q*-value threshold of 0.1 and using all nonzero count sRNAs in our data set as the background genes (Angeles-Albores *et al.* 2016, 2018).

## Results

### PUP-4 is expressed in germline and somatic tissues

PUP-1, PUP-2, and PUP-3 are detected in the germline at a relatively high level and more lowly detected in somatic tissues (Li and Maine 2018). *F43E2.1* and *pup-1* mRNAs are present at comparable levels in our wild-type gonad transcriptome data set [reads per kilobase of transcript per million mapped reads (RPKM) of 45 and 50, respectively] (Guo *et al.* 2015); hence, we expected the F43E2.1 protein (hereafter called PUP-4) to be present in the germline. We easily detected 3xFLAG::PUP-4 and PUP-4::3xFLAG in protein extracts from dissected gonads (below) and not by immunolabeling; therefore, we relied on protein blots to evaluate PUP-4 abundance. To discern germline vs somatic expression, we first evaluated PUP-4::3xFLAG in animals with and without germ cells. We eliminated germ cells using a conditional allele of the Notch-type receptor gene, *glp-1(q231ts)*, whose product is essential for maintaining germline stem cells (Austin and Kimble 1989; Kodoyianni *et al.* 1992; Maine and Kimble 1993) (see *Materials and methods*). The average PUP-4::3xFLAG abundance in *glp-1(q231ts)* adult hermaphrodites was reduced to ∼21% of *glp-1(+)* control levels (Fig. 1a), consistent with a substantial portion of the PUP-4::3xFLAG protein pool being expressed either in the germline or in the soma upon communication from the germline. To distinguish between these alternatives, we compared PUP-4::3xFLAG expression in intact animals and dissected gonads (comprising primarily germ cells). PUP-4::3xFLAG signal was substantially enriched in dissected gonad samples (Fig. 1b). We conclude that PUP-4 is highly expressed in germ cells compared to somatic tissues.

We previously observed a ∼2.5-fold increase in PUP-3 abundance in *pup-1/-2(om129)* double mutants compared to wild type, suggesting that PUP-1 and PUP-2 together directly or indirectly limit PUP-3 expression (Li and Maine 2018). (Unless otherwise indicated, we hereafter use “0” to indicate the null mutations described in *Materials and methods*.) To determine whether PUP-1 and PUP-2 influence PUP-4 abundance, we compared PUP-4::3xFLAG level in *pup-1/-2(0)* double mutant and *pup-1/-2(+)* control adults (see *Materials and methods* for culture scheme). Although the average PUP-4::3xFLAG signal was reduced in *pup-4::3xflag; pup-1/-2(0)* F2 adults at 25 and 22°C compared to *pup-4::3xflag; pup-1/-2(+)* controls, the difference was not statistically significant at either temperature (Fig. 1a and c). We compared somatic PUP-4::3xFLAG expression in *pup-4::3xflag; pup-1/-2(0)glp-1(q231ts)* and *pup-4::3xflag; pup-1/-2(+)glp-1(q231ts)* adults raised in parallel at 25°C. PUP-4::3xFLAG abundance was comparable in both strains, suggesting the loss of PUP-1/-2 had little effect on PUP-4 level in somatic tissues. Low PUP-4::3xFLAG abundance in *pup-1/-2(0)glp-1(q231ts)* adults (∼14% of control levels) (Fig. 1a) is consistent with the germline being required for most of the PUP-4 pool.

We evaluated the impact of PUP-4 loss on expression of other PUP proteins in *pup-4(0)* strains that carried endogenously tagged *pup-1::3xmyc*, *3xflag::pup-2*, or *3xflag::pup-3* (Li and Maine 2018) (see *Materials and methods*). In synchronized F2 M-Z- adult hermaphrodites, the abundance of PUP-1::3xMYC, 3xFLAG::PUP-2, and 3xFLAG::PUP-3 was statistically similar in *pup-4(0)* mutants vs *pup-4(+)* controls (Fig. 1d), and we conclude that PUP-4 does not substantially influence production of other PUPs.

### *pup-4* mutants have mild germline defects

We analyzed the *pup-4* deletion phenotype using 2 CRISPR-Cas9-generated deletion alleles, *pup-4(om140)* and *pup-4(om141)* (see *Materials and methods*; Supplementary Fig. 1). Null alleles of *pup-1*, *pup-2*, and *pup-3* are temperature sensitive (Li and Maine 2018); therefore, we evaluated *pup-4* mutants at elevated temperature. More than 99% of *pup-4(om140)* and *pup-4(om141)* mutants raised at 25°C were fertile over >10 generations (Supplementary Table 2a). We examined DAPI-stained F2, F3, and F4 adult animals and observed some fertile F3 and F4 animals with impaired germline development in 1 of the 2 gonad arms, e.g. 13% of *pup-4(om141)* gonad arms in the F3 generation failed to make embryos despite the presence of sperm and oocytes in the arm or, more rarely, failed to produce oocytes (Supplementary Table 2a). This *pup-4* knockout germline phenotype is similar to that previously described for *pup-3(tm5089)* (Li and Maine 2018).

We tested for genetic interactions between *pup-4(om141)* [hereafter called *pup-4(0)*] and other *pup* genes by asking (1) if *pup-3(0); pup-4(0)* double mutants have a more severe phenotype than either single mutant and (2) whether *pup-4(0)* alters the *pup-1/-2(0)* double and/or *pup-3(0); pup-1/-2(0)* triple mutant phenotype with respect to germline development, fertility, and embryonic viability (see *Materials and methods*). Nearly all *pup-3(0); pup-4(0)* animals produced gametes in at least 1 gonad arm and were fertile at 25°C (Supplementary Table 2). Germline defects in the F3 and F4 generations resembled those in *pup-4(0)*; in addition, the very rare gonad arm failed to make either sperm or oocytes, and fertile animals often contained endomitotic oocytes in the uterus, as previously described for *pup-3(0)* single mutants (Supplementary Table 2a). Germline defects were slightly more penetrant in the *pup-3(0); pup-4(0)* double mutant than in the *pup-4(0)* single mutant (Supplementary Table 2b) and similar overall to the *pup-3(0)* single mutant (Li and Maine 2018). Based on these data, PUP-3 and PUP-4 do not appear to be functionally redundant.

We observed little effect of *pup-4(0)* on the *pup-1/-2(0)* and *pup-3(0); pup-1/-2(0)* phenotypes at 25°C (Supplementary Table 2b). In the F3 and F4 generations, *pup-4(0); pup-1/-2(0)* sterile adults typically lacked germ cells, similar to the *pup-1/-2(0)* double mutant, whereas *pup-3(0); pup-4(0); pup-1/-2(0)* sterile adults often contained germ cells (Supplementary Table 2b), similar to *pup-3(0); pup-1/-2(0)* (Li and Maine 2018). These results suggest that loss of PUP-4 does not substantially modify the *pup-1/-2(0)* or *pup-3(0); pup-1/-2(0)* phenotypes under our laboratory conditions.

### sRNA 3′ tailing is altered in *pup* mutants

To identify sRNA targets of the PUP proteins, we obtained sRNA-seq data for synchronized wild-type and *pup-1(0)*, *pup-2(0)*, *pup-3(0)*, *pup-4(0)*, *pup-1/-2(0), pup-3(0); pup-1/-2(0)*, and *pup-3(0); pup-4(0); pup-1/-2(0)* mutant strains raised in parallel. We identified siRNAs, miRNAs, and piRNAs and their 3′ tail sequences for each genotype (see *Materials and methods*). For siRNA analysis, we combined all siRNAs targeting the same gene and, although most siRNAs in our data sets are 22G RNAs as is common in *C. elegans*, we included other low-abundance siRNAs (Supplementary Fig. 3). In wild type, U-tail frequencies ranged widely within all 3 sRNA classes, averaging ∼14% for siRNAs, ∼6% for miRNAs, and ∼4% for piRNAs (Supplementary Fig. 4a). Single nucleotide addition, (U)_1_, comprised ∼64% of siRNA U-tails, 94% of miRNA U-tails, and ∼96% of piRNA U-tails; longer tails were common only for siRNAs, e.g. ∼20% (U)_2_ and ∼10% (U)_3_ (Supplementary Fig. 4b). Uridylation was severely reduced on all sRNA classes in the absence of PUP-1 (Supplementary Fig. 4). Of the siRNA tails present in *pup-1(0)* mutants, >91% were (U)_1_, suggesting PUP-1 promotes oligouridylation (Supplementary Fig. 4b). Subtle, yet significant, shifts in miRNA and piRNA U-tail lengths were also observed in *pup-1(0)* mutants (Supplementary Fig. 4). In contrast to *pup-1*, uridylation was subtly altered on all 3 sRNA classes in *pup-2(0)*, *pup-3(0)*, and *pup-4(0)* mutants (Supplementary Fig. 4). Although there was a net *increase* in U-tailing in *pup-4(0)* mutants compared to wild type (Supplementary Fig. 4), PUP-4 does not appear to add a different tail type (Supplementary Fig. 6). Strikingly, uridylation was not eliminated in any *pup* strain, consistent with a low level of U-tailing by one or more additional TENTs in the absence of the defined PUPs (Supplementary Fig. 4).

Before further analyzing PUP targets, we implemented a filtering strategy to identify with high confidence the sRNAs modified by PUP activity. For each *pup* mutant strain, we identified sRNAs for which the range of U-tail frequencies among replicates did not overlap the range of U-tail frequencies among wild-type replicates (Supplementary Fig. 2; see *Materials and methods*). The 4,776 sRNAs that pass this conservative test in at least 1 mutant strain are considered to be high-confidence uridylation targets of one or more PUP enzymes (Fig. 2a). Most high-confidence targets are siRNAs (Supplementary Table 3). More than 3,000 high-confidence uridylated sRNAs lost U-tails in *pup-1(0)* single mutants; their average U-tail frequency was reduced to ∼3% (Fig. 2a). Relatively few sRNAs lost U-tails in *pup-2(0)*, *pup-3(0)*, or *pup-4(0)* single mutants where their average frequency was more modestly reduced to ∼8–11% (Fig. 2a). Notably, U-tailing *increased* for other sets of sRNAs in these 3 single mutants, especially in *pup-4(0)* where average U-tail frequency for a set of 425 sRNAs increased to 17% (Fig. 2a). Increased U-tailing may reflect a loss of inhibition by the slower acting or, in the case of PUP-4, perhaps inactive enzyme. In the *pup-3(0); pup-4(0); pup-1/-2(0)* quadruple mutant, where U-tailing frequency is higher than in the *pup-3(0); pup-1/-2(0)* triple mutant (Fig. 2a; Supplementary Fig. 4a), tailing is presumably accomplished by another TENT that can better access sRNAs in the absence of PUP-4.

The phylogenetic relationships among *C. elegans* proteins that most closely share domain homology with PUP-4/F43E2.1 are shown in Fig. 2d. These include PUP-1, PUP-2, PUP-3, MUT-2/RDE-3 (documented to add UG to RNA fragments; Preston *et al.* 2019; Shukla *et al.* 2020), GLD-2 (documented to add A to mRNA; Nousch *et al.* 2017), and GLDR-2 (documented to add A to miRNA; Vieux *et al*. 2021). PUP-4 is the most distantly related protein in the group and likely to be the oldest (Fig. 2d). It may have relatively low uridylation activity because it lacks the PAPa and nucleotide recognition motif (NRM) regions present in PUP-1, PUP-2, and PUP-3 (Fig. 2e). The N-terminal region of PUP-1, known to interact with EGO-1 RNA-directed RNA polymerase (van Wolfswinkel *et al*. 2009), may facilitate recruitment to sRNA targets and is absent from other PUPs.

### Shared vs unique uridylation targets

To better understand the relationship among PUP activities, we compared the sRNA uridylation targets in strains carrying single and multiple *pup* gene mutations. A larger number of sRNAs lose U-tails in animals carrying *pup-1(0)* and a second *pup* mutation than in *pup-1(0)* single mutants (Fig. 2a; Supplementary Fig. 9). For example, >3,800 sRNAs have reduced U-tail frequency in *pup-3(0); pup-4(0); pup-1/-2(0)* quadruple mutants compared to ∼3,000 in *pup-1(0)* (Fig. 2a). The largest group of sRNAs (1,924) lose uridylation in the 4 strains carrying *pup-1(0)*, while other PUP-1 targets are also uridylated by PUP-2 (130; Fig. 2b and c, VIII) or PUP-3 (103; Fig. 2b and c, IX). Moreover, 181 PUP-1 targets have increased U-tailing in *pup-4(0)* mutants (Fig. 2, b and c, VI). We note that relatively few sRNAs were solely targeted by PUP-2, PUP-3, or PUP-4. For example, U-tailing was reduced for only 51 sRNAs—not targeted by PUP-1—in *pup-4(0)* mutants (Supplementary Fig. 9). Together, these results reinforce the conclusion that PUP-1 is primarily responsible for sRNA uridylation in the adult hermaphrodite and point to unique roles for PUP-2, PUP-3, and PUP-4.

We considered whether differences in PUP-2, PUP-3, and PUP-4 target specificity might reflect, in part, a preference for the last 3′ templated nt on a sRNA as has been shown for *Drosophila* Tailor (Bortolamiol-Becet *et al.* 2015; Reimão-Pinto *et al*. 2015). We identified the 3′ templated nt of wild-type U-tailed sRNAs and parsed the sRNAs by the PUP(s) responsible for their modification (Supplementary Fig. 7). Overall, the most frequent final templated nt is U (∼30%) and least frequent is A (∼20%), a pattern that holds for targets of each PUP across most or all of their activity ranges (Supplementary Fig. 7). Subtle shifts are present for PUP-4 at both ends of the activity range and among sRNAs whose U-tailing is limited by PUP-2 or PUP-3 (Supplementary Fig. 7). Overall, a 3′ nt preference is not a major factor in PUP sRNA target choice.

### The relationship between sRNA abundance level and U-tail frequency

**Fig. 3.**
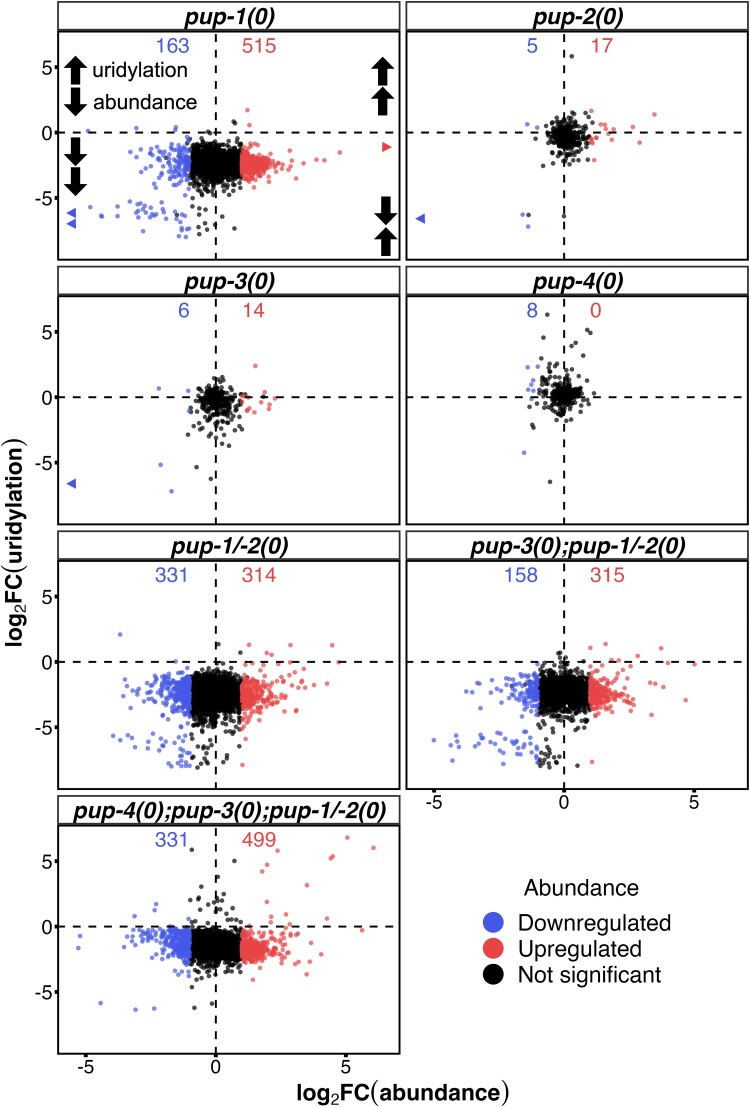
Loss of PUP activity profoundly alters sRNA abundance. All points represent sRNAs whose U-tailing is consistently reduced in the indicated mutant (high-confidence uridylation targets). Differentially abundant sRNAs have a |log_2_FC| > 1 and FDR < 0.05. The number of DA sRNAs is listed for each genotype. *X*-axis, log_2_FC of abundance (mutant/wild type). *Y*-axis, log_2_FC of uridylation frequency (mutant/wild type) (see *Materials and methods*). Triangles, indicating sRNAs with log_2_FC(abundance) values <|6|, are plotted at their corresponding log_2_FC(uridylation) value.

Based on the DA patterns we see upon uridylation loss, U-tailing appears to promote turnover of certain sRNAs (red points) and limit turnover of others (blue points) while not significantly affecting abundance of yet others (black points) (Fig. 3). *pup-1(0)* mutants had the largest number of DA sRNAs of any single mutant strain (515 up, 163 down) (Fig. 3) while *pup-3(0); pup-4(0); pup-1/-2(0)* quadruple mutants had the largest number of DA sRNAs in any strain we evaluated (499 up, 331 down) (Fig. 3). Although the mildly elevated U-tailing in *pup-4* single mutants rarely correlated with DA of those sRNAs, the more substantially increased U-tailing in *pup-3(0); pup-4(0); pup-1/-2(0)* animals tended to correlate with increased abundance (Fig. 3). These results reinforce the hypothesis that 3′ uridylation has context-dependent functions with respect to sRNA abundance.

### sRNAs associated with specific Argonaute proteins

**Fig. 4.**
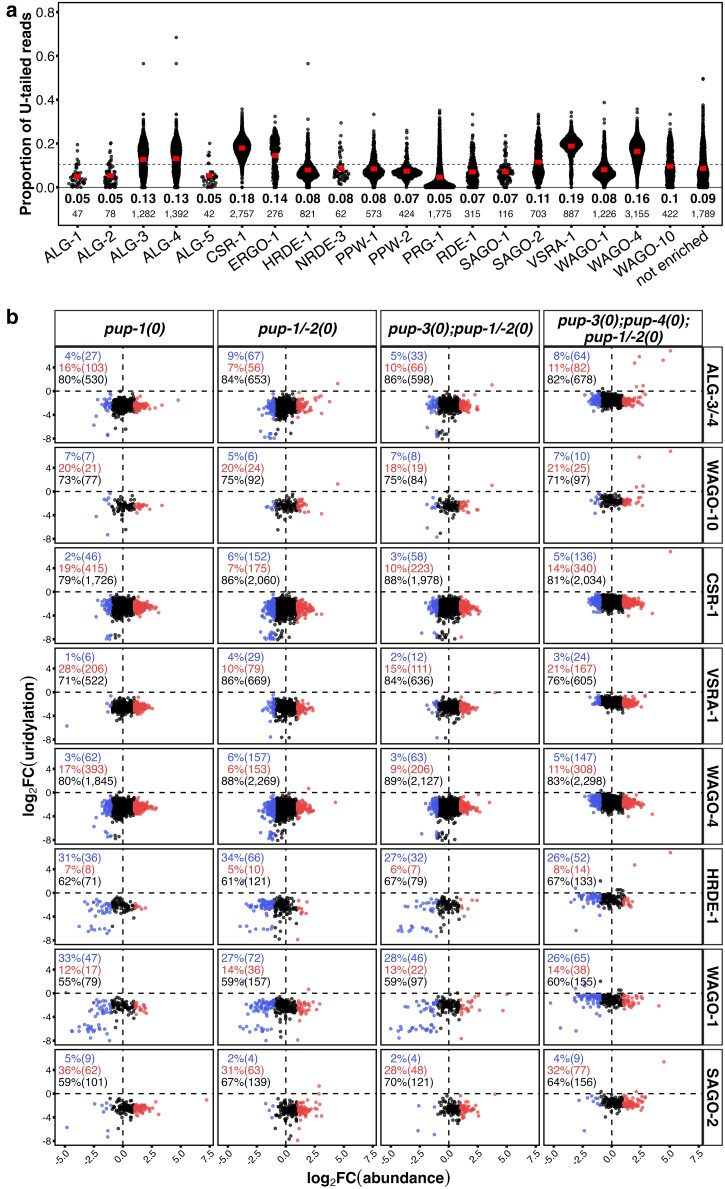
Changes in sRNA abundance parsed by Argonaute association. a) U-tail frequencies for sRNAs present at ≥10 CPM in wild-type adult hermaphrodites, sorted based on documented associations with individual Argonaute proteins (Seroussi *et al.* 2023). *X*-axis, Argonaute proteins. *Y*-axis, proportion of U-tailed reads for corresponding sRNAs in our wild-type data set. Each point represents an sRNA. sRNAs that associate with multiple Argonautes are represented in each Argonaute group. “Not enriched,” sRNAs without a reported Argonaute preference. Dashed line, average uridylation frequency (11%) for the 9,645 Argonaute-associated sRNAs in wild type. Bar, average U-tail frequency for each group of Argonaute-associated sRNAs; this value and number of sRNAs associated with each Argonaute are listed below. b) High-confidence uridylated sRNAs that are DA in the indicated genotypes are plotted by Argonaute association. *X*-axis, log_2_FC of abundance (mutant/wild type). *Y*-axis, log_2_FC of uridylation frequency (mutant/wild type). Values are listed in each panel. |log_2_FC| > 1 and FDR < 0.05. See key in Fig. 3. Remaining Argonaute associations are shown in Supplementary Fig. 10.

sRNAs classically regulate gene expression by interacting with and guiding specific Argonaute proteins to RNA targets (Billi *et al.* 2014). Argonaute activity is primarily implicated in limiting gene expression, although CSR-1 Argonaute is associated with licensing gene expression (Billi *et al.* 2014; Almeida *et al.* 2019). Taking advantage of recent systematic identification of Argonaute-associated sRNAs in *C. elegans*, we parsed sRNAs in our data sets by their known Argonaute associations in young adult hermaphrodites (Seroussi *et al*. 2023) (Fig. 4). Average uridylation frequency reflects (in part) the relative proportion of siRNAs vs miRNAs/piRNAs that associate with an Argonaute, as expected since siRNAs are U-tailed at a higher frequency than miRNAs and piRNAs. In our wild-type data set, U-tail frequency is highest for VSRA-1 (19%), CSR-1 (18%), and WAGO-4 (16%) associated sRNAs (mostly siRNAs), with a wide range of values around each average (Fig. 4a; sRNAs present at ≥10 CPM). To identify Argonautes whose activity might be especially altered in the absence of PUP-1, we evaluated DA of high-confidence uridylated sRNAs, parsed by Argonaute association, in strains carrying *pup-1(0)* (Fig. 4b; Supplementary Fig. 10). In terms of numbers of sRNAs affected, reduced U-tailing correlated most strongly with increased abundance of sRNAs associated with VSRA-1, CSR-1, and WAGO-4, all germline expressed Argonautes targeting protein-coding RNAs (Fig. 4b). Although the numbers are smaller, a similar percentage of sRNAs increased in abundance for ALG-3/-4 and WAGO-10 (known to function in spermatogenesis), SAGO-2 (soma-enriched expression), and ERGO-1 (functions in early embryogenesis) (Fig. 4b; Supplementary Fig. 10). In contrast, reduced U-tailing correlated with decreased abundance of subsets of sRNAs associated with HRDE-1 and WAGO-1 (germline-enriched expression, target nonprotein-coding RNAs) (Fig. 4b). Overall, our data suggest that uridylation may increase or decrease sRNA abundance depending on the specific Argonaute involved and its functional context.

### Misregulated sRNAs preferentially target genes enriched for germline and ubiquitous expression

**Fig. 5.**
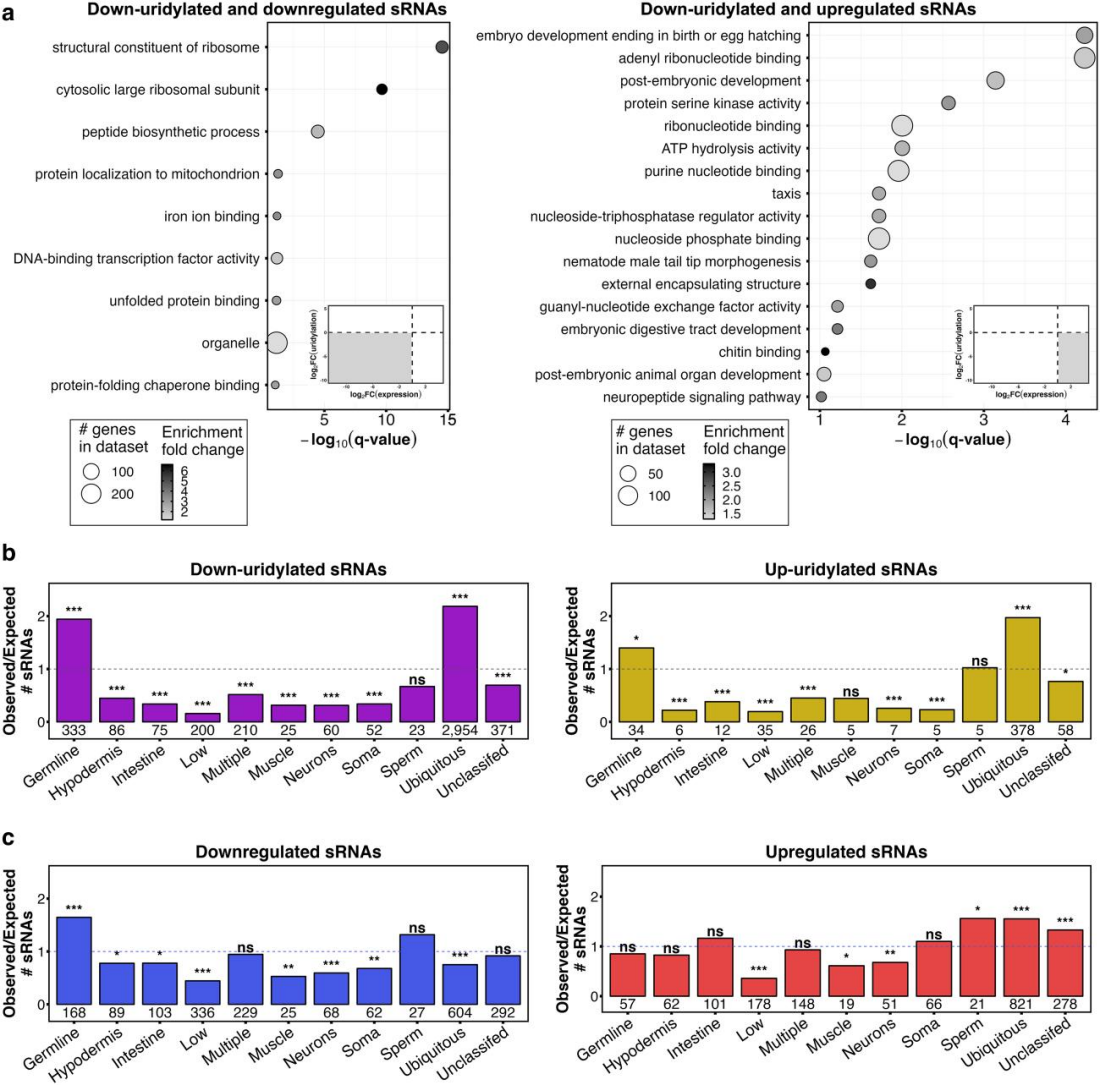
The mRNA targets of high-confidence uridylated sRNAs and differentially abundant sRNAs have primary expression in the germline or ubiquitous expression. a) GO analysis of mRNAs targeted by high-confidence U-tailed sRNAs that are differentially abundant in any *pup* mutant. Left, genes targeted by reduced abundance sRNAs (636) encode products enriched for 9 GO terms. Right, genes targeted by increased abundance sRNA (779) encode products enriched for 17 GO terms. Inserts indicate the quadrant in Fig. 3 plots corresponding to the differentially abundant sRNAs used in the GO analysis. b) Tissue expression patterns for genes targeted by sRNAs with reduced (4,609, left) or increased (654, right) uridylation in any *pup* mutant (regardless of abundance). c) Tissue expression patterns for genes targeted by differentially abundant sRNAs in any mutant with reduced (2,745, left) or increased (2,548, right) abundance as represented in Supplementary Fig. 8 (regardless of uridylation). Tissue expression data are from Serizay *et al.* (2020). Tissue specificity index was calculated based on cummeRbund's S function; *χ*^2^ test (*P < 0.01, **P < 0.001, ***P < 0.0001) was used to calculate significance. ns, not significant.

Misregulation of sRNAs may contribute to the pleiotropic developmental phenotypes observed in *pup* mutants. We investigated this question by examining the biological functions and tissue expression patterns of the targets of sRNAs shown in Fig. 3. GO analysis identified distinct functional classes among genes targeted by DA high-confidence uridylated sRNAs (Fig. 5a). Targets of sRNAs with reduced uridylation and abundance in any *pup* mutant are enriched for 9 GO terms; the top 2 include structural components of ribosomes. In contrast, targets of sRNAs with reduced uridylation and elevated abundance are enriched for numerous GO categories, including several terms involved with embryonic and postembryonic development and ribonucleotide and nucleotide biochemistry. We hypothesize that PUP activity functions in regulating genes with important functions in the germline development and, by extension, embryogenesis.

We investigated tissue expression using the Serizay *et al.* (2020) database where genes are classified based on enriched expression in individual tissues and combinations of tissues in late L4—young adult stage wild-type animals. Targets of sRNAs shown in Fig. 3 tend to have either maximum expression in the germline or ubiquitous expression, a pattern that holds for sRNAs with either reduced or increased U-tailing in *pup* mutants (Fig. 5b). Nearly all other tissue expression categories are significantly underrepresented among these sRNAs. We also analyzed tissue expression of genes that are targets of DA sRNAs regardless of uridylation status (see Supplementary Fig. 8); downregulated sRNAs preferentially target genes with maximal expression in the germline, and upregulated sRNAs preferentially target genes with ubiquitous expression or expression in the “sperm” category (Fig. 5c). Interestingly, the ubiquitous category includes *msp* and some other genes that are highly expressed in sperm. Several other tissue expression categories are underrepresented in the DA data. Together, these results indicate that U-tailing likely impacts expression of genes with enriched expression in the germline and broad somatic expression in the adult hermaphrodite.

### mRNA U-tailing and *pup* mutants

**Fig. 6.**
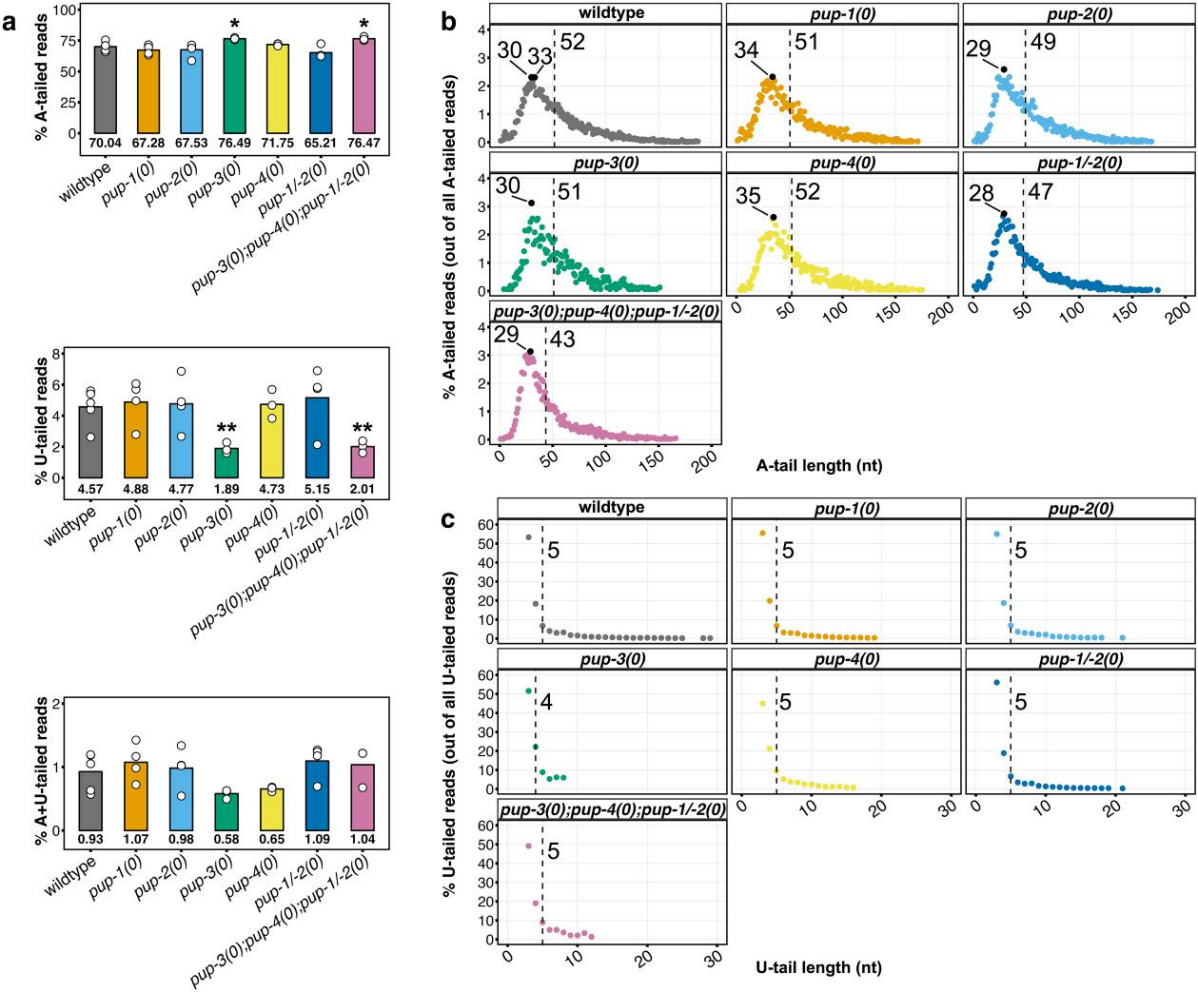
mRNA U-tail frequency and length are reduced in *pup-3(0)* mutants. a) mRNA 3′ tail frequencies in wild-type and *pup* mutant adults as detected with *tailfindr* analysis of Nano3P-seq data. Plots indicate the average % of strictly A-tailed reads, strictly U-tailed reads, and A + U-tailed reads out of the total number of reads obtained. White points, average % tailed reads for each replicate. *Y*-axis, % tailed reads; *X*-axis, genotype. Statistical significance for each mutant compared to wild type was determined by paired *t*-test (*P < 0.02, **P < 0.01). b and c) The distribution of A- and U-tail lengths in wild-type and *pup* mutants. Each point represents the average tailing frequency of all mRNAs with tails of a certain length. For the 4,183 transcripts present at ≥10 CPM, a cutoff of 50 reads/length group was implemented. *X*-axes, tail length in nucleotides. *Y*-axes, percentage of tailed reads. Dashed line/number, average tail length. Black points, most common tail length(s). c) Although 3 nt is the most common U-tail length detected for any genotype, note that *tailfindr* cannot identify shorter U-tails (see text).

To identify U-tailed mRNAs and compare 3′ tailing among *pup* mutants, we chose Nano3P-seq, an ONT-based method (Begik *et al.* 2023). Although ONT is a lower throughput platform than Illumina (library sizes in the order of 10^5^ vs 10^6^), the method offers flexibility in library preparation (see *Materials and methods*). We constructed libraries using RNA isolated from synchronized populations of wild-type and *pup* mutant adult hermaphrodites of the same age as for sRNA-seq. Nontemplated 3′ nucleotides were identified using a modified version of *tailfindr* software (Krause *et al.* 2019) that can distinguish strings of nontemplated A, U, and A followed by U (designated A + U) residues on mRNA 3′ ends (see *Materials and methods*). This approach identified strict A-tails on ∼72% of mRNAs, strict U-tails on ∼3.4% of mRNAs, and A + U tails on ∼0.93% of mRNAs (Fig. 6a). The strict U-tails were not limited to histone mRNAs, the major class of mRNAs known to lack poly(A) tails. Importantly, limitations on current ONT technology are that *tailfindr* cannot distinguish stretches of fewer than 3 Us or intermixed As and Us within a tail. Hence, our analysis almost certainly undercounts 3′ tails containing U residues, especially runs of A + U. Nonetheless, to our knowledge, our data demonstrate the first direct report of mRNA U-tailing in *C. elegans*.

Nano3P-seq identified changes in A- and U-tailing in *pup-3(0)* single and *pup-3(0); pup-4(0); pup-1/-2(0)* quadruple mutants compared to wild type (Fig. 6a). The frequency of strict A-tailed reads increased from ∼72% in wild type to ∼78% in both *pup-3(0)* and *pup-3(0); pup-4(0); pup-1/-2(0)* quadruple mutants, and the frequency of strict U-tailed reads decreased from ∼3.4% in wild type to ∼1.5% in both mutants. A + U-tailing was similar in wild-type and all *pup* mutants (∼1%) except *pup-3(0)* mutants (∼0.58%). The increased frequency of strict A-tails in the absence of PUP-3 may reflect loss of U residues from intermixed A/U tails, which are not detected by *tailfindr*, as well as loss of strict U-tails. Overall, these results are consistent with PUP-3 functioning to uridylate nonadenylated mRNAs.

We observed mild effects on mRNA A- and U-tail lengths in *pup* mutants. The average strict A-tail length in our wild-type mRNA data set was ∼51 nt, and the most common A-tail lengths were 30 and 33 nt (Fig. 6b). Strict A-tails were shorter than or the same length as wild type in all *pup* mutant strains (averaging 43–51 nt), and the largest effect on tail length was observed in the quadruple *pup* mutant where average tail length was 43 nt. The average strict U-tail length in the wild-type mRNA data set was ∼5 nt, and the most common length was 3 nt—the shortest U-tail that *tailfindr* reliably detects (Fig. 6c). Average strict U-tail length did not differ significantly between wild type and any *pup* mutant. Uncommon, long U-tails identified in wild type were notably less frequent in all *pup* mutants and particularly in *pup-3(0)* and *pup-4(0)* single mutants and the *pup-3(0); pup-4(0); pup-1/-2(0)* quadruple mutant. Overall, loss of PUP-3 is associated with subtle changes in tail length.

### Differential mRNA abundance

**Fig. 7.**
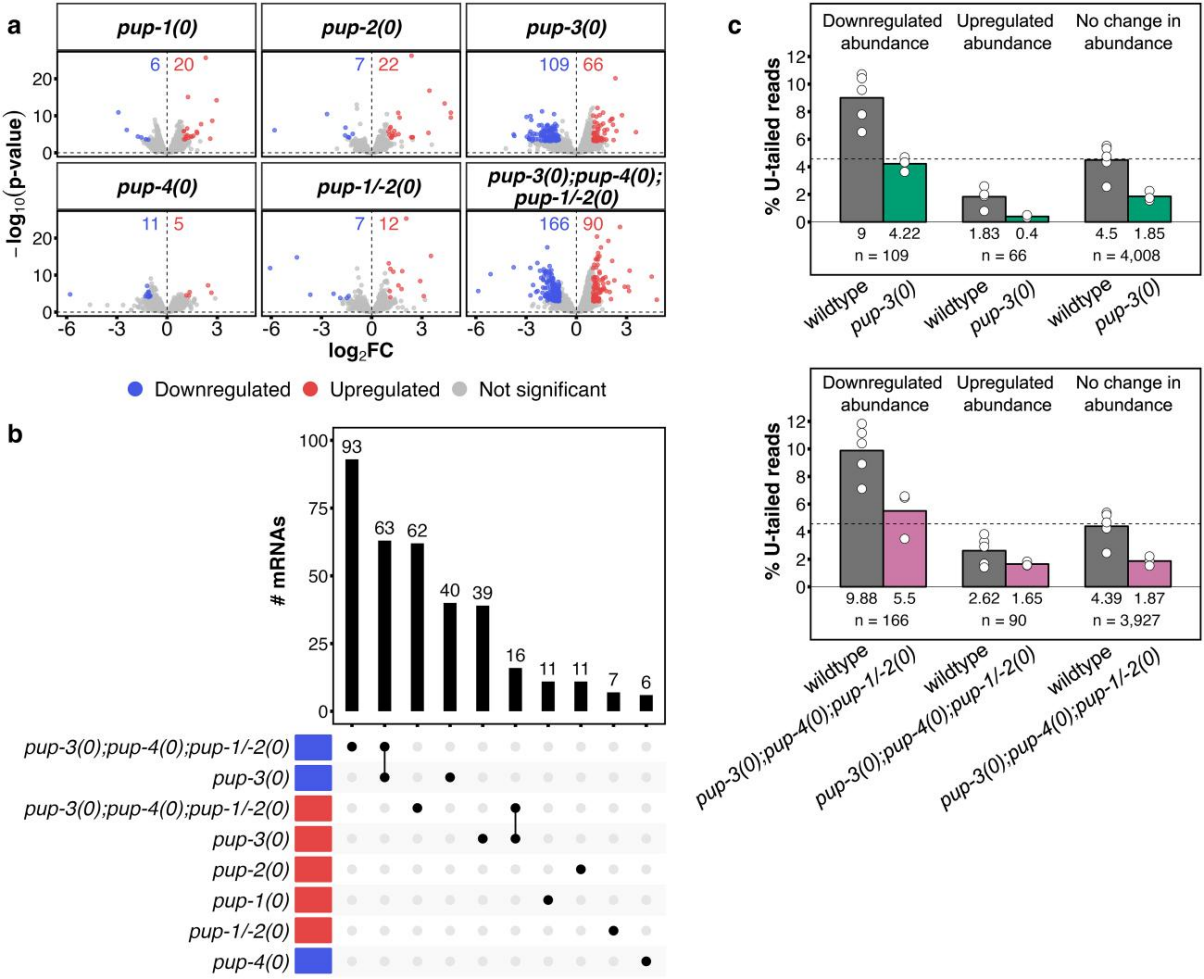
Differentially abundant mRNAs are most prominent in the *pup-3(0); pup-4(0); pup-1/-2(0)* quadruple mutant. a) Plots show DA mRNAs detected in each genotype. Each point represents mRNA from a single gene. Numbers of DA mRNAs are provided. *X*-axis, log_2_FC in mutant vs wild type. *Y*-axis, −log_10_(*P*-value). Statistical significance was determined as |log_2_FC| > 1 and FDR < 0.01. b) UpSet plot shows numbers of unique and shared differentially abundant mRNAs identified in *pup* mutant strains; the top 10 intersections are shown (out of 32). c) Bar plots indicate the % U-tailed reads for mRNAs that are differentially abundant in (upper) *pup-3(0)* single or (lower) *pup-3(0); pup-4(0); pup-1/-2(0)* quadruple mutant compared to wild type. *n*, number of mRNAs in each group. Average % U-tailing is indicated under each bar. Dashed line, average % U-tailing in wild type from Fig. 6a (second panel) for the 4,183 transcripts present at ≥10 CPM.

Differentially expressed mRNAs are difficult to identify reliably with ONT data because the read depth is low. To verify that our data set had sufficient depth coverage, we compared our Nano3P-seq data with the top 100 highly expressed genes in *C. elegans*, based on Illumina RNA-seq data from 5 tissues (Serizay *et al.* 2020). These 100 genes were all represented in our wild-type replicates, suggesting we had adequate coverage for downstream analysis (see Supplementary File 3). Using conventional edgeR analysis and a cutoff of 2-fold difference between *pup* mutant and wild type, we identified 592 mRNAs as differentially abundant in at least 1 *pup* mutant strain (Fig. 7a) (see *Materials and methods*). Most differentially abundant mRNAs were observed in *pup-3(0); pup-4(0); pup-1/-2(0)* quadruple mutants (166 down, 90 up) and/or *pup-3(0)* single mutants (109 down, 66 up) (Fig. 7a and b). We hypothesize that differential mRNA abundance in *pup* mutants reflects not only any direct impact of reduced U-tailing but also changes in abundance and uridylation of sRNAs whose activity impacts mRNA abundance. Moreover, since mRNAs are templates for siRNA production, it is likely that changes in mRNA abundance contribute to changes in sRNA levels. Consistent with this relationship, changes in sRNA abundance are more extensive in the *pup-3(0); pup-4(0); pup-1/-2(0)* quadruple mutant than in the *pup-3(0)* single mutant (Fig. 3).

Among differentially abundant mRNAs in *pup-3(0)* and *pup-3(0); pup-4(0); pup-1/-2(0)* mutants, the average strict U-tailing frequency is reduced for both down- and upregulated transcripts. For example, in *pup-3(0)* mutants, the average U-tailing drops from ∼9 to ∼4% among downregulated mRNAs and from ∼2 to ∼0.4% among upregulated mRNAs (Fig. 7c). A similar pattern holds in *pup-3(0); pup-4(0); pup-1/-2(0)* mutants (Fig. 7c). Interestingly, average U-tailing among these DA transcripts is distinct from the average U-tailing of mRNAs overall, i.e. downregulated mRNAs are uridylated at higher frequency than average in wild type, and upregulated mRNAs are uridylated at a lower frequency than average in wild type (Fig. 7c). Considering that U-tail loss correlates with reduced, increased, or unchanged mRNA abundance, PUP-3 activity appears to positively or negatively regulate subsets of mRNA targets depending on context.

Among the 592 DA mRNAs captured by Nano3P-seq, ∼10% (61) correspond to DA sRNAs reported here for the same mutant to wild type comparison. For ∼4% (23) of these cases, the sRNA and mRNA change in the same direction (both up or both down). In addition, ∼24% (144) of the DA mRNAs correspond to high-confidence uridylated sRNAs in the same mutant to wild type comparison. These data are consistent with changes in sRNA abundance and sRNA uridylation in *pup* mutants contributing to changes in mRNA abundance. When mRNA and sRNA abundance changes in parallel, this may reflect altered mRNA template availability contributing to altered sRNA production.

## Discussion

While RNA uridylation has recently been established as critical for germline and embryonic development in *C. elegans* and other species, much less is known about the specific RNAs that are marked by uridylation and how their abundance may be altered by that modification. Here, we provide a resource for uncovering the importance of U-tailing in regulating *C. elegans* gene expression. Our data indicate that the 4 known *C. elegans* poly(U) polymerases promote tailing of sRNAs in the adult hermaphrodite; siRNAs are the primary targets, and PUP-1 is primarily responsible for uridylation activity. PUP-2, PUP-3, and PUP-4 have fewer targets in these animals.

Several observations suggest a complex relationship among PUP enzymes in vivo. The increased tailing of certain sRNAs in *pup-2(0)*, *pup-3(0)*, and especially *pup-4(0)* mutants suggests that these 3 enzymes may limit U-tailing—perhaps by PUP-1—in at least some tissues. This may occur, for example, if they have lower activity than PUP-1 and delay its ability to access target RNAs. Another intriguing observation is that U-tailing is not completely abolished in the *pup-3(0); pup-4(0); pup-1/-2(0)* mutant, and therefore, another TENT presumably has 3′ uridylation activity in the absence of the PUPs. Studies in *Schizosaccharomyces pombe* and HEK293T cells similarly have observed residual uridylation in the absence of poly(U) polymerase activity, suggesting the work of another non-PUP/TUTase in those systems (Chung *et al.* 2019; Yang *et al.* 2022). Although in vitro evidence suggests many *C. elegans* TENTs preferentially add only A or U, Preston *et al.* (2019) observed a low level of nonpreferential tailing by some TENTs, as well as TENTs that commonly add more than 1 nucleotide type. Another consideration is that Preston *et al.* (2019) assayed U-tailing activity in a situation where a single TENT was expressed, and therefore, any interaction or interference among different TENTs would not have been observed.

### Developmental implications of PUP activity

Uridylation is implicated as regulating RNA stability by signaling turnover of mRNA and some sRNAs (Heo *et al.* 2008; Rissland and Norbury 2009; van Wolfswinkel *et al.* 2009; Ibrahim *et al.* 2010; Ren *et al.* 2012; Lim *et al.* 2014; Kim *et al.* 2015; Morgan *et al.* 2017, 2019; Chang *et al.* 2018; Yang *et al.* 2020, 2022), restabilizing mRNAs (e.g. in *Arabidopsis*) (reviewed by Łabno *et al.* 2016), and allowing mRNAs to remain dormant (e.g. in starfish oocytes and early embryos) (Ochi and Chiba 2016). Our data suggest that sRNA U-tailing in *C. elegans* promotes stability of some sRNAs and limits stability of others. Positively regulated sRNAs are predicted to target mRNAs encoding structural components of ribosomes or proteins otherwise associated with peptide biosynthesis. We find that sRNAs targeting ribosomal protein genes are also uridylated, and these sRNAs become less abundant in the absence of U-tailing. Previous work showed that *C. elegans* ribosomal siRNAs (risiRNAs), rRNAs, and sense rRNA fragments can be uridylated by PUP-1 and/or PUP-2 (Zhou *et al.* 2017; Wang *et al.* 2020; Wahba *et al.* 2021). Together, these data suggest uridylation functions in regulating the handoff from maternal to embryonic ribosomes. Negatively regulated sRNAs, in contrast, are predicted to target genes with a wider range of products including many linked to embryonic and postembryonic development. Since our sRNA samples were obtained from gravid adult hermaphrodites, we expect them to include oocyte sRNAs that direct gene expression in the early embryo. Uridylation that modulates stability of these sRNAs may promote the transition to embryonic gene expression.

One goal of this work was to gain insight into the *pup* mutant phenotype (Li and Maine 2018; Li *et al.* 2021). The profound effects on sRNA abundance in strains carrying *pup-1(0)* are likely to alter developmental gene expression, and subtle phenotypic differences in strains carrying mutations in 1 or more additional *pup* genes may reflect differences in sRNA U-tailing and abundance among these strains. In addition, direct effects on mRNA abundance are likely to feedback on sRNA production from mRNA templates. sRNA comparison with Illumina mRNA-seq data shows ∼66% of overlapping DA sRNAs and mRNA change abundance in the same direction (both increase or both decrease), consistent with feedback (Kelley LH and Maine EM, unpublished data). Relevant for understanding the complex PUP phenotypes, GO and tissue specificity analyses identified targets of misregulated sRNAs as genes with germline-enriched or ubiquitous expression whose products function in developmental processes and ribosome biogenesis. Future analysis of PUP targets at other developmental stages and in somatic tissues, e.g. individual neurons, may identify additional targets. *pup* mRNAs are expressed in multiple somatic cells and tissues, and numerous tissues express *pup-2*, *pup-3*, and/or *pup-4* but not *pup-1* mRNA (Hammarlund *et al*. 2018). The high proportion of germline sRNAs in our adult data sets may obscure important PUP-2, PUP-3, and PUP-4 functions in somatic cells and tissues.

Uridylation tends to correlate negatively with abundance of many sRNAs associated with CSR-1, VRSA-1, WAGO-4, ALG-3/-4, and SAGO-2—all of which have relatively high U-tailing frequency. In contrast, uridylation tends to correlate positively with abundance of many sRNAs associated with HRDE-1 and WAGO-1—both of which have relatively lower tailing frequency. These data suggest that uridylation may have different consequences for sRNA stability depending on the associated Argonaute proteins. Previous reports have implicated uridylation as modulating the interaction of shared sRNAs with competing Argonautes CSR-1 and WAGO-4 (de Albuquerque *et al.* 2015; Phillips *et al.* 2015; Xu *et al.* 2018). Our data are consistent with the model that uridylation can stabilize or destabilize sRNAs depending on their interacting Argonaute regardless of competition.

### mRNA uridylation in *C. elegans*

We identified a role for PUP-3 in adding 3′ uridine to nonpolyadenylated mRNAs in the adult hermaphrodite. Although our mRNA analysis was limited by technical issues, the data are consistent with PUP-3 having a distinct role in mRNA U-tailing not shared by other PUPs. The frequency of strict A-tails increased, and frequency of strict U-tails decreased in the absence of PUP-3. Tailing was not further altered in the *pup-3(0); pup-4(0); pup-1/-2(0)* mutant, consistent with single mutant data indicating that PUP-1, PUP-2, and PUP-4 are not individually essential for uridylating nonadenylated mRNAs. Nonetheless, changes in mRNA abundance are more pronounced in *pup-3(0); pup-4(0); pup-1/-2(0)* mutants than *pup-3(0)* mutants, perhaps due to major changes in the sRNA population in *pup-3(0); pup-4(0); pup-1/-2(0)* compared to *pup-3(0)* animals and/or reduced mRNA uridylation in the absence of PUP-1, PUP-2, and/or PUP-4 that we cannot detect with current *tailfindr* software. We look forward to future technical refinements that will allow better identification of mixed poly(A)+poly(U) tails and the PUP protein(s) responsible for their formation.

Overall, our data reveal changes in uridylation and composition of the *C. elegans* small RNAome and transcriptome in the adult hermaphrodite upon loss of individual and multiple poly(U) polymerases. Identification of PUP RNA targets and relative contributions of different PUPs to RNA modification provides a resource for future work investigating PUP activity in individual cells and tissues.

## Supplementary Material

iyae120_Supplementary_Data

## Data Availability

sRNA-seq and Nano3P-seq data sets are deposited in NCBI Gene Expression Omnibus (GEO) as GSE271566 and GSE271568. Supplemental material available at GENETICS online.
