# Supplementary material for: Poly(U) polymerase activity in Caenorhabditis elegans regulates abundance and tailing of sRNA and mRNA
Source: Genetics. 2024 Jul 28;228(2):iyae120. doi: 10.1093/genetics/iyae120 (PMC11457939; doi:10.1093/genetics/iyae120)
Supplement: iyae120_Supplementary_Data [file iyae120_supplementary_data.zip › File_S1_GENETICS-2024-307061.docx]

**Supplemental Methods**

**Bioinformatic analysis of sRNAs**

3’ adapters (‘5-AGATCGGAAGAGCACACGTCTGAACTCCAGTC-3’) were trimmed with cutadapt (RRID:SCR_011841); reads spanning 15-26 nt with a 3’ quality cutoff of 20 were retained using FastQC (RRID:SCR_014583) (Andrews 2010) and mapped to the *C. elegans* WS284 reference genome using HISAT2 (RRID:SCR_015530) (Kim *et al.* 2019). The resulting SAM files were converted to BAM and sorted using SAMTOOLS (RRID:SCR_002105) (Li *et al.* 2009). Small RNAs and their 3’ non-templated tails were identified with our smalldisco pipeline (Caldas *et al.* 2023; github.com/ianvcaldas/smalldisco). In brief, using the *C. elegans* WS272 canonical geneset GTF, miRNAs were selected from the ninth column (gene_biotype) and converted into a BED file using BEDOPS’ *gtf2bed* (RRID:SCR_012865) (Neph *et al.* 2012). This process was repeated for piRNAs. Using the tail command in the smalldisco pipeline, 3’ tails on miRNAs and piRNAs were identified using the sense and antisense read parameter (Chou *et al.* 2015). For siRNAs, the sirna mode in smalldisco was used to identify reads that mapped antisense to CDS regions; we defined siRNAs by the gene to which they map antisense. This list of siRNAs was input for the smalldisco tail mode to identify 3’ tails, using the antisense only parameter.

For the sRNA abundance cutoff in our uridylation analysis, we selected sRNAs with a minimum count of 50 CPM in a worthwhile number of samples using edgeR::filterByExpr() with a min.count = 50 (RRID:SCR_012802) (Robinson *et al.* 2010), unless otherwise noted.

For transposon analysis, sRNA-seq libraries were mapped using STAR (RRID:SCR_004463) (Dobin *et al.* 2013) with parameters --outFilterMultimapNmax 100 and --winAnchorMultimapNmax 100 to account for multi-mapping reads. Mapped reads and a transposon GTF (*ce11_rmsk_TE.gtf*;<https://labshare.cshl.edu/shares/mhammelllab/www-data/TEtranscripts/>, compiled by Molly Hammell’s lab) were supplied to smalldisco to identify siRNAs that map to transposons.

**Nano3P-seq, reagents, and data analysis**

To calibrate *tailfindr* to distinguish poly(U) and poly(A)+poly(U) from poly(A) sequences, we generated tail standards containing 3’ poly(U) or poly(A)+poly(U) sequences of different lengths (Krause *et al.* 2019). We constructed a series of plasmids containing a common 5’ sequence (~480 nt) obtained from plasmid pJJR82 (RRID:Addgene_75027) to act as a mock mRNA with varying 3’ sequences with different numbers of As, Ts, and A+Ts. We designed a forward primer complementary to pJJR82 that contained a restriction site and a series of reverse primers containing sequences complementary to pJJR82, the mock “tail,” and a restriction site. Long reverse primers were obtained from Invitrogen (25 nmole, single-stranded) or IDT (4 nmole Ultramer DNA Oligo, single-stranded) for a total of 11 tail standards (Files S1, S2). Amplification reactions were performed with Q5 High-Fidelity DNA Polymerase. A single 3’ adenine was added to purified amplification products using Taq; products were ligated to pGEM-T vector and transformed into DH5-alpha *E. coli*. Colony PCR was used to identify successfully ligated products; plasmids were purified, and their sequences verified with Sanger sequencing. To isolate sequences for *tailfindr* calibration, the mock RNA + tail sequences were excised from their plasmid backbone using the terminal restriction sites, and single-stranded ends were removed with mung bean nuclease (NEB, M0250S). Excised fragments (tail standards) were gel purified for sequencing with the ONT Ligation Sequencing Kit (SQK-LSK110). All 11 standards were pooled into one sequencing run. Sequencing data were used to train *tailfindr* to detect poly(U) and poly(A)+poly(U) tails. See supplementary File S2 for tail standard sequences and File S3 for our synthesis protocol.

To generate libraries for mRNA sequencing, rRNA was depleted from total RNA preparations using an RNase H method designed for *C. elegans* (Duan *et al.* 2020). For each replicate, rRNA was depleted from 10 µg of total RNA, which yielded 100-200 ng of rRNA-depleted RNA to use for library preparation.  See supplementary File S4 for our modified library preparation protocol.

FAST5 files were basecalled using *guppy_basecaller*. No trim strategy was specified to retain the 3’ adapter for *tailfindr* to identify 3’ tails (Krause *et al.* 2019; Niazi *et al.* 2021). Sequence quality was assessed with NanoPlot (RRID:SCR_024128) (De Coster *et al.* 2018). FAST5 files were demultiplexed with ONT FAST5 API software using the *demux_fast5* command (https://github.com/nanoporetech/ont_fast5_api) and were used as input to identify tails using *tailfindr*. To annotate reads, FASTQ files were demultiplexed with *guppy_barcoder* and mapped to the *C. elegans* transcriptome (RefSeq GCF_000002985.6) using Minimap2 (RRID:SCR_018550) (Li 2018). These SAM files and the results from the tail identification step were used to annotate the tails by gene. Details of the *tailfindr* pipeline can be found on GitHub under the polyu branch (https://github.com/adnaniazi/tailfindr/tree/polyu). The resulting counts tables were used for analysis. For differential abundance analysis, mRNAs that passed the edgeR::filterByExpr() function with a min.count = 10 were used (Robinson *et al.* 2010).

List of packages and versions:

cutadapt

FastQC

HISAT2

SAMTOOLs

BEDOPS

edgeR (v3.36.0)

STAR (v2.7.9)

guppy_basecaller (v4.4.2)

NanoPlot (v1.32.1)

ONT FAST5 API software, *demux_fast5* (v4.1.1)

*guppy_barcoder* (v4.4.2)

Minimap2 (v2.17)

EDASeq (v2.28.0)

RUVseq (v1.28.0)

**Supplemental Literature cited**

Andrews S. 2010. FastQC: a quality control tool for high throughput sequence data.<https://www.bioinformatics.babraham.ac.uk/projects/fastqc/>.

Caldas IV, Kelley LH, Ahmed-Braimah YH, Maine EM. 2023. smalldisco, a pipeline for siRNA discovery and 3′ tail identification. G3. 13:jkad092.doi:10. 1093/g3journal/jkad092.

Chou M-T, Han BW, Hsiao C-P, Zamore PD, Weng Z, Hung J-H. 2015. Tailor: a computational framework for detecting non-templated tailing of small silencing RNAs. Nucleic Acids Res. 43:e109. doi:10.1093/nar/gkv537.

De Coster W, D’Hert S, Schultz DT, Cruts M, Van Broeckhoven C. 2018. NanoPack: visualizing and processing long-read sequencing data. Bioinformatics. 34:2666–2669. doi:10.1093/bioinformatics/bty149.

Dobin A, Davis CA, Schlesinger F, Drenkow J, Zaleski C, Jha S, Batut P, Chaisson M, Gingeras TR. 2013. STAR: ultrafast universal RNA-seq aligner. Bioinformatics. 29:15–21. doi:10.1093/bioinformatics/bts635.

Duan Y, Sun Y, Ambros V. 2020. RNA-seq with RNase H-based ribosomal RNA depletion specifically designed for *C. elegans*. MicroPub Biol. doi:10.17912/micropub.biology.000312.

Kim D, Paggi JM, Park C, Bennett C, Salzberg SL. 2019. Graph-based genome alignment and genotyping with HISAT2 and HISAT-genotype. Nat Biotech. 37:907–915. doi:10.1038/s41587-019-0201-4.

Krause M, Niazi AM, Labun K, Cleuren YNT, Müller FS, Valen E. 2019. *tailfindr*: alignment-free poly(A) length measurement for Oxford Nanopore RNA and DNA sequencing. RNA. 25:1229–1241. doi:10.1261/rna.071332.119.

Li H. 2018. Minimap2: pairwise alignment for nucleotide sequences. Bioinformatics. 34:3094–3100. doi:10.1093/bioinformatics/bty191.

Li H, Handsaker B, Wysoker A, Fennell T, Ruan J, Homer N, Marth G, Abecasis G, Durbin R, 1000 Genome Project Data Processing Subgroup. 2009. The Sequence Alignment/Map format and SAMtools. Bioinformatics. 25:2078–2079. doi:10.1093/bioinformatics/btp352.

Neph S, Kuehn MS, Reynolds AP, Haugen E, Thurman RE, Johnson AK, Rynes E, Maurano MT, Vierstra J, Thomas S, Sandstrom R, Humbert R, Stamatoyannopoulos JA. 2012. BEDOPS: high-performance genomic feature operations. Bioinformatics. 28:1919–1920. doi:10.1093/bioinformatics/bts277.

Niazi AM, Krause M, Valen E. 2021. Transcript isoform-specific estimation of poly(A) tail length by Nanopore sequencing of native RNA. Bioinformatics, Methods in Molecular Biology. New York, NY: Springer US. pp. 543–567. doi:10.1007/978-1-0716-1307-8_30.

Robinson MD, McCarthy DJ, Smyth GK. 2010. edgeR: a Bioconductor package for differential expression analysis of digital gene expression data. Bioinformatics. 26:139–140. doi:10.1093/bioinformatics/btp616.

Vieux K-F, Prothro KP, Kelley LH, Palmer C, Maine EM, Veksler-Lublinsky I, McJunkin K. 2021. Screening by deep sequencing reveals mediators of microRNA tailing in *C. elegans*. Nucleic Acids Res. 49:11167–11180. doi:10.1093/nar/gkab840.
