# Supplementary material for: Poly(U) polymerase activity in Caenorhabditis elegans regulates abundance and tailing of sRNA and mRNA
Source: Genetics. 2024 Jul 28;228(2):iyae120. doi: 10.1093/genetics/iyae120 (PMC11457939; doi:10.1093/genetics/iyae120)
Supplement: iyae120_Supplementary_Data [file iyae120_supplementary_data.zip › File_S2_GENETICS-2024-307061.docx]

**Tail Standards: Plasmid Construction and Nanopore Sequencing**

All standards were built from pJJR82 to create a sequence that would be “carrier mRNA” for the tails, but any template can be used. XbaI restriction site was incorporated in order to estimate tail size when cut, but sequencing has demonstrated that accuracy is high and this step is not necessary.

*Plasmid Design*

Design the reverse primer with a restriction site at the 5’ end (3’ on the reverse complement) that leaves behind only the poly(A) or (T) tail of the resulting forward strand. The same restriction site is added to the 5’ end of the forward primer, unless already present within the selected region of pJJR82, so that the final cut product is 400 – 500 bp. Avoid portions of pJJR82 with multiple cut sites for that restriction enzyme. The forward primer should have a T_m_ of 51^o^C ± 5^o^C to match M13 Reverse primer for colony PCR screening.

Standards ending in poly(A) on forward strand:

Forward primer: Reverse primer:

5’-[SpeI match pJJR82]-3’ 5’[SpeI polyT tail XbaI match pJJR82]-3’

Example: poly(A)_30_

Forward primer:

ACTAGTAATCATGTCACTTTACCAC

Reverse primer:

ACTAGTTTTTTTTTTTTTTTTTTTTTTTTTTTTTTCTAGACAGATCAGTCATAACCAAG

Amplified product:

ACTAGTaatcatgtcactttaccactatttccgtccagctcaacgttccgtttttggtgatcATTTTTGCTTTCGTCGTAAATCTACACACGCGTCTCTTCCGTGCGAGAGTCCAAGCCAGCAGCCAAATTCGTTGACTGAGTATTCAACGTTTATACGTTGTCGGCAACGAGAAATAGGAAAATGCATCGGGAAATGTTCTTTTTTCGATTTTTTCCAAGGTTTTGACAAATTTTACCACGAATTTTGCTATGTTTTCAATTAAAAAATATGTTATTCAACTGTTTCTATGAGGAAAATAAGGCTTTGCATGTAATTTTCTTATTCAGCATAATTTTTAATTAATTTGAATTTTCTGTCCTAACGTTTATTTTGTTTTCTTGGTTATGACTGATCTGTCTAGAAAAAAAAAAAAAAAAAAAAAAAAAAAAAACTAGT

Standards ending in poly(T) on forward strand:

Forward primer: Reverse primer:

5’-[match pJJR82]-3’ 5’[DraI polyA tail XbaI match pJJR82]-3’

Example: poly(T)_10_

Forward primer:

GAGGTTGATGGATTCAG

Reverse primer:

TTTAAAAAAAAAATCTAGATCCAACTGGTCCTTGTG

Amplified product:

GAGGTTGATGGATTCAGAGAGAAGCTTGATACCGAGATGAATGTGTTCAGAgtgagttaaattatattttgaattttaaataatttttaattttctagCAATCTACCAATGGATTGTGGAAGGACATAGTTGTCATCGGAAGATCTAGCAAGCGTGTCCGTTGTCAATATGAAGAGACCAACGCTACCCCAACTCCACATGCTGATGGATCCCCATCTGCTCCACCAGGTCAACCACCAGCAGTTCCACCAGTCTTCAACCAGCCAAAGACTCCAAATGGAGCCAATGGAAATGGACCAACCTGCAACTGCAATGCTGATAACAAGTGCCCAGCTGGACCATCCGGACCAAAGGGAGTTCCAGGAGTTCCAGGACTCGACGGAGTTCCAGGACTTGACGGTGTTCCAGGAGTTGGAGCTGATGATATCGCTCCACAACGCGAGTCTGTCGGATGCTTCACTTGCCCACAAGGACCAGTTGGATCTAGATTTTTTTTTTAAA

**Plasmid Construction**

**Day 1**

*Plasmid Inserts*

1. Calculate the annealing temperatures of the forward and reverse primers using NEB’s T_m_ Calculator for Q5 High-Fidelity DNA Polymerase, and a primer concentration of 400 nM. Only enter the portion of the primer sequences matching pJJR82.
2. Run the following PCR reaction with three parallel 50 µl reactions:

|  | x1 | x3 |  |  |  |  |  |
| --- | --- | --- | --- | --- | --- | --- | --- |
| **Reagent** | **Volume (µl)** | |  |  | | **PCR** |  |
| 5x Q5 buffer | 10 | 30 |  | Cycles | | Temperature (^o^C) | Time |
| pJJR82, 70 ng/µl (10fmol/µl) | 1 | 3 |  | 1 |  | 98 | 30 sec |
| dNTP (10 mM) | 1 | 3 |  |  |  | 98 | 10 sec |
| Forward primer (10 µM) | 1 | 3 |  | 9 |  | From T_m_ Calculator | 30 sec |
| Reverse primer (10 µM) | 1 | 3 |  |  |  | 72 | 30 sec |
| Q5 polymerase | 0.5 | 1.5 |  | 1 |  | 72 | 2 min |
| H_2_O | 36 | 106.5 |  |  |  |  |  |
| **Final Volume:** | **50** | **150** |  |  |  |  |  |
| *Note: If using a different plasmid template, keep concentration at 10 fmol/µl (might not be 70 ng/µl)* | | |  |  |  |  |  |

1. Check PCR product on a 1% agarose gel. If a band is visible (even faintly), proceed. Otherwise, the annealing temperature may need to be optimized.
2. Load remaining product onto a 1% agarose gel for extraction. Cut the appropriate band from the gel, purify using the Monarch DNA Gel Extraction Kit (NEB, Cat. #T1020), and elute in 9 µl dH_2_O. Check concentration on a NanoDrop using 1 µl of product. Concentrations as low as 5 ng/µl may still work well.

*Note: If the concentration is at least 20 ng/µl, remove 80 ng and dilute in dH_2_O to a total volume of 7.8 µl. The remaining purified product can be stored at 4^o^C and used again if the ligation fails (potentially at a higher concentration). Product cannot be stored after A-tailing.*

*Ligation*

1. Set up the following A-tailing reaction:

| **Reagent** | **Volume (µl)** |
| --- | --- |
| Purified PCR product | 7.8 |
| 10x Taq buffer | 1 |
| dATP (10 mM) | 1 |
| Taq polymerase | 0.2 |
| **Final Volume:** | **10** |

1. Incubate at 72^o^C for 20 minutes. Drop temperature to 4^o^C and proceed immediately to the next step.

*Note: A starting molar ratio of at least 3:1 insert:vector is recommended for ligation. Most inserts for this protocol are ~0.5 kb and the pGEM-T Vector is 3.0 kb. Under those parameters, a 3:1 molar ratio will be 25 ng of insert per 50 ng vector, or 8 ng/µl. The A-tailed product should already meet this requirement. If ligation is unsuccessful, a ratio as high as 8:1 can be tested.*

1. Set up the following ligation reaction:

| **Reagent** | **Volume (µl)** |
| --- | --- |
| A-tailed product | 3 |
| 2x ligation buffer | 5 |
| pGEM-T Vector (50 ng/µl) | 1 |
| T4 DNA ligase | 1 |
| **Final Volume:** | **10** |

1. Incubate overnight (18 – 24 hours) at 4^o^C.

**Day 2**

*Primer Optimization*

*Note: The purpose of this step is to select an optimal annealing temperature for colony PCR using Taq polymerase and can be completed any time before Day 3. Although only the forward primer will be used for colony PCR (paired with M13 reverse primer), M13 is located within pGEM-T and cannot be tested here. However, M13 is already known to anneal well from at least 50^o^ – 55^o^C. Pairing an internal forward primer with an external reverse primer verifies proper insert orientation.*

1. Set up the following PCR reaction, dividing into six identical 25 µl reactions:

|  | x1 | x6 |  |  |  | |  |
| --- | --- | --- | --- | --- | --- | --- | --- |
| **Reagent** | **Volume (µl)** | |  |  | | **PCR** |  |
| 10x Taq Buffer | 2.5 | 15 |  | Cycles | | Temperature (^o^C) | Time |
| pJJR82 (2 ng/µl) | 0.5 | 3 |  | 1 |  | 95 | 2 min |
| dNTP (10 mM) | 0.5 | 3 |  |  |  | 95 | 30 sec |
| Forward primer (10 µM) | 0.5 | 3 |  | 30 |  | 50 – 60* | 30 sec |
| Reverse primer (10 µM) | 0.5 | 3 |  |  |  | 72 | 30 sec |
| Taq polymerase | 0.1 | 0.6 |  | 1 |  | 72 | 2 min |
| H_2_O | 20.4 | 122.4 |  | *test in 2^o^C increments | | | |
| **Final Volume:** | **25** | **150** |  |  |  |  |  |
|  |  |  |  |  |  |  |  |

1. Check PCR reactions on a 1% agarose gel. Choose the optimal temperature to be used for colony PCR (Day 3).

*Transformation*

1. Thaw 50 µl DH5-α or other chemically competent cells on ice. Add ligation product and tap or pipette to mix (do not vortex). Hold on ice for 30 minutes.
2. Heat shock at 42^o^C for 1 minute. Immediately move to ice and hold for 10 minutes.
3. Add 350 µl LB or SOC. Incubate at 37^o^C with agitation for 30 – 90 minutes.
4. Meanwhile, prepare two selection plates by spreading 25 µl Ampicillin (100 mM), 40 µl IPTG (100 mM), and 40 µl X-Gal (20 mg/ml) evenly onto each LB plate. When dry, store one plate at 4^o^C to use the following day.

*Note: IPTG/X-Gal is used to distinguish ligation products with inserts from those with self- ligated ends. Self-ligation results in a functional β-galactosidase gene, which allows the bacteria to break down X-Gal and form a blue pigment. White colonies are more likely to have an insert while blue colonies are not. However, the disruption is a frameshift and therefore blue colonies may still contain the correct insert if the β-galactosidase gene remains in-frame.*

1. Spread transformation product evenly onto the second plate. Incubate overnight (at least 18 – 20 hours) at 37^o^C.

**Day 3**

*Colony Selection*

1. Set up colony PCR. Remove the second LB/Amp^+^/IPTG/X-Gal plate from 4^o^C and draw a numbered grid on the underside. This will be used to sub-culture (“patch”) colonies picked from the transformation plate. Additionally, prepare PCR strip tubes with corresponding numbers, each containing 30 µl of water. Positive results vary; initially testing 20 colonies is recommended.
2. Using a sterile pipette tip set to 25 µl, dab a single white colony from the transformation plate. Next, lightly touch the end of the tip to one grid box of the patch plate. Dip the end of the same tip into the corresponding PCR tube and pipette up and down 20 times. Discard the pipette tip. Close each cap after pipetting to avoid mix-ups.
3. Move the patch plate to 37^o^C for at least 6 hours or until colonies are clearly visible.
4. Lyse the bacteria by heating the PCR tubes to 95^o^C for 5 minutes. Bring temperature down to 4^o^C before proceeding.
5. Run the following PCR reaction for each sample using the same forward primer used to make the Standard:

|  | x1 | x21 |  |  |  | |  |
| --- | --- | --- | --- | --- | --- | --- | --- |
| **Reagent** | **Volume (µl)** | |  |  | | **PCR** |  |
| 10x Taq Buffer | 2.5 | 52.5 |  | Cycles | | Temperature (^o^C) | Time |
| dNTP (10 mM) | 0.5 | 10.5 |  | 1 |  | 95 | 2 min |
| Forward primer (10 µM) | 0.5 | 10.5 |  |  |  | 95 | 30 sec |
| M13 Reverse primer (10 µM) | 0.5 | 10.5 |  | 30 |  | Varies* | 30 sec |
| Taq polymerase | 0.1 | 2.1 |  |  |  | 72 | 40 sec |
| H_2_O | 10.9 | 228.9 |  | 1 |  | 72 | 2 min |
| Lysed template | 10.0 | --- |  | *selected from *Primer Optimization* step | | | |
| **Final Volume:** | **25** | **315** |  |  |  |  |  |
|  |  |  |  |  |  |  |  |

1. Check PCR reactions on a 1% agarose gel. Presence of a band at ~500 – 700 bp indicates an insert with the correct orientation.

*Note: If no positive colonies are identified, re-test using another 10 µl of lysate and the forward and reverse primers used to create the insert. Presence of a band indicates that the ligation was likely successful but in the wrong orientation. Steps 4 – 8 can be repeated with additional colonies from the same transformation plate. If no bands are present, repeat ligation at a higher or lower molar ratio.*

1. When colonies are visible on the patch plate (6+ hours), pick candidates into 3 ml liquid LB/Amp^+^ using a sterile toothpick. Incubate overnight (15 – 18 hours) at 37^o^C on a shaker. The patch plate can be returned to 37^o^C overnight for larger colonies or stored at 4^o^C.

**Day 4**

*Plasmid Isolation and Verification*

1. Centrifuge cultures at max speed (3,200 rpm) for 4 minutes. Pour off liquid.
2. Collect plasmids using the Monarch Plasmid Miniprep Kit (NEB, Cat. #T1010) following manufacturer’s instructions*. Elute in 30 µl dH_2_O. Check concentration on a NanoDrop.

**To increase yield, it is recommended that the supernatant be mixed 1:1 with 95% ethanol following the 2 – 5 minute spin, just before loading onto the column.*

1. Verify the correct size of insert and presence of cut sites by digesting* plasmids either for 1 hour or overnight^†^:

|  | **Volume (µl)** | |
| --- | --- | --- |
| **Reagent** | 1 hour | Overnight |
| Plasmid (200 – 500 ng/µl) | 1 | 1 |
| 10x CutSmart buffer | 1 | 1 |
| DraI or SpeI restriction enzyme | 1 | 0.1 |
| dH_2_O | 7 | 7.9 |
| **Final Volume:** | **10** | **10** |

**Digest with SpeI for standards ending in poly(A) and DraI for standards ending in poly(T)*

^†^*Overnight digestion is recommended in order to save enzyme, but a 1 hour digestion can be used if sending plasmids for sequencing the same day*

1. Prepare candidate plasmids for sequencing:

- 50 ng plasmid in 10 µl dH_2_O for each tube (use strip tubes)
- Two replicate tubes per candidate, each to be sequenced with either M13F or M13R primer
- Label tubes “XX1, XX2, XX3…”

1. Go to the GENEWIZ website and fill out the Sample Submission form using the example below. Keep all parameters the same except DNA Name.


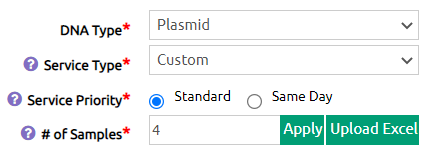


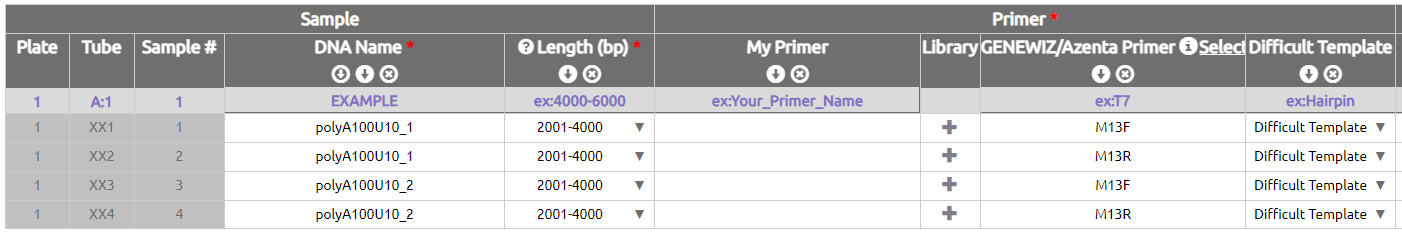


1. When results are available, verify the correct sequence using the BLAST multiple sequence alignment. If any bases do not match, double check the GENEWIZ trace file for both the forward and reverse sequencing. Results are typically available within two days of submission.
2. Pick confirmed colonies from the patch plate onto an LB/Amp^+^ plate. Store at 4^o^C.

**Standard Purification**

**Day 1**

*Plasmid Digestion*

1. Prepare four tubes each with 3 ml of LB/Amp^+^. Pick individual colonies from the streak plate into the tubes. Incubate overnight (15 – 18 hours) at 37^o^C on a shaker.

**Day 2**

1. Centrifuge cultures at max speed (3,200 rpm) for 4 minutes. Pour off liquid.
2. Collect plasmids using the Monarch Plasmid Miniprep Kit (NEB, Cat. #T1010) following manufacturer’s instructions*. Pool samples by spinning all four preps through the same column, one at a time. Elute twice in 15 µl dH_2_O for a total of 30 µl. Check concentration on a NanoDrop.

**To increase yield, it is recommended that the supernatant be mixed 1:1 with 95% ethanol following the 2 – 5 minute spin, just before loading onto the column.*

1. Adjust the plasmid concentration to 500 ng/µl. Set up the following reaction and incubate overnight (15 – 24 hours) at 37^o^C:

|  | **Volume (µl)** | |
| --- | --- | --- |
| **Reagent** | poly(A) tail | poly(T) tail |
| Plasmid (500 ng/µl) | 42.5 | 42.5 |
| 10x CutSmart buffer | 5 | 5 |
| SpeI restriction enzyme | 2.5 | --- |
| DraI restriction enzyme | --- | 2.5 |
| **Final Volume:** | **50** | **50** |

**Day 3**

1. Confirm digestion by diluting 1 µl of product into 9 µl of water and loading 1 µl onto a 1% agarose gel. Include an undigested plasmid control (500 ng/µl), 0.5 µl diluted in 9.5 µl of water and 1 µl loaded onto the gel. Product can be used even if a small amount of plasmid remains undigested.

For poly(T) standards, proceed to Step 7.

1. Inactive SpeI by incubating tube at 80^o^C for 20 minutes. Reduce temperature to 21^o^C before proceeding.
2. Add 2 µl Mung Bean Nuclease, pipetting up and down to mix. Incubate at 30^o^C for 30 minutes.

*Standard Purification*

1. Add 50 µl dH_2_O and 20 µl 6x loading dye to the tube and load onto a 1% agarose gel. Run at 100 V for 45 minutes.
2. Cut the band (~400 – 500 bp) from the gel on a UV box, working quickly to minimize UV exposure that may lead to mutations in the DNA.
3. Purify the DNA from the gel slice using the Monarch DNA Gel Extraction Kit. Elute twice in 25 µl for a total volume of 50 µl.
4. Store purified DNA at -20^o^C. Standard can now be used directly with the Nanopore Ligation Sequencing Kit (SQK-LSK110).

**Library Preparation protocol for Nano3P-Seq**

*The library preparation protocol below is modified from Begik et al. (2023) and the ONT Direct cDNA Sequencing – native barcoding (SQK-DCS109 with EXP-NBD104 and EXP-NBD114) protocols.*

Materials and consumables required:

- Direct cDNA Sequencing Kit (SQK-DCS109)
  - Components used in this protocol:
    - WSB, EB, SQB, LB 🡨 These reagents are not unique to this kit.
  - Components **not** used in this protocol:
    - SQT, SSP (our SSP is the D_DNA/R_RNA hybrid oligo), PR2, VNP (our custom VNP is CompA_DNA)
- 0.5M NaCl (sterile filtered)
- 0.1M Tris-Cl, pH7.5 (sterile filtered)
- RNase Inhibitor, Murine (NEB, M0314)
- Nuclease-free water
- 5x TGIRT reaction buffer (sterile filtered)
  - 2.25 M NaCl
  - 25 mM MgCl_2_
  - 100 mM Tris-HCl, pH 7.5
- 0.1M DTT
- TGIRT™-III Enzyme (InGex or Fisher Scientific)
- 10mM dNTPs
- RNase Cocktail Enzyme Mix (Thermo Fisher)
- AMPure XP beads (Beckman Coulter, A63880)
- Freshly prepared 70% ethanol in nuclease-free water
  - Each sample uses a total of 600 µL 70% ethanol throughout the whole protocol
- dH_2_O
- Blunt/TA Ligase Master Mix (NEB, M0367)
- NEBNext Quick Ligation Module (E6056)
  - Comes with 5x Quick Ligation Reaction Buffer (B60580) and Quick T4 DNA Ligase (E6057)
- Native Barcoding Expansion 1-12 (PCR-free) (ONT, EXP-NBD104)
  - Comes with Adapter Mix II Expansion (EXPAMII001)
- Flow Cell Priming Kit (ONT, EXP-FLP001)
  - Contains Flush Buffer (FB) and Flush Tether (FLT)
- Qubit and Qubit ssDNA Assay Kit (Q10212)

Oligos required:

| **Oligo Name** | **Stock**  **Concentration** | **Sequence** |
| --- | --- | --- |
| D_DNA | 100µM | /5Phos/CTTCCGATCACTTGCCTGTCGCTCTATCTTCN |
| R_RNA | 100µM | rGrArArGrArUrArGrArGrCrGrArCrArGrGrCrArArGrUrGrArUrCrGrGrArArG/3SpC3/ |
| CompA_DNA | 100µM | GAAGATAGAGCGACAGGCAAGTGATCGGAAGA |

CompA_DNA can be ordered from Invitrogen (desalt, 25N synthesis scale).

D_DNA can be ordered from IDT (desalt, 25 nmole).

R_RNA can be ordered from IDT (desalt, 100 nmole, resuspended in nuclease-free water and stored in aliquots at -80°C).

**1. Preannealing of the oligos**

We need pre-anneal R_RNA and D_DNA oligos in order to be able to initiate template switching.

- In a PCR tube, add the following and pipette up/down to mix:

| **Reagent** | **Initial Concentration** | **Volume** | **Final Concentration** |
| --- | --- | --- | --- |
| R_RNA | 100 µM | 1 µL | 10 µM |
| D_DNA | 100 µM | 1 µL | 10 µM |
| Tris-Cl, pH7.5 | 0.1 M | 1 µL | 0.01 M |
| NaCl | 0.5 M | 1 µL | 0.05 M |
| RNase Inhibitor, Murine (NEB) | 40 U/µL | 0.5 µL | 20 U |
| Nuclease-free H_2_O | --- | 5.5 µL | --- |
| TOTAL | --- | 10 µL | --- |

*10 µL of pre-annealed oligos is enough for 4 samples.*

- Heat the mixture for 94°C for 1 mins and ramp down to RT at 0.1°C/s (in a preheated thermocycler) (~15 minutes).

**2. Reverse Transcription**

- In a PCR tube, add the following and pipette up/down to mix:

| **Reagent** | **Initial Concentration** | **Volume** | **Final Concentration** |
| --- | --- | --- | --- |
| 5x reaction buffer | 2.25 M NaCl  25 mM MgCl_2_  100 mM Tris-HCl, pH 7.5 | 4 µL | 450 mM NaCl  5 mM MgCl_2_  20 mM Tris-HCl, pH 7.5 |
| DTT | 0.1 M | 1 µL | 5 mM |
| Pre-annealed oligos | 10 µM | 2 µL | 1 µM |
| rRNA-depleted RNA | varies | Up to 10 µL | 50-100 ng |
| TGIRT | 200 U/µL | 1-2µL | 500 nM – 1,000 nM |
| RNase Inhibitor, Murine | 40 U/µL | 1 µL | 40 U |
| **TOTAL** | --- | **19 µL** | --- |

| **Reagent** | **Volume (µL)** |  |  |  |  |  |
| --- | --- | --- | --- | --- | --- | --- |
| 5x reaction buffer | 4 |  |  |  |  |  |
| DTT (0.1M) | 1 |  |  |  |  |  |
| Pre-annealed oligos | 2 |  |  |  |  |  |
| rRNA-depleted RNA | Up to 10 |  |  |  |  |  |
| TGIRT | 1-2 |  |  |  |  |  |
| RNase Inhibitor, Murine | 1 |  |  |  |  |  |
| **TOTAL** | **19** |  |  |  |  |  |

- Pre-incubate at room temperature for 30 minutes, then add **1 µL of 10 mM dNTPs.**
- Incubate at 60°C for 1 hour (in thermocycler).
- Inactivate the enzyme by incubating at 75°C for 15 mins.
- Move reaction to ice for ≥2 minutes.

**3. RNase treatment**

- Add 1.5 µL RNase Cocktail Enzyme Mix each tube. Mix by pipetting up/down with A P20 set to 10 µL.
- 37°C 10 minutes incubation (in thermocycler).
- Move reaction to ice for ≥2 minutes.

**4. Cleanup using Ampure XP Beads**

- Transfer reaction to 1.5mL tube.
- Resuspend the beads by vortexing for 30 seconds.
- Mix the samples with the 17 µL beads.
  - This is 0.8X, which keeps everything above 150bp (good for getting rid of adapters).
- Mix the beads by pipetting up/down 8 times.
- Incubate 10 minutes at RT. Place it on the magnet for 1 minute. Remove the supernatant.
- Add 70% freshly prepared 200 µL ethanol to the tube while the tube is on the magnet.
- Incubate for 30 seconds at room temperature. Remove the ethanol completely by spinning down (15 seconds, placing back it on magnet, and removing residual ethanol.
- Air-dry the pellet on the magnet for maximum 1 minute, do not let it dry out completely!
- Resuspend the beads in 17 µL dH_2_O while off of the magnet.
- Incubate for 10 minutes at RT.
- Place the beads on magnet for 1 minute.
- Transfer 16 µL supernatant into a new PCR tube.
  - I recommend transferring 16 µL to avoid drawing up beads in the last 1 µL.
- Quantify 1 μL of eluted sample using a Qubit fluorometer with the ssDNA reagents.
  - Goal yield: 70-200 ng

| **Sample** |  |  |  |
| --- | --- | --- | --- |
|  |  |  |  |
|  |  |  |  |
|  |  |  |  |
|  |  |  |  |
|  |  |  |  |

**5. Annealing of Complementary DNA to VNP Oligo**

This step is essential to have a double-stranded DNA oligo with an A overhang, which will initiate the ligation to the adapter

- To the tube containing the cDNA from step 4, add:

| **Reagents** | **Initial Concentration** | **Volume** | **Final Concentration** |
| --- | --- | --- | --- |
| cDNA | --- | 15 µL | --- |
| Tris-Cl, pH 7.5 | 0.1M | 2.25 µL | 0.01 M |
| NaCl | 0.5M | 2.25 µL | 0.05 M |
| CompA_DNA | 100 µM | 1 µL | 4.4 µM |
| dH_2_O | --- | 2 µL | --- |
| Total | --- | 22.5 µL | --- |

- Mix by pipetting up/down.
- Heat the mixture for 90°C for 1 mins and ramp down to RT at 0.1°C/s (in a preheated thermocycler).

Follow the Barcode Ligation and AMII Ligation steps from the *Direct cDNA sequencing - native barcoding (SQK-DCS109with EXP-NBD104 and EXP-NBD114), Version: DCB_9091_v109_revQ_14Aug2019, Last update: 05/07/2022* protocol.

**Direct cDNA sequencing**

Native barcoding (SQK-DCS109 with EXP-NBD104)

Barcode ligation

1. Transfer each sample to a 1.5mL tube. Then, add the reagents in the order given below:
   - 22.5µL cDNA-complement mix
   - 2.5µL Native Barcode
   - 25µL Blunt/TA Ligase Master Mix
2. Pipette up/down 10 minutes to mix.
3. Incubate the reactions for 10 minutes at RT.
4. Resuspend the AMPure XP beads by vortexing.
5. Add 50µL of resuspended AMPure XP beads to the reaction and mix by pipetting.
6. Incubate on a Hula mixer (rotator mixer) for 5 minutes at RT.
7. Spin down the sample (if necessary) and pellet on a magnet for 2 minutes. Keep the tube on the magnet and pipette off the supernatant.
8. Keep the tube on the magnet and wash the beads with 200µL of freshly prepared 70% ethanol without disturbing the pellet. Remove the ethanol using a pipette and discard.
9. Repeat the previous step.
10. Spin down and place the tube back on the magnet. Pipette off any residual 70% ethanol. Briefly allow to dry (2-3 minutes).
11. Remove the tube from the magnetic rack and resuspend the pellet in 26µL nuclease-free water. Incubate for 2 minutes at RT.
12. Pellet the beads on the magnet until the eluate is clear and colorless (2 minutes).
13. Remove and retain 26µL of eluate into a clean 1.5mL Eppendorf DNA LoBind tube.
14. Quantify 1µL of eluted sample on a Qubit fluorometer.
    - Use ssDNA reagents.
    - Goal = >50fol (7.4ng total) or 0.296 ng/µL in 25µL)
15. Pool the barcoded samples at the desired ratio to a final volume of 65μl in a 1.5mL Eppendorf DNA LoBind tube. Aim for as high a concentration as possible which does not exceed 200 fmol total. If the total volume is >65μl, perform a 2.5x AMPure clean up and elute in 65 μl of nuclease free water.

Adapter Ligation

1. Thaw Wash Buffer (WSB), Elution Buffer (EB), and NEBNext Quick Ligation Reaction Buffer (5x) at RT, mix by vortexing, spin down and place on ice. Check the contents of each tube are clear of any precipitate.
2. Spin down the T4 Ligase and the Adapter Mix II (AMII), and place on ice.
3. Check that the contents of each tube are clear of any precipitate and are thoroughly mixed before setting up the reaction.
   - - Check that there is no precipitate present (DTT in the Blunt/TA Master Mix, if used, can sometimes form a precipitate).
     - Spin down briefly before accurately pipetting the contents into the reaction.
4. Taking the pooled and barcoded DNA, perform the adapter ligation as follows, mixing by flicking the tube between each sequential addition:
   - - 65µL 200 fmol pooled barcoded sample
     - 5µL Adapter Mix II (AMII)
     - 20µL NEBNext Quick Ligation Reaction Buffer (5x)
     - 10µL Quick T4 DNA Ligase
5. Mix gently by flicking the tube and spin down.
6. Incubate the reaction for 10 minutes at RT.
7. Resuspend the AMPure XP beads by vortexing.
8. Add 50µL of resuspended AMPure XP beads to the reaction and mix by pipetting.
9. Incubate on a Hula mixer (rotator mixer) for 5 minutes at RT.
10. Place on magnetic rack, allow bead to pellet and pipette off supernatant.
11. Add 140µL of the Wash Buffer (WSB) to the beads. Close the tube lid and resuspend the beads by flicking the tube (spin down for 5 seconds). Return the tube to the magnetic rack, allow bead to pellet and pipette off the supernatant.
12. Repeat the previous step.
13. Spin down and place the tube back on the magnet. Pipette off any residual supernatant. Allow to dry for ~30 seconds, but do not dry the pellet to the point of cracking.
14. Remove the tube from the magnetic rack and resuspend the pellet in 13µL of Elution Buffer (EB).
15. Incubate on a Hula mixer (rotator mixer) for 10 minutes at RT.
16. Pellet the beads on the magnet until the elualte is clear and colorless.
17. Remove and retain 13µL of eluate into a clean 1.5mL Eppendorf DNA LoBind tube.
    - - Quantify 1µL of eluted sample using a Qubit fluorometer and the ssDNA reagents.
      - Goal = 60ng

*The prepared library is used for loading onto the flow cell. Store the library on ice until ready to load.*

Priming and loading the SpotON flow cell

1. Thaw the Sequencing Buffer (SQB), Loading Beads (LB), Flush Tether (FLT) and one tube of Flush Buffer (FB) at RT before mixing the reagents by vortexing, and spin down the SQB, FB and FLT at RT.
2. Prepare the flow cell priming mix: Add 30µL of thawed and mixed Flush Tether (FLT) directly to the tube of thawed and mixed Flush Buffer (FB), and mix by vortexing.
3. Open the MinION Mk1B lid and slide the flow cell under the clip.
4. Slide the priming port cover clockwise to open the priming port.
5. After opening the priming port, check for a small air bubble under the cover. Draw back a small volume to remove any bubbles (a few µL):
   - Set a P1000 pipette to 200µL.
   - Insert the tip into the priming port.
   - Turn the wheel until the dial shows 200-230µL to draw back 20-30µL or until you can see a small volume of buffer entering the pipette tip. *Note: Visually check that there is continuous buffer from the priming port across the sensor array.*
6. Load 800µL of the priming mix into the flow cell via the priming port, avoiding the introduction of air bubbles. Wait for 5 minutes. During this time, prepare the library for loading by following the steps below.
7. In a new tube, prepare the library for loading as follows:
   - 37.5µL Sequencing Buffer (SQB)
   - 25.5µL Loading Beads (LB), mixed immediately before use
     - The Loading Beads (LB) tube contains a suspension of beads. These beads settle very quickly. It is vital that they are mixed immediately before use.
   - 12µL DNA library
8. Complete the flow cell priming:
   - Gently lift the SpotON sample port cover to make the SpotON sample port accessible.
   - Load 200µL of the priming mix into the flow cell via the priming port (not the SpotON sample port), avoiding the introduction of air bubbles.
9. Mix the prepared library gently by pipetting up and down just prior to loading.
10. Add 75µL of sample to the flow cell via the SpotON sample port in a dropwise fashion. Ensure each drop flows into the port before adding the next.
11. Gently replace the SpotON sample port cover, making sure that bung enters the SpotON port, close the priming port, and replace the MinION device lid.
12. Start the sequencing run.
