# Supplementary material for: Poly(U) polymerase activity in Caenorhabditis elegans regulates abundance and tailing of sRNA and mRNA
Source: Genetics. 2024 Jul 28;228(2):iyae120. doi: 10.1093/genetics/iyae120 (PMC11457939; doi:10.1093/genetics/iyae120)
Supplement: iyae120_Supplementary_Data [file iyae120_supplementary_data.zip › Merged_PDF_GENETICS-2024-307061.pdf]

Poly(U) polymerase activity in *Caenorhabditis elegans* regulates abundance and tailing of sRNA and mRNA

Leanne H. Kelley[1*], Ian V. Caldas[1], Matthew T. Sullenberger[1], Kevin E. Yongblah[1], Adnan M. Niazi[2], Anoop Iyer[1], Yini Li[1], Patrick Minty Tran[1], Eivind Valen[2], Yasir H. Ahmed-Braimah[1], and Eleanor M. Maine[1*]

[1] Department of Biology, Syracuse University, 107 College Place, Syracuse, NY 13244 USA
[2] Computational Biology Unit, Department of Informatics, University of Bergen, 5008 Bergen, Norway

*Corresponding author: emmaine@syr.edu ; lhkelley@syr.edu

Current addresses:
M.T. Sullenberger, Center for Computational and Genomic Medicine, Children's Hospital of Philadelphia, Philadelphia, PA USA
Y. Li, Department of Physiology, Brain Science Institute, Johns Hopkins School of Medicine, Baltimore, MD USA
E. Valen, Department of Biosciences, University of Oslo, 0316 Oslo, Norway

Short running head: *C. elegans* poly(U) polymerase targets

Keywords: *C. elegans*, uridylation, 3' tailing, small RNA, poly(U) polymerase

Abstract

Terminal nucleotidyl transferases add nucleotides to the 3' end of RNA to modify their stability and function. In *Caenorhabditis elegans*, the terminal uridyltransferases/poly(U) polymerases PUP-1 (aka CID-1, CDE-1), PUP-2, and PUP-3 affect germline identity, survival, and development. Here, we identify small RNA (sRNA) and mRNA targets of these PUPs and of a fourth predicted poly(U) polymerase, F43E2.1/PUP-4. Using genetic and RNA sequencing approaches, we identify RNA targets of each PUP and the U-tail frequency and length of those targets. At the whole organism level, PUP-1 is responsible for most sRNA U-tailing, and other PUPs contribute to

modifying discrete subsets of sRNAs. Moreover, expression of PUP-2, PUP-3, and especially PUP-4 limit uridylation on some sRNAs. The relationship between uridylation status and sRNA abundance suggests that U-tailing can have a negative or positive effect on abundance depending on context. sRNAs modified by PUP activity primarily target mRNAs that are ubiquitously expressed or most highly expressed in the germline. mRNA data obtained with a Nanopore-based method reveal that addition of U-tails to non-adenylated mRNA is substantially reduced in the absence of PUP-3. Overall, this work identifies PUP RNA targets, defines the effect of uridylation loss on RNA abundance, and reveals the complexity of PUP regulation in *C. elegans* development.

Introduction

Numerous post-transcriptional mechanisms regulate mRNA stability and function, including covalent modifications, such as phosphorylation, methylation, ubiquitination, nucleobase modifications, mRNA 5'-capping, and 3' tailing, as well as RNA interference (RNAi)-related mechanisms. In RNAi, small RNAs (sRNAs) associate with an Argonaute effector protein to form RNA-induced silencing complexes (RISC) and target mRNAs by sequence complementarity to silence, or in some cases, promote their activity (Billi *et al.* 2014). The best studied classes of sRNAs in the context of RNAi-related mechanisms are short interfering (si) RNAs, micro (mi) RNAs, and PIWI (pi) RNAs. While sRNAs function in post-transcriptional regulation of mRNAs, they themselves can also be post-transcriptionally modified, e.g., by addition of 3' non-templated nucleotide tails (De Almeida *et al*. 2018).

3' tailing is a common phenomenon whose function and regulation are well understood in some contexts and poorly understood in others. One class of 3' modification, uridylation, is accomplished by a family of terminal nucleotidyl transferases (TENTs) called poly(U) polymerases or terminal uridyltransferases (PUPs, TUTases) (reviewed by Scheer *et al.* 2016; De Almeida *et al.* 2018; Warkocki *et al.* 2018). PUPs/TUTases are conserved across eukaryotes, including algae, protozoa, yeast, plants, flies, nematodes, and vertebrates (Liudkovska and Dziembowski 2021).

Uridylation is classically described as negatively regulating mRNA stability; the U-tail in these cases is often added 3' to a poly(A) tail and typically contains fewer than 5 nucleotides (Rissland *et al*. 2008; Rissland and Norbury 2009; Schmidt *et al.* 2010; Lim *et al.* 2014; Lackey *et al.* 2016; Morgan *et al.* 2017; Chang *et al.* 2018; Lipińska-Zubrycka *et al.* 2023). In vertebrates, fission yeast, and *Arabidopsis*, uridylation of polyadenylated mRNAs triggers the recruitment of conserved 3'-5' exonucleases, such as Dis3L2, or 5'-3' exonucleases, such as Lsm1-7 (Rissland and Norbury 2009; Scheer *et al.* 2021; Wu *et al.* 2023). Uridylation of histone mRNAs, which lack polyadenylation, also has been described and linked to degradation (Mullen and Marzluff 2008; Lackey *et al.* 2016). In addition, mRNA cleavage products produced by miRNA-mediated RISC activity are reportedly uridylated (Shen and Goodman 2004). Interestingly, uridylation can protect mRNA from 3'-5' degradation in some cases and ensure that 5'-3' degradation occurs instead (Sement *et al.* 2013; Scheer *et al.* 2021).

As is the case for mRNA, 3' uridylation of miRNA and siRNA has also been implicated in promoting turnover (Li *et al.* 2005; Lehrbach *et al.* 2009; van Wolfswinkel *et al.* 2009; Ibrahim *et al.* 2010; Kim *et al.* 2015; see Liudkovska and Dziembowski 2021) and, in at least some cases, U-tailed miRNAs are degraded by Dis3L2 exonuclease (Chang *et al*. 2013; Faehnle *et al.* 2014; Reimao-Pinto *et al.* 2015, 2016; Yang *et al.* 2020). In *C. elegans*, increased abundance of (at least some) CSR-1 class siRNAs has been observed in the absence of PUP-1 (Xu *et al.* 2018; van Wolfswinkel *et al.* 2009). However, U-tailing is not a global mechanism for miRNA decay in *C. elegans* or human cells (Vieux *et al.* 2021; Yang *et al.* 2022). Notably, Yang *et al*. (2022) found that U-tailing correlates with increased abundance of some miRNAs in HEK293T cells, a result they interpret to mean the uridylation positively regulates abundance of those miRNAs. In addition, 3' uridylation can regulate sRNA biogenesis and activity by altering miRNA target specificity (Yang *et al.* 2019, 2022), regulating which arm is selected for miRNA maturation (Kim *et al.* 2020), limiting production of siRNAs from de-adenylated mRNAs (Scheer *et al.* 2021), and preferentially routing siRNAs to specific Argonaute protein(s) (de Albuquerque *et al.* 2015; Xu *et al.* 2018).

PUPs/TUTases are critical for development, particularly in the vertebrate and invertebrate germline during gamete formation and in the early embryo (Thornton *et al.* 2014; Spracklin *et al.* 2017; Morgan *et al.* 2017, 2019; Chang *et al.* 2018; Li and Maine 2018; Li *et al.* 2021). Uridylation

has been shown to promote widespread turnover of mRNAs during specific developmental windows, e.g., during oocyte maturation (Morgan *et al.* 2017; Wu *et al.* 2023), during spermatogenesis (Morgan *et al.* 2019), and in the early embryo to remove maternal mRNA and facilitate the transition to embryonic gene expression (Chang *et al.* 2018; Zhao *et al.* 2022). In plants, uridylation promotes development by limiting inappropriate sRNA production to allow correct expression of an essential photosynthesis regulator (Wang *et al.* 2022).

C. elegans PUP-encoding genes, *pup-1* (aka *cid-1*, *cde-1*), *pup-2*, and *pup-3*, promote germline and early embryonic development (Li and Maine 2018; Li *et al.* 2021). Loss of *pup-1* function reduces U-tailing of miRNAs and siRNAs (van Wolfswinkel *et al.* 2009; Vieux *et al.* 2021) as well as viral transcripts produced upon Orsay virus infection (Le Pen *et al.* 2018). We hypothesize that *pup* developmental defects, which are exacerbated at high culture temperatures, arise due to inappropriate gene expression resulting from reduced U-tailing of sRNA and mRNA. Here, we used Illumina sRNA-seq and Oxford Nanopore Technologies (ONT) direct cDNA sequencing (Nano3P-seq; Begik *et al.* 2023) to identify sRNAs and mRNAs that are U-tailed by one or more PUP. We included PUP-1, PUP-2, PUP-3, and a fourth gene, F43E2.1, with PUP activity in a heterologous system (Preston *et al.* 2019) in our analyses. We show that F43E2.1 protein is expressed in both germline and soma, and expression promotes aspects of germline development. Given its role in U-tailing certain RNAs, as well as the Preston *et al.* (2019) findings, we refer to *F43E2.1* as *pup-4*. PUP-1 is primarily responsible for uridylating siRNAs, miRNAs, and piRNAs, whereas PUP-2, PUP-3, and PUP-4 uridylate distinct subsets of those sRNAs. Moreover, uridylation frequency of certain sRNAs increases in the absence of PUP-2, PUP-3, and especially PUP-4, suggesting these enzymes may interfere with PUP-1 activity. For mRNA, we observe reduced U-tailing of non-adenylated mRNAs in the absence of PUP-3. Our data suggest that uridylation can positively and negatively regulate sRNA and mRNA abundance, depending on context. Overall, this work establishes the global molecular consequences of uridylation loss and sets the stage for future work on uridylation's specific functions in *C. elegans* development.

Materials and Methods

C. elegans strains and culture

Nematodes were cultured using standard methods (Epstein and Shakes 1995). We used the following mutations. LG (linkage group) I: *pup-3(tm5089), omIs10[3xflag::pup-3].* LGII: *pup-4(om140)*, *pup-4(om141)*, *omIs12[pup-4::3xflag].* LGIII: *pup-1(tm1021)*, *pup-2(tm4344)*, *pup-1/-2(om129)*, *omIs7[pup-2::3xflag]*, *omIs8[pup-1::3xmyc]* (Li and Maine 2018), *glp-1(q231ts)*. *glp-1(q231)* was maintained over the balancer chromosome *hT2 [bli-4(e937) let-?(q272) qIs48]*. *pup-1/-2(om129)*, *pup-1(tm1021)*, and *pup-2(tm4344)* were maintained over the balancer chromosome *qC1[dpy-19(e1259ts) glp-1(q339) nIs189[myo-2::gfp]]* (abbreviated *qC1gfp* below). We used co-CRISPR-Cas9 gene editing to isolate *pup-4(om140)* and *pup-4(om141)* deletion alleles and generate *omIs12[pup-4::3xflag]* (Arribere *et al.* 2014; Paix *et al.* 2014) (see Fig. S1). *dpy-10* and *pup-4* genome edits were simultaneously induced; visible *dpy-10* mutants were recovered and screened for a *pup-4* edit via DNA amplification; candidate edits were confirmed by DNA sequencing. The *dpy-10* mutation was either repaired to wildtype using CRISPR or removed by recombination; strains were back-crossed to wildtype at least four times prior to use. Strains used in this study are listed in Table S1.

Small RNA sequencing and bioinformatic analysis

Strains were grown in parallel at 22°C, and F2 generation individuals were harvested. For strains carrying the *qC1gfp* balancer, we passaged F1 *pup* M+Z- individuals and harvested their F2 *pup* M-Z- offspring as 1-day old adults. "-" indicates the absence of the functional gene product; M and Z indicate maternally contributed and zygotically generated gene products, respectively. RNA was isolated from whole animals using the TRIzol (Invitrogen) method. Small RNA libraries were constructed and sequenced at the Biotechnology Resource Center (BRC) Genomics Facility (RRID: SCR_021727) at the Cornell University Institute of Biotechnology. Prior to library construction, low molecular RNA was column purified and treated with RNA 5' polyphosphatase (Epicentre) to convert triphosphate to monophosphate. Libraries were generated using the NEBNext Small Library Prep Kit; 75 bp, single-ended reads were sequenced to a minimum depth of 10M on an Illumina MiSeq. We generated six independent replicates for wildtype samples and three independent replicates for mutant samples.

For bioinformatic analysis, 3' adapters were trimmed, and 15-26 nt long reads with a 3' quality cutoff of 20 were retained (Martin 2011; Andrews 2010) and mapped to the *C. elegans* WS284 reference genome. Small RNAs and their 3' non-templated tails were identified with our smalldisco pipeline (Caldas *et al.* 2023; github.com/ianvcaldas/smalldisco). In brief, using the *C. elegans* WS272 canonical geneset GTF, miRNAs and piRNAs were selected from the ninth column (gene_biotype) and converted into BED files using BEDOPS' *gtf2bed* (RRID:SCR_012865) (Neph *et al.* 2012). Using the `tail` command, 3' tails on miRNAs and piRNAs were identified by implementing both the sense and antisense read parameter (Chou *et al.* 2015). We defined siRNAs by the gene to which they map antisense; the `sirna` mode was used to identify reads that mapped antisense to CDS regions. All siRNA reads mapping to exons of a single gene were grouped together and referred to as a single siRNA species, labeled by the gene ID. This list of siRNAs was used as input to identify 3' tails using the antisense only parameter. See File S1.

U-tail frequency is defined as the number of U-tailed reads summed across replicates divided by the number of total reads summed across replicates, resulting in a proportion of uridylation activity for each sRNA (Fig. S2). sRNAs with tails of any length containing a single nucleotide type were collapsed into one tail type group, i.e., A-tails, C-tails, G-tails, or U-tails of varying lengths. Tail types containing a mix of different nucleotides (e.g., UG) were categorized as "other." The $\log_2$FC of uridylation frequency for mutant versus wildtype comparisons was calculated by taking the $\log_2$ of (uridylation frequency in mutant/uridylation frequency in wildtype). There was little variation in proportion across replicates of any given genotype. Of note, the values used to calculate tailing were raw reads and not CPM because uridylation frequency is quantified as a proportion of the total reads.

To investigate U-tail proportions rather than read counts, conventional differential expression (abundance) workflows were not appropriate. Instead, as additional criteria to select the most meaningful data points, we implemented two strategies: 1) an abundance cutoff filter to select sRNAs with a robust number of counts, and 2) a stringent overlap filter based on uridylation frequencies in mutants *versus* wildtype. For the abundance cutoff, we selected sRNAs with a minimum count of 50 CPM in a worthwhile number of samples using edgeR::filterByExpr() (RRID:SCR_012802) (Robinson *et al.* 2010); this retained 6,439 sRNAs with sufficient counts for

uridylation analysis. The overlap filter determines if the range of values among three wildtype replicates overlaps with the range of values among three mutant replicates run in parallel (Fig. S2). Here, we calculated the frequency of U-tails for each sRNA in each replicate. If the range of replicate values for an sRNA in wildtype did not overlap with those in a mutant, then we considered that uridylation was meaningfully altered and included that sRNA in downstream analyses. The overlap filter was imposed with a custom script (conceptually demonstrated in Fig. S2). 4,776 sRNAs passed the overlap filter and were retained for further analysis; we refer to these sRNAs as high confidence uridylation targets.

For analyses pertaining to sRNA abundance (expression), the overlap filter was not imposed and sRNAs with a minimum count of 10 CPM were included. Libraries were normalized using EDASeq (RRID:SCR_006751) (Risso *et al.* 2011) and RUVSeq (RRID:SCR_006263) (with k=6) (Risso *et al.* 2014), and differential abundance was assessed using edgeR (RRID:SCR_012802) (Robinson *et al.* 2010).

Nano3P-seq, reagents, and data analysis

Animal staging was the same as for sRNA-seq experiments. mRNA 3' tails were sequenced using Nano3P-seq, a Nanopore-based method that is a modification of direct cDNA-seq (Begik *et al.* 2023). To calibrate *tailfindr* to distinguish poly(U) and poly(A)+poly(U) from poly(A) sequences, we generated tail standards containing 3' poly(U), poly(A), and poly(A)+poly(U) sequences of different lengths (Krause *et al.* 2019). See File S2 for tail standard synthesis protocol and File S3 for tail standard sequences.

Total RNA was isolated using the Trizol method; rRNA was depleted using RNase H as described (Duan *et al.* 2020), and samples were subjected to Nano3P-seq. Modifications to the Nano3P-seq protocol are detailed in File S2. Briefly, an RNA-DNA hybrid oligo that can accommodate any 3' nucleotide was used to prime reverse transcription with TGIRT-III (InGex or Lambowitz lab, UT-Austin). The RNA strand was removed with RNase (Invitrogen, AM2286), and the remaining single-stranded cDNA was prepped for sequencing using the Nanopore Direct cDNA (SQK-DCS109) kit. Replicates were barcoded (EXP-NBD104) and pooled. Seven runs were performed, each containing four (or in one case, two) pooled replicates, on a MinION using

R9.4.1 flow cells (File S3). FAST5 files were basecalled using *guppy_basecaller* and sequence quality was assessed with NanoPlot (RRID:SCR_024128) (De Coster *et al.* 2018). To annotate reads, FASTQ files were demultiplexed with *guppy_barcoder* and mapped to the *C. elegans* transcriptome (RefSeq GCF_000002985.6) using Minimap2 (RRID:SCR_018550) (Li 2018). The *tailfindr* pipeline is described on GitHub under the polyu branch (https://github.com/adnaniazi/tailfindr/tree/polyu). The resulting counts tables were used for analysis. The Nano3P-seq run summary is included in File S3.

We selected transcripts with a minimum count of 10 CPM in a worthwhile number of samples using edgeR::filterByExpr() as having adequate representation in our dataset; this process identified 1,337 mRNAs that we carried forward in downstream tailing analyses (RRID:SCR_012802) (Robinson *et al*. 2010). U-tail and A-tail frequencies were calculated with *tailfindr*. The same group of mRNAs were used in differential abundance analyses. Raw counts of those mRNAs were normalized using RUVSeq with k=6 (RRID:SCR_006263) (Risso *et al.* 2014) and analyzed with edgeR to identify differentially abundant mRNAs.

Protein immunoblotting reagents and protocol

pup-1/-2(om129) and *glp-1(q231ts)* mutations were maintained over a balancer chromosome. Protein extracts were generated from synchronized populations of F2 generation (M-Z-) 1-day old (1d) mutants. For experiments eliminating the germline, all *glp-1(q231ts)* and control strains were grown in parallel. F2 L1 larvae of genotypes *pup-4::3xflag*, *pup-4::3xflag; pup-1/-2(om129)*, *pup-4::3xflag;glp-1(q231ts)*, and *pup-4::3xflag;glp-1(q231ts)pup-1/-2(om129)* were shifted from 15°C (permissive temperature for *glp-1(q231ts)*) to 25°C (restrictive temperature for *glp-1(q231ts)*) and harvested as 1d adults. *glp-1(q231ts)* adults were visually inspected to confirm they lacked germ cells prior to preparing protein extracts. For 22°C cultures, *pup-4::3xflag* and *pup-4::3xflag;pup-1/-2(om129)/qC1gfp* balanced heterozygotes were moved from 20°C to 22°C; F1 (M+Z-) *pup-4::3xflag;pup-1/-2(om129)* hermaphrodites were picked to fresh plates, and a synchronized population of F2 (M-Z-) embryos was collected. F2 animals were harvested as 1d adults. To compare abundance of PUP-1, PUP-2, and PUP-3 in *pup-4(+)* versus *pup-4(om141)* backgrounds, strains were grown at 22°C for two generations, and synchronized populations of

F2 (M-Z-) 1d adults were harvested. Gonads were dissected from adult hermaphrodites, and extracts were prepared as described (Guo *et al.* 2015).

Protein immunoblotting was performed as described (Li and Maine 2018). Proteins were resolved on a 10% polyacrylamide gel for PUP-4::3xFLAG analysis and on a 4-15% polyacrylamide gradient gel (Bio-Rad, #4561086) for PUP-1::3xMYC, 3xFLAG::PUP-2, and 3xFLAG::PUP-3 analysis. Antibodies and dilutions used were anti-FLAG (Sigma-Aldrich Cat# F1804, RRID:AB_262044, 1:1,000), anti-MYC (Thermo Fisher Scientific Cat# PA1-981, RRID:AB_325961, 1:1,000), anti-actin (DSHB Cat# jla20, RRID:AB_528068, 1:500 or 1:1,000), and anti-beta-tubulin (DSHB Cat# E7, RRID:AB_528499, 1:1,000). Immunolabeling was visualized using Pierce SuperSignal West Pico (PUP-1, PUP-2, PUP-4) or Femto (PUP-3). Fiji (RRID:SCR_002285) software was used to quantify signal intensity (Schindelin *et al.* 2012). Background signal was subtracted from each PUP signal, which was then normalized to the loading control signal in the same lane. Values in each lane were then normalized to the average PUP value in the appropriate control strain carrying the epitope tag and wildtype alleles of *pup* and *glp-1* genes.

DAPI staining and germline analysis

Intact animals were fixed with -20°C methanol, stained with DAPI, processed, and observed with a Zeiss Axioscope or Leica DM5500 as described (Li and Maine 2018). Nuclear morphology was used to identify mitotic and meiotic nuclei, including developing sperm and oocytes.

Alignment and phylogenetic analysis

The top F43E3.1-related proteins were aligned, and neighbor-joining trees were built using Geneious software (www.geneious.com) (Kearse *et al*. 2012). Conserved domains were identified with programs available at NCBI. Intrinsically disordered regions were predicted using AlphaFold (https://alphafold.ebi.ac.uk/), DisoRDPbind (http://biomine.cs.vcu.edu/servers/DisoRDPbind/), and IUPred2a (https://iupred2a.elte.hu/).

Statistical analysis summary

Statistical analyses and data visualization were performed using R Project for Statistical Computing (RRID:SCR_001905) and Jupyter Notebook (RRID:SCR_018315). To assess significant differences for protein quantifications, we used a Dunnet's test followed by a Tukey multiple comparisons test (Fig. 1A) or a two-sided Student's t-test (Fig. 1B, C, D). At least three biological replicates were obtained. Differences in uridylation frequencies were determined by Kruskal-Wallis test followed by a Dunn's test with Bonferroni correction (Fig. 2). At least three biological replicates were obtained for each genotype in both sRNA-seq and Nano3P-seq experiments. For differential abundance analysis, edgeR was used to identify significant differences with an arbitrary cutoff of fold change greater than 2 and FDR < 0.05 for sRNA datasets and a fold change of greater than 2 and FDR < 0.01 for mRNA datasets. Tissue enrichment was calculated using a tissue specificity index based on cummeRbund's csSpecificity function; chi-square tests and corrected *p*-values are reported. GO analyses were performed using the WormBase Enrichment Tool with a *q*-value threshold of 0.1 and using all non-zero count sRNAs in our dataset as the background genes (Angeles-Albores *et al.* 2016, 2018).

Results

PUP-4 is expressed in germline and somatic tissues

PUP-1, PUP-2, and PUP-3 are detected in the germline at a relatively high level and more lowly detected in somatic tissues (Li and Maine 2018). *F43E2.1* and *pup-1* mRNAs are present at comparable levels in our wildtype gonad transcriptome dataset (RPKM of 45 and 50, respectively; RPKM, reads per kilobase of transcript, per million mapped reads) (Guo *et al.* 2015), hence we expected the F43E2.1 protein (hereafter called PUP-4) to be present in the germline. We easily detected 3xFLAG::PUP-4 and PUP-4::3xFLAG in protein extracts from dissected gonads (below) and not by immunolabeling; therefore, we relied on protein blots to evaluate PUP-4 abundance. To discern germline vs somatic expression, we first evaluated PUP-4::3xFLAG in animals with and without germ cells. We eliminated germ cells using a conditional allele of the Notch-type receptor gene, *glp-1(q231ts)*, whose product is essential for maintaining germline stem cells (Austin and Kimble 1989; Kodoyianni *et al.* 1992; Maine and Kimble 1993) (see Methods). The average PUP-4::3xFLAG abundance in *glp-1(q231ts)* adult hermaphrodites

was reduced to ~21% of *glp-1(+)* control levels (Fig. 1A), consistent with a substantial portion of the PUP-4::3xFLAG protein pool being expressed either in the germline or in the soma upon communication from the germline. To distinguish between these alternatives, we compared PUP-4::3xFLAG expression in intact animals and dissected gonads (comprising primarily germ cells). PUP-4::3xFLAG signal was substantially enriched in dissected gonad samples (Fig. 1B). We conclude that PUP-4 is highly expressed in germ cells compared to somatic tissues.

We previously observed a ~2.5-fold increase in PUP-3 abundance in *pup-1/-2(om129)* double mutants compared to wildtype, suggesting that PUP-1 and PUP-2 together directly or indirectly limit PUP-3 expression (Li and Maine 2018). (Unless otherwise indicated, we hereafter use "*0*" to indicate the null mutations described in Materials and Methods.) To determine whether PUP-1 and PUP-2 influence PUP-4 abundance, we compared PUP-4::3xFLAG level in *pup-1/-2(0)* double mutant and *pup-1/-2(+)* control adults (see Methods for culture scheme). Although the average PUP-4::3xFLAG signal was reduced in *pup-4::3xflag;pup-1/-2(0)* F2 adults at 25°C and 22°C compared to *pup-4::3xflag;pup-1/-2(+)* controls, the difference was not statistically significant at either temperature (Fig. 1A, C). We compared somatic PUP-4::3xFLAG expression in *pup-4::3xflag;pup-1/-2(0)glp-1(q231ts)* and *pup-4::3xflag;pup-1/-2(+)glp-1(q231ts)* adults raised in parallel at 25°C. PUP-4::3xFLAG abundance was comparable in both strains, suggesting the loss of PUP-1/-2 had little effect on PUP-4 level in somatic tissues. Low PUP-4::3xFLAG abundance in *pup-1/-2(0)glp-1(q231ts)* adults (~14% of control levels) (Fig. 1A) is consistent with the germline being required for most of the PUP-4 pool.

We evaluated the impact of PUP-4 loss on expression of other PUP proteins in *pup-4(0)* strains that carried endogenously tagged *pup-1::3xmyc*, *3xflag::pup-2*, or *3xflag::pup-3* (Li and Maine 2018) (see Methods). In synchronized F2 M-Z- adult hermaphrodites, the abundance of PUP-1::3xMYC, 3xFLAG::PUP-2, and 3xFLAG::PUP-3 was statistically similar in *pup-4(0)* mutants vs *pup-4(+)* controls (Fig. 1D), and we conclude that PUP-4 does not substantially influence production of other PUPs.

***pup-4* mutants have mild germline defects**

We analyzed the *pup-4* deletion phenotype using two CRISPR-Cas9 generated deletion alleles, *pup-4(om140)* and *pup-4(om141)* (see Methods; Fig S1). Null alleles of *pup-1*, *pup-2*, and *pup-3* are temperature sensitive (Li and Maine 2018), therefore we evaluated *pup-4* mutants at elevated temperature. >99% of *pup-4(om140)* and *pup-4(om141)* mutants raised at 25°C were fertile over >10 generations (Table S2A). We examined DAPI-stained F2, F3, and F4 adult animals and observed some fertile F3 and F4 animals with impaired germline development in one of the two gonad arms, e.g., 13% of *pup-4(om141)* gonad arms in the F3 generation failed to make embryos despite the presence of sperm and oocytes in the arm or, more rarely, failed to produce oocytes (Table S2A). This *pup-4* knockout germline phenotype is similar to that previously described for *pup-3(tm5089)* (Li and Maine 2018).

We tested for genetic interactions between *pup-4(om141)* (hereafter called *pup-4(0)*) and other *pup* genes by asking (i) if *pup-3(0);pup-4(0)* double mutants have a more severe phenotype than either single mutant, and (ii) whether *pup-4(0)* alters the *pup-1/-2(0)* double and/or *pup-3(0);pup-1/-2(0)* triple mutant phenotype with respect to germline development, fertility, and embryonic viability (see Methods). Nearly all *pup-3(0);pup-4(0)* animals produced gametes in at least one gonad arm and were fertile at 25°C (Table S2). Germline defects in the F3 and F4 generations resembled those in *pup-4(0)*; in addition, the very rare gonad arm failed to make either sperm or oocytes, and fertile animals often contained endomitotic oocytes in the uterus, as previously described for *pup-3(0)* single mutants (Table S2A). Germline defects were slightly more penetrant in the *pup-3(0);pup-4(0)* double mutant than in the *pup-4(0)* single mutant (Table S2B) and similar overall to the *pup-3(0)* single mutant (Li and Maine 2018). Based on these data, PUP-3 and PUP-4 do not appear to be functionally redundant.

We observed little effect of *pup-4(0)* on the *pup-1/-2(0)* and *pup-3(0);pup-1/-2(0)* phenotypes at 25°C (Table S2B). In the F3 and F4 generation, *pup-4(0);pup-1/-2(0)* sterile adults typically lacked germ cells, similar to the *pup-1/-2(0)* double mutant, whereas *pup-3(0);pup-4(0);pup-1/-2(0)* sterile adults often contained germ cells (Table S2B), similar to *pup-3(0);pup-1/-2(0)* (Li and Maine 2018). These results suggest that loss of PUP-4 does not substantially modify the *pup-1/-2(0)* or *pup-3(0);pup-1/-2(0)* phenotypes under our laboratory conditions.

sRNA 3' tailing is altered in *pup* mutants

To identify sRNA targets of the PUP proteins, we obtained sRNA-seq data for synchronized wildtype and *pup-1(0)*, *pup-2(0)*, *pup-3(0)*, *pup-4(0)*, *pup-1/-2(0)*, *pup-3(0);pup-1/-2(0)* and *pup-3(0);pup-4(0);pup-1/-2(0)* mutant strains raised in parallel. We identified siRNAs, miRNAs, and piRNAs and their 3' tail sequences for each genotype (see Materials and Methods). For siRNA analysis, we combined all siRNAs targeting the same gene and, although most siRNAs in our datasets are 22G RNAs as is common in *C. elegans*, we included other low abundance siRNAs (Fig. S3). In wildtype, U-tail frequencies ranged widely within all three sRNA classes, averaging ~14% for siRNAs, ~6% for miRNAs, and ~4% for piRNAs (Fig. S4A). Single nucleotide addition, $(U)_1$, comprised ~64% of siRNA U-tails, 94% of miRNA U-tails, and ~96% of piRNA U-tails; longer tails were common only for siRNAs, e.g., ~20% $(U)_2$ and ~10% $(U)_3$ (Fig. S4B). Uridylation was severely reduced on all sRNA classes in the absence of PUP-1 (Fig. S4). Of the siRNA tails present in *pup-1(0)* mutants, >91% were $(U)_1$, suggesting PUP-1 promotes oligo-uridylation (Fig. S4B). Subtle, yet significant, shifts in miRNA and piRNA U-tail lengths were also observed in *pup-1(0)* mutants (Fig. S4). In contrast to *pup-1*, uridylation was subtly altered on all three sRNAs classes in *pup-2(0)*, *pup-3(0)*, and *pup-4(0)* mutants (Fig. S4). Although there was a net *increase* in U-tailing in *pup-4(0)* mutants compared to wildtype (Fig. S4), PUP-4 does not appear to add a different tail type (Fig. S6). Strikingly, uridylation was not eliminated in any *pup* strain, consistent with a low level of U-tailing by one or more additional TENTs in the absence of the defined PUPs (Fig. S4).

Before further analyzing PUP targets, we implemented a filtering strategy to identify with high confidence the sRNAs modified by PUP activity. For each *pup* mutant strain, we identified sRNAs for which the range of U-tail frequencies among replicates did not overlap the range of U-tail frequencies among wildtype replicates (Fig. S2; see Materials and Methods). The 4,776 sRNAs that pass this conservative test in at least one mutant strain are considered to be high confidence uridylation targets of one or more PUP enzymes (Fig. 2A). Most high confidence targets are siRNAs (Table S3). More than 3,000 high confidence uridylated sRNAs lost U-tails in *pup-1(0)* single mutants; their average U-tail frequency was reduced to ~3% (Fig. 2A). Relatively few sRNAs lost U-tails in *pup-2(0)*, *pup-3(0)*, or *pup-4(0)* single mutants where their average

frequency was more modestly reduced to ~8-11% (Fig. 2A). Notably, U-tailing *increased* for other sets of sRNAs in these three single mutants, especially in *pup-4(0)* where average U-tail frequency for a set of 425 sRNAs increased to 17% (Fig. 2A). Increased U-tailing may reflect a loss of inhibition by the slower acting or, in the case of PUP-4, perhaps inactive enzyme. In the *pup-3(0);pup-4(0);pup-1/-2(0)* quadruple mutant, where U-tailing frequency is higher than in the *pup-3(0);pup-1/-2(0)* triple mutant (Fig. 2A; Fig. S4A), tailing is presumably accomplished by another TENT that can better access sRNAs in the absence of PUP-4.

The phylogenetic relationships among *C. elegans* proteins that most closely share domain homology with PUP-4/F43E2.1 are shown in Fig. 2D. These include PUP-1, PUP-2, PUP-3, MUT-2/RDE-3 (documented to add UG to RNA fragments; Shukla *et al.* 2020; Preston *et al.* 2019), GLD-2 (documented to add A to mRNA; Nousch *et al.* 2017), and GLDR-2 (documented to add A to miRNA; Vieux *et al*., 2021). PUP-4 is the most distantly related protein in the group and likely to be the oldest (Fig. 2D). It may have relatively low uridylation activity because it lacks the PAPa and nucleotide recognition motif (NRM) regions present in PUP-1, PUP-2, and PUP-3 (Fig. 2E). The N-terminal region of PUP-1, known to interact with EGO-1 RNA-directed RNA polymerase (van Wolfswinkel *et al*. 2009), may facilitate recruitment to sRNA targets and is absent from other PUPs.

Shared vs unique uridylation targets

To better understand the relationship among PUP activities, we compared the sRNA uridylation targets in strains carrying single and multiple *pup* gene mutations. A larger number of sRNAs lose U-tails in animals carrying *pup-1(0)* and a second *pup* mutation than in *pup-1(0)* single mutants (Fig. 2A, Fig. S9). For example, >3,800 sRNAs have reduced U-tail frequency in *pup-3(0);pup-4(0);pup-1/-2(0)* quadruple mutants compared to ~3,000 in *pup-1(0)* (Fig. 2A). The largest group of sRNAs (1,924) lose uridylation in the four strains carrying *pup-1(0)*, while other PUP-1 targets are also uridylated by PUP-2 (130; Fig. 2B, C VIII) or PUP-3 (103; Fig. 2B, C IX). Moreover, 181 PUP-1 targets have increased U-tailing in *pup-4(0)* mutants (Fig. 2B, C VI). We note that relatively few sRNAs were solely targeted by PUP-2, PUP-3, or PUP-4. For example, U-tailing was reduced for only 51 sRNAs - not targeted by PUP-1 - in *pup-4(0)* mutants (Fig. S9).

Together, these results reinforce the conclusion that PUP-1 is primarily responsible for sRNA uridylation in the adult hermaphrodite and point to unique roles for PUP-2, PUP-3, and PUP-4.

We considered whether differences in PUP-2, PUP-3, and PUP-4 target specificity might reflect, in part, a preference for the last 3' templated nt on a sRNA as has been shown for *Drosophila* Tailor (Reimao-Pinto *et al*. 2015; Bortolamiol-Becet *et al.*, 2015). We identified the 3' templated nt of wildtype U-tailed sRNAs and parsed the sRNAs by the PUP(s) responsible for their modification (Fig. S7). Overall, the most frequent final templated nt is U (~30%) and least frequent is A (~20%), a pattern that holds for targets of each PUP across most or all of their activity ranges (Fig. S7). Subtle shifts are present for PUP-4 at both ends of the activity range and among sRNAs whose U-tailing is limited by PUP-2 or PUP-3 (Fig. S7). Overall, a 3' nt preference is not a major factor in PUP sRNA target choice.

The relationship between sRNA abundance level and U-tail frequency

Based on the differential abundance (DA) patterns we see upon uridylation loss, U-tailing appears to promote turnover of certain sRNAs (red points) and limit turnover of others (blue points), while not significantly affecting abundance of yet others (black points) (Fig. 3). *pup-1(0)* mutants had the largest number of DA sRNAs of any single mutant strain (515 up, 163 down) (Fig. 3) while *pup-3(0);pup-4(0);pup-1/-2(0)* quadruple mutants had the largest number of DA sRNAs in any strain we evaluated (499 up, 331 down) (Fig. 3). Although the mildly elevated U-tailing in *pup-4* single mutants rarely correlated with DA of those sRNAs, the more substantially increased U-tailing in *pup-3(0);pup-4(0);pup-1/-2(0)* animals tended to correlate with increased abundance (Fig. 3). These results reinforce the hypothesis that 3' uridylation has context-dependent functions with respect to sRNA abundance.

sRNAs associated with specific Argonaute proteins

sRNAs classically regulate gene expression by interacting with and guiding specific Argonaute proteins to RNA targets (Billi *et al.* 2014). Argonaute activity is primarily implicated in limiting gene expression, although CSR-1 Argonaute is associated with licensing gene expression (Billi *et al.* 2014; Almeida *et al.* 2019). Taking advantage of recent systematic identification of Argonaute-

associated sRNAs in *C. elegans*, we parsed sRNAs in our datasets by their known Argonaute associations in young adult hermaphrodites (Seroussi et al. 2023) (Fig. 4). Average uridylation frequency reflects (in part) the relative proportion of siRNAs vs miRNAs/piRNAs that associate with an Argonaute, as expected since siRNAs are U-tailed at a higher frequency than miRNAs and piRNAs. In our wildtype dataset, U-tail frequency is highest for VSRA-1 (19%), CSR-1 (18%), and WAGO-4 (16%) associated sRNAs (mostly siRNAs), with a wide range of values around each average (Fig. 4A; sRNAs present at ≥10 CPM). To identify Argonautes whose activity might be especially altered in the absence of PUP-1, we evaluated differential abundance of high confidence uridylated sRNAs, parsed by Argonaute association, in strains carrying *pup-1(0)* (Fig. 4B, Fig. S10). In terms of numbers of sRNAs affected, reduced U-tailing correlated most strongly with increased abundance of sRNAs associated with VSRA-1, CSR-1, and WAGO-4, all germline expressed Argonautes targeting protein-coding RNAs (Fig. 4B). Although the numbers are smaller, a similar percentage of sRNAs increased in abundance for ALG-3/-4 and WAGO-10 (known to function in spermatogenesis), SAGO-2 (soma-enriched expression), and ERGO-1 (functions in early embryogenesis) (Fig. 4B, Fig. S10). In contrast, reduced U-tailing correlated with decreased abundance of subsets of sRNAs associated with HRDE-1 and WAGO-1 (germline-enriched expression, target non-protein coding RNAs) (Fig. 4B). Overall, our data suggest that uridylation may increase or decrease sRNA abundance depending on the specific Argonaute involved and its functional context.

Mis-regulated sRNAs preferentially target genes enriched for germline and ubiquitous expression

Mis-regulation of sRNAs may contribute to the pleiotropic developmental phenotypes observed in *pup* mutants. We investigated this question by examining the biological functions and tissue expression patterns of the targets of sRNAs shown in Fig. 3. Gene Ontology (GO) analysis identified distinct functional classes among genes targeted by DA high confidence uridylated sRNAs (Fig. 5A). Targets of sRNAs with reduced uridylation and abundance in any *pup* mutant are enriched for nine GO terms; the top two include structural components of ribosomes. In contrast, targets of sRNAs with reduced uridylation and elevated abundance are enriched for

numerous GO categories, including several terms involved with embryonic and post-embryonic development and ribonucleotide and nucleotide biochemistry. We hypothesize that PUP activity functions in regulating genes with important functions in the germline development and, by extension, embryogenesis.

We investigated tissue expression using the Serizay *et al.* (2020) database where genes are classified based on enriched expression in individual tissues and combinations of tissues in late L4 - young adult stage wildtype animals. Targets of sRNAs shown in Fig. 3 tend to have either maximum expression in the germline or ubiquitous expression, a pattern that holds for sRNAs with either reduced or increased U-tailing in *pup* mutants (Fig. 5B). Nearly all other tissue expression categories are significantly under-represented among these sRNAs. We also analyzed tissue expression of genes that are targets of DA sRNAs regardless of uridylation status (see Fig. S8); downregulated sRNAs preferentially target genes with maximal expression in the germline, and upregulated sRNAs preferentially target genes with ubiquitous expression or expression in the "sperm" category (Fig. 5C). Interestingly, the ubiquitous category includes *msp* and some other genes that are highly expressed in sperm. Several other tissue expression categories are under-represented in the DA data. Together, these results indicate that U-tailing likely impacts expression of genes with enriched expression in the germline and broad somatic expression in the adult hermaphrodite.

mRNA U-tailing and *pup* mutants

To identify U-tailed mRNAs and compare 3' tailing among *pup* mutants, we chose Nano3P-seq, an Oxford Nanopore Technologies (ONT)-based method (Begik *et al.* 2023). Although ONT is a lower throughput platform than Illumina (library sizes on the order of 10^5 vs. 10^6), the method offers flexibility in library preparation (see Methods). We constructed libraries using RNA isolated from synchronized populations of wildtype and *pup* mutant adult hermaphrodites of the same age as for sRNA-seq. Non-templated 3' nucleotides were identified using a modified version of *tailfindr* software (Krause *et al.* 2019) that can distinguish strings of non-templated A, U, and A followed by U (designated A+U) residues on mRNA 3' ends (see Methods). This approach

identified strict A-tails on ~72% of mRNAs, strict U-tails on ~3.4% of mRNAs, and A+U tails on ~0.93% of mRNAs (Fig. 6A). The strict U-tails were not limited to histone mRNAs, the major class of mRNAs known to lack poly(A) tails. Importantly, limitations on current ONT technology are that *tailfindr* cannot distinguish stretches of fewer than three Us or intermixed As and Us within a tail. Hence, our analysis almost certainly undercounts 3' tails containing U residues, especially runs of A+U. Nonetheless, to our knowledge, our data demonstrate the first direct report of mRNA U-tailing in *C. elegans.*

Nano3P-seq identified changes in A- and U-tailing in *pup-3(0)* single and *pup-3(0);pup-4(0);pup-1/-2(0)* quadruple mutants compared to wildtype (Fig. 6A). The frequency of strict A-tailed reads increased from ~72% in wildtype to ~78% in both *pup-3(0)* and *pup-3(0);pup-4(0);pup-1/-2(0)* quadruple mutants, and the frequency of strict U-tailed reads decreased from ~3.4% in wildtype to ~1.5% in both mutants. A+U-tailing was similar in wildtype and all *pup* mutants (~1%) except *pup-3(0)* mutants (~0.58%). The increased frequency of strict A-tails in the absence of PUP-3 may reflect loss of U residues from intermixed A/U tails, which are not detected by *tailfindr*, as well as loss of strict U-tails. Overall, these results are consistent with PUP-3 functioning to uridylate non-adenylated mRNAs.

We observed mild effects on mRNA A- and U-tail lengths in *pup* mutants. The average strict A-tail length in our wildtype mRNA dataset was ~51 nt, and the most common A-tail lengths were 30 and 33 nt (Fig. 6B). Strict A-tails were shorter than or the same length as wildtype in all *pup* mutant strains (averaging 43-51 nt), and the largest effect on tail length was observed in the quadruple *pup* mutant where average tail length was 43 nt. The average strict U-tail length in the wildtype mRNA dataset was ~5 nt, and the most common length was 3 nt - the shortest U-tail that *tailfindr* reliably detects (Fig. 6C). Average strict U-tail length did not differ significantly between wildtype and any *pup* mutant. Uncommon, long U-tails identified in wildtype were notably less frequent in all *pup* mutants and particularly in *pup-3(0)* and *pup-4(0)* single mutants and the *pup-3(0);pup-4(0);pup-1/-2(0)* quadruple mutant. Overall, loss of PUP-3 is associated with subtle changes in tail length.

Differential mRNA abundance

Differentially expressed mRNAs are difficult to identify reliably with ONT data because the read depth is low. To verify that our dataset had sufficient depth coverage, we compared our Nano3P-seq data with the top one hundred highly expressed genes in *C. elegans*, based on Illumina RNA-seq data from five tissues (Serizay *et al.* 2020). These 100 genes were all represented in our wildtype replicates, suggesting we had adequate coverage for downstream analysis (see File S3). Using conventional edgeR analysis and a cutoff of 2-fold difference between *pup* mutant and wildtype, we identified 592 mRNAs as differentially abundant in at least one *pup* mutant strain (Fig. 7A) (see Methods). Most differentially abundant mRNAs were observed in *pup-3(0);pup-4(0);pup-1/-2(0)* quadruple mutants (166 down, 90 up) and/or *pup-3(0)* single mutants (109 down, 66 up) (Fig. 7A,B). We hypothesize that differential mRNA abundance in *pup* mutants reflects not only any direct impact of reduced U-tailing, but also changes in abundance and uridylation of sRNAs whose activity impacts mRNA abundance. Moreover, since mRNAs are templates for siRNA production, it is likely that changes in mRNA abundance contribute to changes in sRNA levels. Consistent with this relationship, changes in sRNA abundance are more extensive in the *pup-3(0);pup-4(0);pup-1/-2(0)* quadruple mutant than in the *pup-3(0)* single mutant (Fig. 3).

Among differentially abundant mRNAs in *pup-3(0)* and *pup-3(0);pup-4(0);pup-1/-2(0)* mutants, the average strict U-tailing frequency is reduced for both down- and upregulated transcripts. For example, in *pup-3(0)* mutants the average U-tailing drops from ~9% to ~4% among downregulated mRNAs and from ~2% to ~0.4% among upregulated mRNAs (Fig. 7C). A similar pattern holds in *pup-3(0);pup-4(0);pup-1/-2(0)* mutants (Fig. 7C). Interestingly, average U-tailing among these DA transcripts is distinct from the average U-tailing of mRNAs overall, i.e., downregulated mRNAs are uridylated at higher frequency than average in wildtype, and upregulated mRNAs are uridylated at a lower frequency than average in wildtype (Fig. 7C). Considering that U-tail loss correlates with reduced, increased, or unchanged mRNA abundance, PUP-3 activity appears to positively or negatively regulate subsets of mRNA targets depending on context.

Among the 592 DA mRNAs captured by Nano3P-seq, ~10% (61) correspond to DA sRNAs reported here for the same mutant to wildtype comparison. For ~4% (23) of these cases, the

sRNA and mRNA change in the same direction (both up or both down). In addition, ~24% (144) of the DA mRNAs correspond to high confidence uridylated sRNAs in the same mutant to wildtype comparison. These data are consistent with changes in sRNA abundance and sRNA uridylation in *pup* mutants contributing to changes in mRNA abundance. When mRNA and sRNA abundance changes in parallel, this may reflect altered mRNA template availability contributing to altered sRNA production.

Discussion

While RNA uridylation has recently been established as critical for germline and embryonic development in *C. elegans* and other species, much less is known about the specific RNAs that are marked by uridylation and how their abundance may be altered by that modification. Here, we provide a resource for uncovering the importance of U-tailing in regulating *C. elegans* gene expression. Our data indicate that the four known *C. elegans* poly(U) polymerases promote tailing of sRNAs in the adult hermaphrodite; siRNAs are the primary targets, and PUP-1 is primarily responsible for uridylation activity. PUP-2, PUP-3, and PUP-4 have fewer targets in these animals.

Several observations suggest a complex relationship among PUP enzymes *in vivo*. The increased tailing of certain sRNAs in *pup-2(0)*, *pup-3(0)*, and especially *pup-4(0)* mutants suggests that these three enzymes may limit U-tailing - perhaps by PUP-1 - in at least some tissues. This may occur, for example, if they have lower activity than PUP-1 and delay its ability to access target RNAs. Another intriguing observation is that U-tailing is not completely abolished in the *pup-3(0);pup-4(0);pup-1/-2(0)* mutant, and therefore another TENT presumably has 3' uridylation activity in the absence of the PUPs. Studies in *S. pombe* and HEK293T cells similarly have observed residual uridylation in the absence of poly(U) polymerase activity, suggesting the work of another non-PUP/TUTase in those systems (Chung *et al.* 2019; Yang *et al.* 2022). Although *in vitro* evidence suggests many *C. elegans* TENTs preferentially add only A or U, Preston *et al.* (2019) observed a low level of non-preferential tailing by some TENTS, as well as TENTs that commonly add more than one nucleotide type. Another consideration is that Preston

et al. (2019) assayed U-tailing activity in a situation where a single TENT was expressed, and therefore any interaction or interference among different TENTs would not have been observed.

Developmental implications of PUP activity

Uridylation is implicated as regulating RNA stability by signaling turnover of mRNA and some sRNAs (Rissland and Norbury 2009; Lim *et al.* 2014; Kim *et al.* 2015; Morgan *et al.* 2017, 2019; Chang *et al.* 2018; Heo *et al.* 2008; van Wolfswinkel *et al.* 2009; Ibrahim *et al.* 2010; Ren *et al.* 2012; Yang *et al.* 2020, 2022), restabilizing mRNAs (e.g., in *Arabidopsis*) (reviewed by Łabno *et al.* 2016), and allowing mRNAs to remain dormant (e.g., in starfish oocytes and early embryos) (Ochi and Chiba 2016). Our data suggest that sRNA U-tailing in *C. elegans* promotes stability of some sRNAs and limits stability of others. Positively regulated sRNAs are predicted to target mRNAs encoding structural components of ribosomes or proteins otherwise associated with peptide biosynthesis. We find that sRNAs targeting ribosomal protein genes are also uridylated, and these sRNAs become less abundant in the absence of U-tailing. Previous work showed that *C. elegans* ribosomal siRNAs (risiRNAs), rRNAs, and sense rRNA fragments can be uridylated by PUP-1 and/or PUP-2 (Zhou *et al.* 2017; Wahba *et al.* 2021; Wang *et al.* 2020). Together, these data suggest uridylation functions in regulating the handoff from maternal to embryonic ribosomes. Negatively regulated sRNAs, in contrast, are predicted to target genes with a wider range of products including many linked to embryonic and post-embryonic development. Since our sRNA samples were obtained from gravid adult hermaphrodites, we expect them to include oocyte sRNAs that direct gene expression in the early embryo. Uridylation that modulates stability of these sRNAs may promote the transition to embryonic gene expression.

One goal of this work was to gain insight into the *pup* mutant phenotype (Li and Maine 2018; Li *et al.* 2021). The profound effects on sRNA abundance in strains carrying *pup-1(0)* are likely to alter developmental gene expression, and subtle phenotypic differences in strains carrying mutations in one or more additional *pup* genes may reflect differences in sRNA U-tailing and abundance among these strains. In addition, direct effects on mRNA abundance are likely to feedback on sRNA production from mRNA templates. sRNA comparison with Illumina mRNA-seq data show ~66% of overlapping DA sRNAs and mRNA change abundance in the same direction

(both increase or both decrease), consistent with feedback (LH Kelley and EM Maine, unpublished data). Relevant for understanding the complex PUP phenotypes, GO and tissue specificity analyses identified targets of mis-regulated sRNAs as genes with germline-enriched or ubiquitous expression whose products function in developmental processes and ribosome biogenesis. Future analysis of PUP targets at other developmental stages and in somatic tissues, e.g., individual neurons, may identify additional targets. *pup* mRNAs are expressed in multiple somatic cells and tissues, and numerous tissues express *pup-2*, *pup-3*, and/or *pup-4* but not *pup-1* mRNA (Hammarlund *et al*. 2018). The high proportion of germline sRNAs in our adult datasets may obscure important PUP-2, PUP-3, and PUP-4 functions in somatic cells and tissues.

Uridylation tends to correlate negatively with abundance of many sRNAs associated with CSR-1, VRSA-1, WAGO-4, ALG–3/-4 and SAG0-2 - all of which have relatively high U-tailing frequency. In contrast, uridylation tends to correlate positively with abundance of many sRNAs associated with HRDE-1 and WAGO-1 - both of which have relatively lower tailing frequency. These data suggest that uridylation may have different consequences for sRNA stability depending on the associated Argonaute proteins. Previous reports have implicated uridylation as modulating the interaction of shared sRNAs with competing Argonautes CSR-1 and WAGO-4 (Xu *et al.* 2018, de Albuquerque *et al.* 2015; Phillips *et al.* 2015). Our data are consistent with the model that uridylation can stabilize or destabilize sRNAs depending on their interacting Argonaute regardless of competition.

mRNA uridylation in *C. elegans*

We identified a role for PUP-3 in adding 3' uridine to non-polyadenylated mRNAs in the adult hermaphrodite. Although our mRNA analysis was limited by technical issues, the data are consistent with PUP-3 having a distinct role in mRNA U-tailing not shared by other PUPs. The frequency of strict A-tails increased, and frequency of strict U-tails decreased in the absence of PUP-3. Tailing was not further altered in the *pup-3(0);pup-4(0);pup-1/-2(0)* mutant, consistent with single mutant data indicating that PUP-1, PUP-2, and PUP-4 are not individually essential for uridylating non-adenylated mRNAs. Nonetheless, changes in mRNA abundance are more pronounced in *pup-3(0);pup-4(0);pup-1/-2(0)* mutants than *pup-3(0)* mutants, perhaps due to

major changes in the sRNA population in *pup-3(0);pup-4(0);pup-1/-2(0)* compared to *pup-3(0)* animals and/or reduced mRNA uridylation in the absence of PUP-1, PUP-2, and/or PUP-4 that we cannot detect with current *tailfindr* software. We look forward to future technical refinements that will allow better identification of mixed poly(A)+poly(U) tails and the PUP protein(s) responsible for their formation.

Overall, our data reveal changes in uridylation and composition of the *C. elegans* small RNAome and transcriptome in the adult hermaphrodite upon loss of individual and multiple poly(U) polymerases. Identification of PUP RNA targets and relative contributions of different PUPs to RNA modification provides a resource for future work investigating PUP activity in individual cells and tissues.

Data availability

sRNA-seq and Nano3P-seq datasets are deposited in NCBI Gene Expression Omnibus (GEO) as GSE271566 and GSE271568.

Acknowledgements

We are grateful to Sarah Hall for discussion and comments on the manuscript, Alan Lambowitz and members of his research group (University of Texas at Austin) for a generous gift of TGIRT-III enzyme, members of the Hall and Maine laboratories for discussion, and anonymous reviewers for insightful comments on the manuscript. We received invaluable support from Syracuse University computing resources: The Syracuse University HTC Campus Grid, supported by NSF grant ACI-1341006. Tien Thi Pham Huynh and Caleb Porter contributed to our early *pup-4* phenotypic analysis. Some mutations used in this study were obtained from the *Caenorhabditis* Genetics Center, which is funded by the National Institutes of Health Office of Research Infrastructure Programs, and from the National BioResource Project under the direction of Shohei Mitani.

Funding

This work was supported by the National Institutes of Health, grants R03HD0091645 and R15GM139096 to EMM and R35GM147454 to YHAB, and the Research Council of Norway, project #314216 to EDV.

Literature cited

Almeida MV, Andrade-Navarro MA, Ketting RF. 2019. Function and evolution of nematode RNAi pathways. Noncoding RNA. 5:8. doi: 10.3390/ncrna5010008.

Andrews S. 2010. FastQC: a quality control tool for high throughput sequence data. Available online at: http://www.bioinformatics.babraham.ac.uk/projects/fastqc.

Angeles-Albores D, Lee RYN, Chan J, Sternberg PW. 2018. Two new functions in the WormBase Enrichment Suite. microPublication Biol. doi:10.17912/W25Q2N.

Angeles-Albores D, N. Lee RY, Chan J, Sternberg PW. 2016. Tissue enrichment analysis for *C. elegans* genomics. BMC Bioinformatics. 17:366. doi:10.1186/s12859-016-1229-9.

Arribere JA, Bell RT, Fu BXH, Artiles KL, Hartman PS, Fire AZ. 2014. Efficient marker-free recovery of custom genetic modifications with CRISPR/Cas9 in *Caenorhabditis elegans*. Genetics. 198:837–846. doi:10.1534/genetics.114.169730.

Austin J, Kimble J. 1989. Transcript analysis of *glp-1* and *lin-12*, homologous genes required for cell interactions during development of *C. elegans*. Cell. 58:565–571. doi:10.1016/0092-8674(89)90437-6.

Begik O, Diensthuber G, Liu H, Delgado-Tejedor A, Kontur C, Niazi AM, Valen E, Giraldez AJ, Beaudoin J-D, Mattick JS, Novoa EM. 2023. Nano3P-seq: transcriptome-wide analysis of gene expression and tail dynamics using end-capture nanopore cDNA sequencing. Nat Methods. 20:75–85. doi:10.1038/s41592-022-01714-w.

Billi AC, Fischer SEJ, Kim JK. 2014. Endogenous RNAi pathways in *C. elegans*. In *WormBook*, ed. The *C. elegans* Research Community. doi:10.1895/wormbook.1.170.1.

Bortolamiol-Becet D, Hu F, Jee D, Wen J, Okamura K, Lin C-J, Ameres SL, Lai EC. 2015. Selective suppression of the splicing-mediated microRNA pathway by the terminal uridyltransferase Tailor. Mol Cell. 59:217–228. doi:10.1016/j.molcel.2015.05.034.

Caldas IV, Kelley LH, Ahmed-Braimah YH, Maine EM. 2023. smalldisco, a pipeline for siRNA discovery and 3′ tail identification. G3. 13:jkad092. doi:10.1093/g3journal/jkad092

Chang H, Lim J, Ha M, Kim VN. 2014. TAIL-seq: genome-wide determination of poly(A) tail length and 3′ end modifications. Mol Cell. **53**:1044–1052. doi:10.1016/j.molcel.2014.02.007.

Chang H, Yeo J, Kim J, Kim H, Lim J, Lee M, Kim HH, Ohk J, Jeon H-Y, Lee H, Jung H, Kim K-W, Kim VN. 2018. Terminal uridylyltransferases execute programmed clearance of maternal transcriptome in vertebrate embryos. Mol Cell. 70:72-82.e7. doi:10.1016/j.molcel.2018.03.004.

Chang H-M, Triboulet R, Thornton JE, Gregory RI. 2013. A role for the Perlman syndrome exonuclease Dis3l2 in the Lin28–let-7 pathway. Nature. 497:244–248. doi:10.1038/nature12119.

Chou M-T, Han BW, Hsiao C-P, Zamore PD, Weng Z, Hung J-H. 2015. Tailor: a computational framework for detecting non-templated tailing of small silencing RNAs. Nucleic Acids Res. 43:e109. doi:10.1093/nar/gkv537.

Chung CZ, Jaramillo JE, Ellis MJ, Bour DYN, Seidl LE, Jo DHS, Turk MA, Mann MR, Bi Y, Haniford DB, Duennwald ML, Heinemann IU. 2019. RNA surveillance by uridylation-dependent RNA decay in *Schizosaccharomyces pombe*. Nucleic Acids Res. 47:3045–3057. doi:10.1093/nar/gkz043

de Albuquerque BFM, Placentino M, Ketting RF. 2015. Maternal piRNAs Are essential for germline development following de novo establishment of endo-siRNAs in *Caenorhabditis elegans*. Dev Cell. 34:448–456. doi:10.1016/j.devcel.2015.07.010

De Almeida C, Scheer H, Zuber H, Gagliardi D. 2018. RNA uridylation: a key posttranscriptional modification shaping the coding and noncoding transcriptome. WIREs RNA**. 9**:e1440. doi:10.1002/wrna.1440.

De Coster W, D'Hert S, Schultz DT, Cruts M, Van Broeckhoven C. 2018. NanoPack: visualizing and processing long-read sequencing data. Bioinformatics. 34:2666–2669. doi:10.1093/bioinformatics/bty149.

Duan Y, Sun Y, Ambros V. 2020. RNA-seq with RNase H-based ribosomal RNA depletion specifically designed for *C. elegans*. microPub Biol. doi:10.17912/micropub.biology.000312.

Epstein HF, Shakes DC. 1995. *Caenorhabditis elegans: Modern Biological Analysis of an Organism*, Vol. 48. Academic Press.

Faehnle CR, Walleshauser J, Joshua-Tor L. 2014. Mechanism of Dis3l2 substrate recognition in the Lin28–let-7 pathway. Nature. 514:252–256. doi:10.1038/nature13553.

Guo Y, Yang B, Li Y, Xu X, Maine EM. 2015. Enrichment of H3K9me2 on unsynapsed chromatin in *Caenorhabditis elegans* does not target *de novo* sites. G3. 5:1865–1878. doi:10.1534/g3.115.019828.

Heo I, Joo C, Cho J, Ha M, Han J, Kim VN. 2008. Lin28 mediates the terminal uridylation of *let-7* precursor microRNA. Mol Cell. 32:276–284. doi:10.1016/j.molcel.2008.09.014.

Ibrahim F, Rymarquis LA, Kim E-J, Becker J, Balassa E, Green PJ, Cerutti H. 2010. Uridylation of mature miRNAs and siRNAs by the MUT68 nucleotidyltransferase promotes their degradation in *Chlamydomonas*. Proc Nat Acad Sci USA. 107:3906–3911. doi:10.1073/pnas.0912632107.

Kearse M, Moir R, Wilson A, Stones-Havas S, Cheung M, Sturrock S, Buxton S, Cooper A, Markowitz S, Duran C, Thierer T, Ashton B, Meintjes P, Drummond A. 2012. Geneious Basic: An integrated and extendable desktop software platform for the organization and analysis of sequence data. Bioinformatics. 28:1647–1649. doi:10.1093/bioinformatics/bts199.

Kim B, Ha M, Loeff L, Chang H, Simanshu DK, Li S, Fareh M, Patel DJ, Joo C, Kim VN. 2015. TUT7 controls the fate of precursor microRNAs by using three different uridylation mechanisms. EMBO J. 34:1801–1815. doi:10.15252/embj.201590931.

Kim D, Paggi JM, Park C, Bennett C, Salzberg SL. 2019. Graph-based genome alignment and genotyping with HISAT2 and HISAT-genotype. Nat Biotech. 37:907–915. doi:10.1038/s41587-019-0201-4.

Kim H, Kim J, Yu S, Lee Y-Y, Park J, Choi RJ, Yoon S-J, Kang S-G, Kim VN. 2020. A mechanism for microRNA arm switching regulated by uridylation. Mol Cell. 78:1224-1236.e5. doi:10.1016/j.molcel.2020.04.030.

Kodoyianni V, Maine EM, Kimble J. 1992. Molecular basis of loss-of-function mutations in the *glp-1* gene of *Caenorhabditis elegans*. Mol Biol Cell. 3:1199–1213. doi:10.1091/mbc.3.11.1199.

Krause M, Niazi AM, Labun K, Cleuren YNT, Müller FS, Valen E. 2019. *tailfindr*: alignment-free poly(A) length measurement for Oxford Nanopore RNA and DNA sequencing. RNA. 25:1229–1241. doi:10.1261/rna.071332.119.

Łabno A, Tomecki R, Dziembowski A. 2016. Cytoplasmic RNA decay pathways - enzymes and mechanisms. Biochim Biophys Acta. 1863:3125–3147. doi:10.1016/j.bbamcr.2016.09.023.

Lackey PE, Welch JD, Marzluff WF. 2016. TUT7 catalyzes the uridylation of the 3′ end for rapid degradation of histone mRNA. RNA. doi:10.1261/rna.058107.116.

Le Pen J, Jiang H, Di Domenico T, Kneuss E, Kosałka J, Leung C, Morgan M, Much C, Rudolph KLM, Enright AJ, O'Carroll D, Wang D, Miska EA. 2018. Terminal uridylyltransferases target RNA viruses as part of the innate immune system. Nat Struct Mol Biol. 25:778–786. doi:10.1038/s41594-018-0106-9.

Lehrbach NJ, Armisen J, Lightfoot HL, Murfitt KJ, Bugaut A, Balasubramanian S, Miska EA. 2009. LIN-28 and the poly(U) polymerase PUP-2 regulate let-7 microRNA processing in *Caenorhabditis elegans*. Nat Struct Mol Biol. 16:1016–1020. doi:10.1038/nsmb.1675.

Lex A, Gehlenborg N, Strobelt H, Vuillemot R, Pfister H. 2014. UpSet: visualization of intersecting sets. IEEE Transact Visualization Computer Graphics. 20:1983–1992. doi:10.1109/TVCG.2014.2346248.

Li H. 2018. Minimap2: pairwise alignment for nucleotide sequences. Bioinformatics. 34:3094–3100. doi:10.1093/bioinformatics/bty191.

Li J, Yang Z, Yu B, Liu J, Chen X. 2005. Methylation Protects miRNAs and siRNAs from a 3′-End Uridylation Activity in *Arabidopsis*. Curr Biol. 15:1501–1507. doi:10.1016/j.cub.2005.07.029.

Li Y, Maine EM. 2018. The balance of poly(U) polymerase activity ensures germline identity, survival and development in *Caenorhabditis elegans*. Development. 145:dev165944. doi:10.1242/dev.165944.

Li Y, Snyder M, Maine EM. 2021. Meiotic H3K9me2 distribution is influenced by the ALG-3 and ALG-4 pathway and by poly(U) polymerase activity. microPub Biol. doi:10.17912/micropub.biology.000455.

Lim J, Ha M, Chang H, Kwon SC, Simanshu DK, Patel DJ, Kim VN. 2014. Uridylation by TUT4 and TUT7 marks mRNA for degradation. Cell. 159:1365–1376. doi:10.1016/j.cell.2014.10.055.

Lipińska-Zubrycka L, Grochowski M, Bähler J, Małecki M. 2023. Pervasive mRNA uridylation in fission yeast is catalysed by both Cid1 and Cid16 terminal uridyltransferases. PLOS ONE. 18:e0285576. doi:10.1371/journal.pone.0285576.

Liudkovska V, Dziembowski A. 2021. Functions and mechanisms of RNA tailing by metazoan terminal nucleotidyltransferases. WIREs RNA. 12:e1622. doi:10.1002/wrna.1622.

Maine EM, Kimble J. 1993. Suppressors of *glp-1*, a gene required for cell communication during development in *Caenorhabditis elegans*, define a set of interacting genes. Genetics. 135:1011–1022. doi:10.1093/genetics/135.4.1011.

Martin M. 2011. Cutadapt removes adapter sequences from high-throughput sequencing reads. EMBnet.journal. 17:10–12. doi:10.14806/ej.17.1.200.

Morgan M, Kabayama Y, Much C, Ivanova I, Di Giacomo M, Auchynnikava T, Monahan JM, Vitsios DM, Vasiliauskaitė L, Comazzetto S, Rappsilber J, Allshire RC, Porse BT, Enright AJ, O'Carroll D. 2019. A programmed wave of uridylation-primed mRNA degradation is essential for meiotic progression and mammalian spermatogenesis. Cell Res. 29:221–232. doi:10.1038/s41422-018-0128-1.

Morgan M, Much C, DiGiacomo M, Azzi C, Ivanova I, Vitsios DM, Pistolic J, Collier P, Moreira P, Benes V, Enright AJ, O'Carroll D. 2017. mRNA 3′ uridylation and poly(A) tail length sculpt the mammalian maternal transcriptome. Nature. 548:347–351. doi:10.1038/nature23318.

Mullen TE, Marzluff WF. 2008. Degradation of histone mRNA requires oligouridylation followed by decapping and simultaneous degradation of the mRNa both 5' to 3' and 3' to 5'. Genes Dev. 22:50-65.

Neph S, Kuehn MS, Reynolds AP, Haugen E, Thurman RE, Johnson AK, Rynes E, Maurano MT, Vierstra J, Thomas S, Sandstrom R, Humbert R, Stamatoyannopoulos JA. 2012. BEDOPS: high-performance genomic feature operations. Bioinformatics. 28:1919–1920. doi:10.1093/bioinformatics/bts277.

Nousch M, Minasaki R, Eckmann CR. 2017. Polyadenylation is the key aspect of GLD-2 function in *C. elegans*. RNA. 23:1180–1187. doi:10.1261/rna.061473.117.

Ochi H, Chiba K. 2016. Hormonal stimulation of starfish oocytes induces partial degradation of the 3′ termini of cyclin B mRNAs with oligo(U) tails, followed by poly(A) elongation. RNA. 22:822–829. doi:10.1261/rna.054882.115.

Paix A, Wang Y, Smith HE, Lee C-YS, Calidas D, Lu T, Smith J, Schmidt H, Krause MW, Seydoux G. 2014. Scalable and versatile genome editing using linear DNAs with microhomology to Cas9 Sites in *Caenorhabditis elegans*. Genetics. 198:1347–1356. doi:10.1534/genetics.114.170423.

Phillips CM, Brown KC, Montgomery BE, Ruvkun G, Montgomery TA. 2015. piRNAs and piRNA-dependent siRNAs protect conserved and essential *C. elegans* genes from misrouting into the RNAi pathway. Dev Cell. 34:457–465. doi:10.1016/j.devcel.2015.07.009.

Preston MA, Porter DF, Chen F, Buter N, Lapointe CP, Keles S, Kimble J, Wickens M. 2019. Unbiased screen of RNA tailing activities reveals a poly(UG) polymerase. Nat Methods. 16:437–445. doi:10.1038/s41592-019-0370-6.

Reimão-Pinto MM, Ignatova V, Burkard TR, Hung J-H, Manzenreither RA, Sowemimo I, Herzog VA, Reichholf B, Fariña-Lopez S, Ameres SL. 2015. Uridylation of RNA hairpins by Tailor confines the emergence of microRNAs in *Drosophila*. Mol Cell. 59:203–216. doi:10.1016/j.molcel.2015.05.033.

Reimão-Pinto MM, Manzenreither RA, Burkard TR, Sledz P, Jinek M, Mechtler K, Ameres SL. 2016. Molecular basis for cytoplasmic RNA surveillance by uridylation-triggered decay in Drosophila. EMBO J. 35:2417–2434. doi:10.15252/embj.201695164.

Ren G, Chen X, Yu B. 2012. Uridylation of miRNAs by HEN1 SUPPRESSOR1 in *Arabidopsis*. Curr Biol. 22:695–700. doi:10.1016/j.cub.2012.02.052

Rissland OS, Mikulasova A, Norbury CJ. 2007. Efficient RNA polyuridylation by noncanonical poly(A) polymerases. Mol Cell Biol. 27:3612-3624. doi:10.1128/MCB.02209-06.

Rissland OS, Norbury CJ. 2009. Decapping is preceded by 3′ uridylation in a novel pathway of bulk mRNA turnover. Nat Struct Mol Biol. 16:616–623. doi:10.1038/nsmb.1601

Risso D, Ngai J, Speed TP, Dudoit S. 2014. Normalization of RNA-seq data using factor analysis of control genes or samples. Nat Biotech. 32:896–902. doi:10.1038/nbt.2931

Risso D, Schwartz K, Sherlock G, Dudoit S. 2011. GC-content normalization for RNA-seq data. BMC Bioinformatics. 12:480. doi:10.1186/1471-2105-12-480.

Robinson MD, McCarthy DJ, Smyth GK. 2010. edgeR: a Bioconductor package for differential expression analysis of digital gene expression data. Bioinformatics. 26:139–140. doi:10.1093/bioinformatics/btp616.

Scheer H, Zuber H, De Almeida C, Gagliardi D. 2016. Uridylation earmarks mRNAs for degradation… and more. Trends in Genet. 32:607–619. doi:10.1016/j.tig.2016.08.003.

Scheer H, de Almeida C, Ferrier E, Simonnot Q, Poirier L, Pflieger D, Sement FM, Koechler S, Piermaria C, Krawczyk P, Mroczek S, Chicher J, Kuhn L, Dziembowski A, Hammann P, Zuber H, Gagliardi D. 2021. The TUTase URT1 connects decapping activators and prevents the accumulation of excessively deadenylated mRNAs to avoid siRNA biogenesis. Nat Commun. 12:1298. doi:10.1038/s41467-021-21382-2.

Schindelin J, Arganda-Carreras I, Frise E, Kaynig V, Longair M, Pietzsch T, Preibisch S, Rueden C, Saalfeld S, Schmid B, Tinevez J-Y, White DJ, Hartenstein V, Eliceiri K, Tomancak P, Cardona A. 2012. Fiji: an open-source platform for biological-image analysis. Nat Methods. 9:676–682. doi:10.1038/nmeth.2019.

Schmidt M-J, West S, Norbury CJ. 2010. The human cytoplasmic RNA terminal U-transferase ZCCHC11 targets histone mRNAs for degradation. RNA. doi:10.1261/rna.2252511.

Serizay J, Dong Y, Jänes J, Chesney M, Cerrato C, Ahringer J. 2020. Distinctive regulatory architectures of germline-active and somatic genes in *C. elegans*. Genome Res. 30:1752–1765. doi:10.1101/gr.265934.120.

Seroussi U, Lugowski A, Wadi L, Lao RX, Willis AR, Zhao W, Sundby AE, Charlesworth AG, Reinke AW, Claycomb JM. 2023. A comprehensive survey of *C. elegans* argonaute proteins reveals organism-wide gene regulatory networks and functions. eLife. 12:e83853. doi:10.7554/eLife.83853.

Shen B, Goodman HM. 2004. Uridine addition After microRNA-directed cleavage. Science. 306:997–997. doi:10.1126/science.1103521.

Shukla A, Yan J, Pagano DJ, Dodson AE, Fei Y, Gorham J, Seidman JG, Wickens M, Kennedy S. 2020. poly(UG)-tailed RNAs in genome protection and epigenetic inheritance. Nature. 582:283–288. doi:10.1038/s41586-020-2323-8.

Spracklin G, Fields B, Wan G, Becker D, Wallig A, Shukla A, Kennedy S. 2017. The RNAi inheritance machinery of *Caenorhabditis elegans*. Genetics. 206:1403–1416. doi:10.1534/genetics.116.198812.

Thornton JE, Du P, Jing L, Sjekloca L, Lin S, Grossi E, Sliz P, Zon LI, Gregory RI. 2014. Selective microRNA uridylation by Zcchc6 (TUT7) and Zcchc11 (TUT4). Nucleic Acids Res. 42:11777–11791. doi:10.1093/nar/gku805.

van Wolfswinkel JC, Claycomb JM, Batista PJ, Mello CC, Berezikov E, Ketting RF. 2009. CDE-1 affects chromosome segregation through uridylation of CSR-1-bound siRNAs. Cell. 139:135–148. doi:10.1016/j.cell.2009.09.012.

Vieux K-F, Prothro KP, Kelley LH, Palmer C, Maine EM, Veksler-Lublinsky I, McJunkin K. 2021. Screening by deep sequencing reveals mediators of microRNA tailing in *C. elegans*. Nucleic Acids Res. 49:11167–11180. doi:10.1093/nar/gkab840.

Wahba L, Hansen L, Fire AZ. 2021. An essential role for the piRNA pathway in regulating the ribosomal RNA pool in *C. elegans*. Dev Cell. 56:2295-2312.e6. doi:10.1016/j.devcel.2021.07.014.

Wang X, Kong W, Wang Y, Wang J, Zhong L, Lao K, Dong X, Zhang D, Huang H, Mo B, Yu Y, Ren G. 2022. Uridylation and the SKI complex orchestrate the Calvin cycle of photosynthesis through

RNA surveillance of TKL1 in *Arabidopsis*. Proc Nat Acad Sci USA. 119:e2205842119. doi:10.1073/pnas.2205842119.

Wang Y, Weng C, Chen X, Zhou X, Huang X, Yan Y, Zhu C. 2020. CDE-1 suppresses the production of risiRNA by coupling polyuridylation and degradation of rRNA. BMC Biol. 18:115. doi:10.1186/s12915-020-00850-z.

Warkocki Z, Krawczyk PS, Adamska D, Bijata K, Garcia-Perez JL, Dziembowski A. 2018. Uridylation by TUT4/7 restricts retrotransposition of human LINE-1s. Cell. 174:1537-1548.e29. doi:10.1016/j.cell.2018.07.022.

Wu D, Pedroza M, Chang J, Dean J. 2023. DIS3L2 ribonuclease degrades terminal-uridylated RNA to ensure oocyte maturation and female fertility. Nucleic Acids Res. **51**:3078–3093. doi:10.1093/nar/gkad061.

Xu F, Feng X, Chen X, Weng C, Yan Q, Xu T, Hong M, Guang S. 2018. A cytoplasmic Argonaute protein promotes the inheritance of RNAi. Cell Rep.23:2482–2494. doi:10.1016/j.celrep.2018.04.072.

Yang A, Bofill-De Ros X, Shao T-J, Jiang M, Li K, Villanueva P, Dai L, Gu S. 2019. 3′ uridylation confers miRNAs with non-canonical target repertoires. Mol Cell. 75:511-522.e4. doi:10.1016/j.molcel.2019.05.014.

Yang A, Bofill-De Ros X, Stanton R, Shao T-J, Villanueva P, Gu S. 2022. TENT2, TUT4, and TUT7 selectively regulate miRNA sequence and abundance. Nat Commun. 13:5260. doi:10.1038/s41467-022-32969-8.

Yang A, Shao T-J, Bofill-De Ros X, Lian C, Villanueva P, Dai L, Gu S. 2020. AGO-bound mature miRNAs are oligouridylated by TUTs and subsequently degraded by DIS3L2. Nat Commun. 11:2765. doi:10.1038/s41467-020-16533-w.

Zhao M-Z, Lin D-H, Zuo H, Wei H, Wang X, Gou L-T, Liu M-F. 2022. piRNA 3′ uridylation facilitates the assembly of MIWI/piRNA complex for efficient target regulation in mouse male germ cells. Cell Res. 32:1030–1033. doi:10.1038/s41422-022-00659-1.

Zhou X, Feng X, Mao H, Li M, Xu F, Hu K, Guang S. 2017. RdRP-synthesized antisense ribosomal siRNAs silence pre-rRNA via the nuclear RNAi pathway. Nat Struct Mol Biol. 24:258–269. doi:10.1038/nsmb.3376.

Figure legends

Fig. 1. PUP-4 is expressed in germline and somatic tissues. (A) Representative protein blot containing whole-protein extracts from adult hermaphrodites raised at 25°C (see Materials and Methods). Blot was probed with anti-FLAG to visualize PUP-4::3xFLAG and anti-actin as a loading control. "+" indicates the listed allele is present in the strain. Quantification is shown for n = 5 biological replicates. Statistical significance was evaluated with a Dunnet's test followed by Tukey's multiple correction. *, $p < 0.05$. PUP-4::3xFLAG signal in each strain was normalized to the average *pup-4::3xflag; pup-1/-2(+) glp-1(+)* control value. (B) Representative protein blot containing extracts prepared from 25°C F2 adult hermaphrodites and dissected adult hermaphrodite gonads. Protein extract was prepared from 52 wildtype animals, 102 wildtype gonad arms, 92 *pup-4::3xflag* animals, and 193 *pup-4::3xflag* gonad arms. Blot was probed with anti-FLAG and reprobed with anti-beta-tubulin. Quantification is shown for n= 3 biological replicates; PUP-4::3xFLAG signal in gonads was normalized to the signal in *pup-4::3xflag* whole animals. Note that gonad tissue is primarily germline. (C) Representative protein blot of extracts from adult hermaphrodites raised at 22°C. Quantification is shown for n = 3 biological replicates. (D) Representative protein blots containing whole-protein extracts prepared from adult hermaphrodites of the genotype indicated raised at 22°C. "+" indicates the listed allele is present

in the strain. n = 3 biological replicates of each blot. Wildtype and PUP-1 lanes are from the same blot. Statistical significance in B, C, and D was evaluated using a two-sided Student's *t*-test. For all boxplots, box indicates the middle 50% of values, bar indicates the median, yellow diamond indicates the average.

Fig. 2. Altered sRNA U-tailing in *pup* mutants. (A) Proportion of U-tailed reads for high confidence uridylation targets in wildtype and each *pup* mutant. sRNAs that pass both the abundance cutoff and overlap filter criteria (4,776) are included (see Fig. S2). Each point represents an sRNA. Wildtype uridylation frequencies for all sRNAs are indicated in gray; only high confidence uridylation targets in each mutant strain (n) are shown. Purple points, sRNAs with reduced uridylation frequency in the indicated mutant; gold points, sRNAs with increased uridylation frequency. White diamond, average U-tail frequency for each group of sRNAs; value is listed below in the corresponding color. (B) UpSet plot (Lex *et al.* 2014) indicates shared and unique sRNA uridylation targets identified in different mutant strains; the top 10 intersections (out of 171) are shown. Purple, down-uridylated in mutant; gold, up-uridylated in mutant. (C) Proportion of U-tailed reads is plotted for sRNAs indicated in individual columns in panel B as indicated by Roman numerals. Gray points indicate the uridylation frequency of those sRNAs in wildtype. X-axis, genotype. Y-axis, proportion of U-tailed reads. White diamond, average U-tail frequency for each group of sRNAs; value is listed below in the corresponding color. Dotted line, average uridylation frequency of the 4,776 sRNAs in wildtype (~14%). The siRNAs shown here correspond to protein-coding genes; U-tail frequency of siRNAs corresponding to transposons are shown in Fig. S5. (D) Protein sequence alignment and neighbor-joining analysis of F43E2.1/PUP-4 and closely related *C. elegans* proteins. Value on each branch indicates the average number of substitutions per site. (E) Schematic of PUP proteins showing relative positions of conserved domains. NTD, nucleotidyl transferase domain of poly(A) polymerase and TUTase; contains catalytic residues for transferring a nucleotide to RNA 3' end. "TUTase NTD" in PUP-1 lacks catalytic residues. PAPa, poly(A) polymerase-associated domain containing a nucleotide recognition motif (NRM). TRF4, yeast DNA polymerase sigma homology region including an NTD and NRM. *, degenerate NRM. White boxed regions are predicted to be

intrinsically disordered.

Fig. 3. Loss of PUP activity profoundly alters sRNA abundance. All points represent sRNAs whose U-tailing is consistently reduced in the indicated mutant (high confidence uridylation targets). Differentially abundant sRNAs ($|\log_2 FC| > 1$ & $FDR < 0.05$) are indicated in blue (downregulated) and red (upregulated). The number of DA sRNAs is listed for each genotype. Black points indicate sRNAs that are not differentially abundant. X-axis, $\log_2$FC of abundance (mutant/wildtype). Y-axis, $\log_2$FC of uridylation frequency (mutant/wildtype) (see Materials and Methods). Triangles, indicating sRNAs with $\log_2$FC(abundance) values <|6|, are plotted at their corresponding $\log_2$FC(uridylation) value.

Fig. 4. Changes in sRNA abundance parsed by Argonaute association. (A) U-tail frequencies for sRNAs present at ≥10 CPM in wildtype adult hermaphrodites, sorted based on documented associations with individual Argonaute proteins (Seroussi *et al.* 2023). X-axis, Argonaute proteins. Y-axis, proportion of U-tailed reads for corresponding sRNAs in our wildtype dataset. Each point represents an sRNA. sRNAs that associate with multiple Argonautes are represented in each Argonaute group. "Not enriched," sRNAs without a reported Argonaute preference. Dashed line, average uridylation frequency (11%) for the 9,645 Argonaute-associated sRNAs in wildtype. Red bar, average U-tail frequency for each group of Argonaute-associated sRNAs; this value and number of sRNAs associated with each Argonaute are listed below. (B) High confidence uridylated sRNAs that are DA in the indicated genotypes are plotted by Argonaute association. X-axis, $\log_2$FC of abundance (mutant/wildtype). Y-axis, $\log_2$FC of uridylation frequency (mutant/wildtype). Blue points, downregulated sRNAs; red points, upregulated sRNAs; black points, sRNAs without a statistically significant change in abundance. Values are listed in each panel. $|\log_2 FC| > 1$ & $FDR < 0.05$. Remaining Argonaute associations are shown in Fig. S10.

Fig. 5. The mRNA targets of high confidence uridylated sRNAs and differentially abundant sRNAs have primary expression in the germline or ubiquitous expression. (A) Gene Ontology analysis of

mRNAs targeted by high confidence U-tailed sRNAs that are differentially abundant in any *pup* mutant. Left, genes targeted by reduced abundance sRNAs (636) encode products enriched for 9 GO terms. Right, genes targeted by increased abundance sRNA (779) encode products enriched for 17 GO terms. Inserts indicate the quadrant in Fig. 3 plots corresponding to the differentially abundant sRNAs used in the GO analysis. (B) Tissue expression patterns for genes targeted by sRNAs with reduced (4,609, left) or increased (654, right) uridylation in any *pup* mutant (regardless of abundance). (C) Tissue expression patterns for genes targeted by differentially abundant sRNAs in any mutant with reduced (2,745, left) or increased (2,548, right) abundance as represented in Fig. S8 (regardless of uridylation). Tissue expression data are from Serizay *et al.* (2020). Tissue specificity index was calculated based on cummerBund's S function; Chi-square test (**p*-value < 0.01, ***p*-value < 0.001, ****p*-value < 0.0001) was used to calculate significance.

Fig. 6. mRNA U-tail frequency and length are reduced in *pup-3(0)* mutants. (A) mRNA 3' tail frequencies in wildtype and *pup* mutant adults as detected with *tailfindr* analysis of Nano3P-seq data. Plots indicate the average % of strictly A-tailed reads, strictly U-tailed reads, and A+U-tailed reads out of the total number of reads obtained. White points, average % tailed reads for each replicate. Y-axis, % tailed reads; x-axis, genotype. Statistical significance for each mutant compared to wildtype was determined by paired *t*-test (**p*-value < 0.02, ***p*-value < 0.01). (B, C) The distribution of A- and U-tail lengths in wildtype and *pup* mutants. Each point represents the average tailing frequency of all mRNAs with tails of a certain length. For the 4,183 transcripts present at ≥10 CPM, a cutoff of 50 reads/length group was implemented. X-axes, tail length in nucleotides. Y-axes, percentage of tailed reads. Dashed line/number, average tail length. Black points, most common tail length(s). (C) Although 3 nt is the most common U-tail length detected for any genotype, note that *tailfindr* cannot identify shorter U-tails (see text).

Fig. 7. Differentially abundant mRNAs are most prominent in the *pup-3(0);pup-4(0);pup-1/-2(0)* quadruple mutant. (A) Plots show DA mRNAs detected in each genotype. Each point represents mRNA from a single gene. Blue, downregulated mRNAs; red, upregulated mRNAs; gray, no statistical change in abundance. Numbers of DA mRNAs are provided. X-axis, log_2FC in mutant

versus wildtype. Y-axis, $-\log_{10}$(p-value). Statistical significance was determined as $|\log_2 FC| > 1$ and FDR < 0.01. (B) UpSet plot shows numbers of unique and shared differentially abundant mRNAs identified in *pup* mutant strains; the top 10 intersections are shown (out of 32). Blue, downregulated mRNAs; red, upregulated mRNAs. (C) Bar plots indicate the % U-tailed reads for mRNAs that are differentially abundant in (upper) *pup-3(0)* single or (lower) *pup-3(0);pup-4(0);pup-1/-2(0)* quadruple mutant compared to wildtype. n, number of mRNAs in each group. Average % U-tailing is indicated under each bar. Dashed line, average % U-tailing in wildtype from Fig. 6A (second panel) for the 4,183 transcripts present at ≥ 10 CPM.

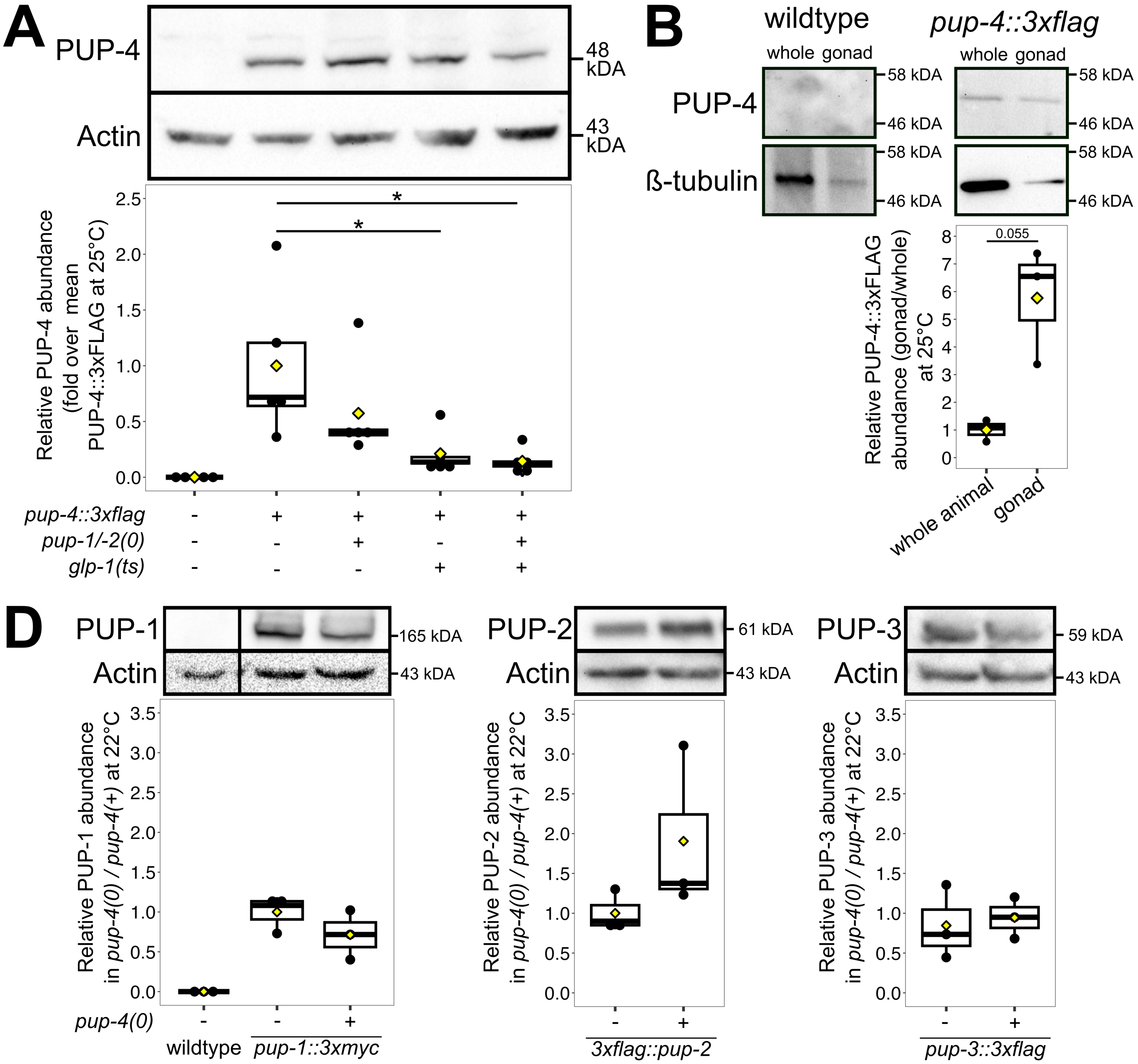

A
PUP-4
Actin
48 kDA
43 kDA
Relative PUP-4 abundance (fold over mean PUP-4::3xFLAG at 25°C)
pup-4::3xflag
pup-1/-2(0)
glp-1(ts)
B
wildtype
pup-4::3xflag
whole gonad
PUP-4
ß-tubulin
58 kDA
46 kDA
0.055
Relative PUP-4::3xFLAG abundance (gonad/whole) at 25°C
whole animal
gonad
C
Relative PUP-4 abundance (fold over mean PUP-4::3xFLAG at 22°C)
pup-4::3xflag
pup-4::3xflag;pup-1/-2(0)
D
PUP-1
Actin
165 kDA
43 kDA
Relative PUP-1 abundance in pup-4(0) / pup-4(+) at 22°C
pup-4(0)
wildtype
pup-1::3xmyc
PUP-2
61 kDA
Relative PUP-2 abundance in pup-4(0) / pup-4(+) at 22°C
3xflag::pup-2
PUP-3
59 kDA
Relative PUP-3 abundance in pup-4(0) / pup-4(+) at 22°C
pup-3::3xflag

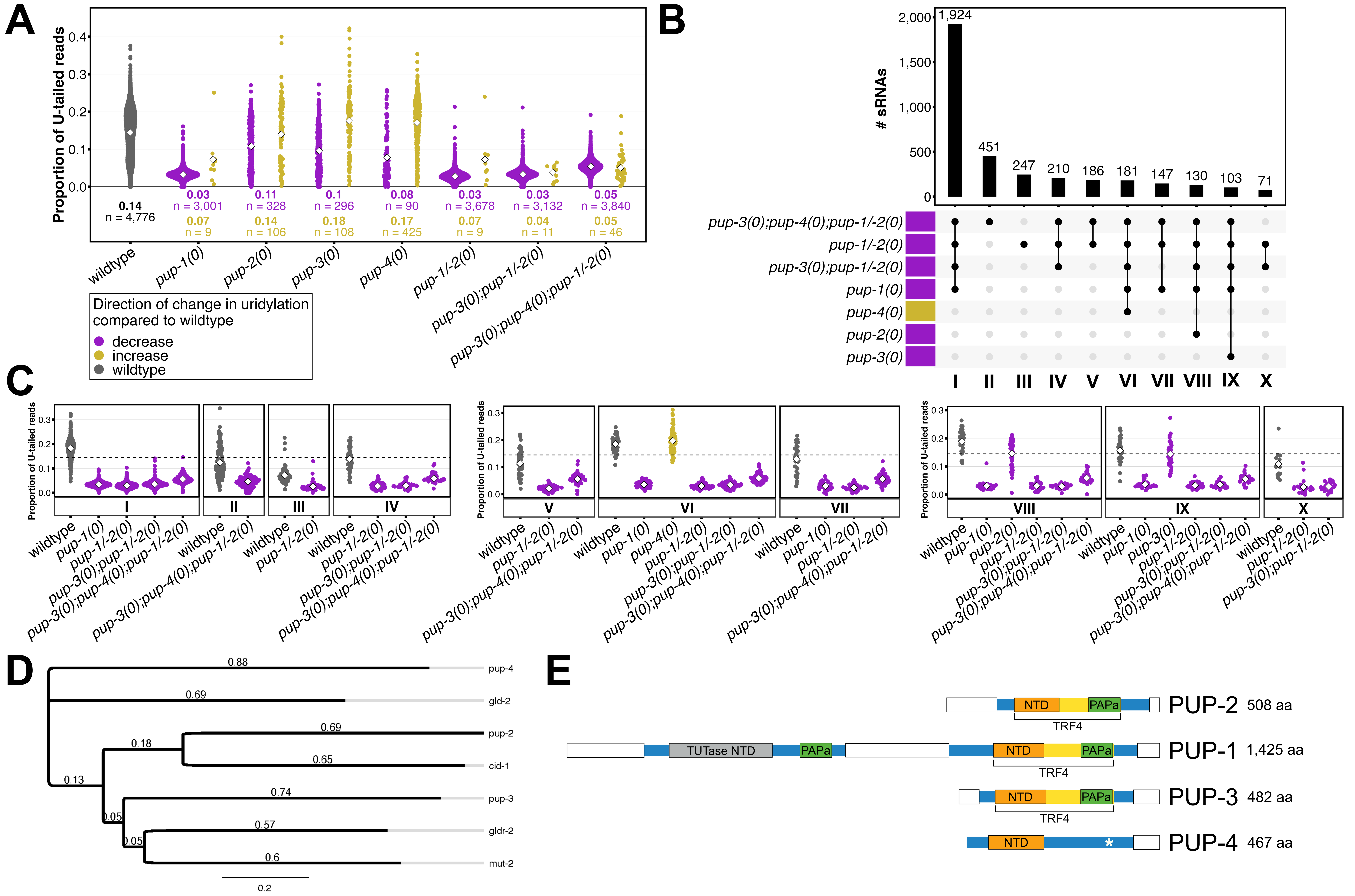

A
Proportion of U-tailed reads
0.0
0.1
0.2
0.3
0.4
0.14
n = 4,776
0.03
n = 3,001
0.07
n = 9
0.11
n = 328
0.14
n = 106
0.1
n = 296
0.18
n = 108
0.08
n = 90
0.17
n = 425
0.03
n = 3,678
0.07
n = 9
0.03
n = 3,132
0.04
n = 11
0.05
n = 3,840
0.05
n = 46
wildtype
pup-1(0)
pup-2(0)
pup-3(0)
pup-4(0)
pup-1/-2(0)
pup-3(0);pup-1/-2(0)
pup-3(0);pup-4(0);pup-1/-2(0)
Direction of change in uridylation compared to wildtype
decrease
increase
wildtype
B
sRNAs
0
500
1,000
1,500
2,000
1,924
451
247
210
186
181
147
130
103
71
pup-3(0);pup-4(0);pup-1/-2(0)
pup-1/-2(0)
pup-3(0);pup-1/-2(0)
pup-1(0)
pup-4(0)
pup-2(0)
pup-3(0)
I
II
III
IV
V
VI
VII
VIII
IX
X
C
Proportion of U-tailed reads
0.0
0.1
0.2
0.3
I
II
III
IV
V
VI
VII
VIII
IX
X
wildtype
pup-1(0)
pup-2(0)
pup-3(0)
pup-4(0)
pup-1/-2(0)
pup-3(0);pup-1/-2(0)
pup-3(0);pup-4(0);pup-1/-2(0)
D
0.88
pup-4
0.69
gld-2
0.69
pup-2
0.18
0.65
cid-1
0.13
0.74
pup-3
0.05
0.57
gldr-2
0.05
0.6
mut-2
0.2
E
NTD
PAPa
TRF4
PUP-2 508 aa
TUTase NTD
PAPa
NTD
PAPa
TRF4
PUP-1 1,425 aa
NTD
PAPa
TRF4
PUP-3 482 aa
NTD
*
PUP-4 467 aa

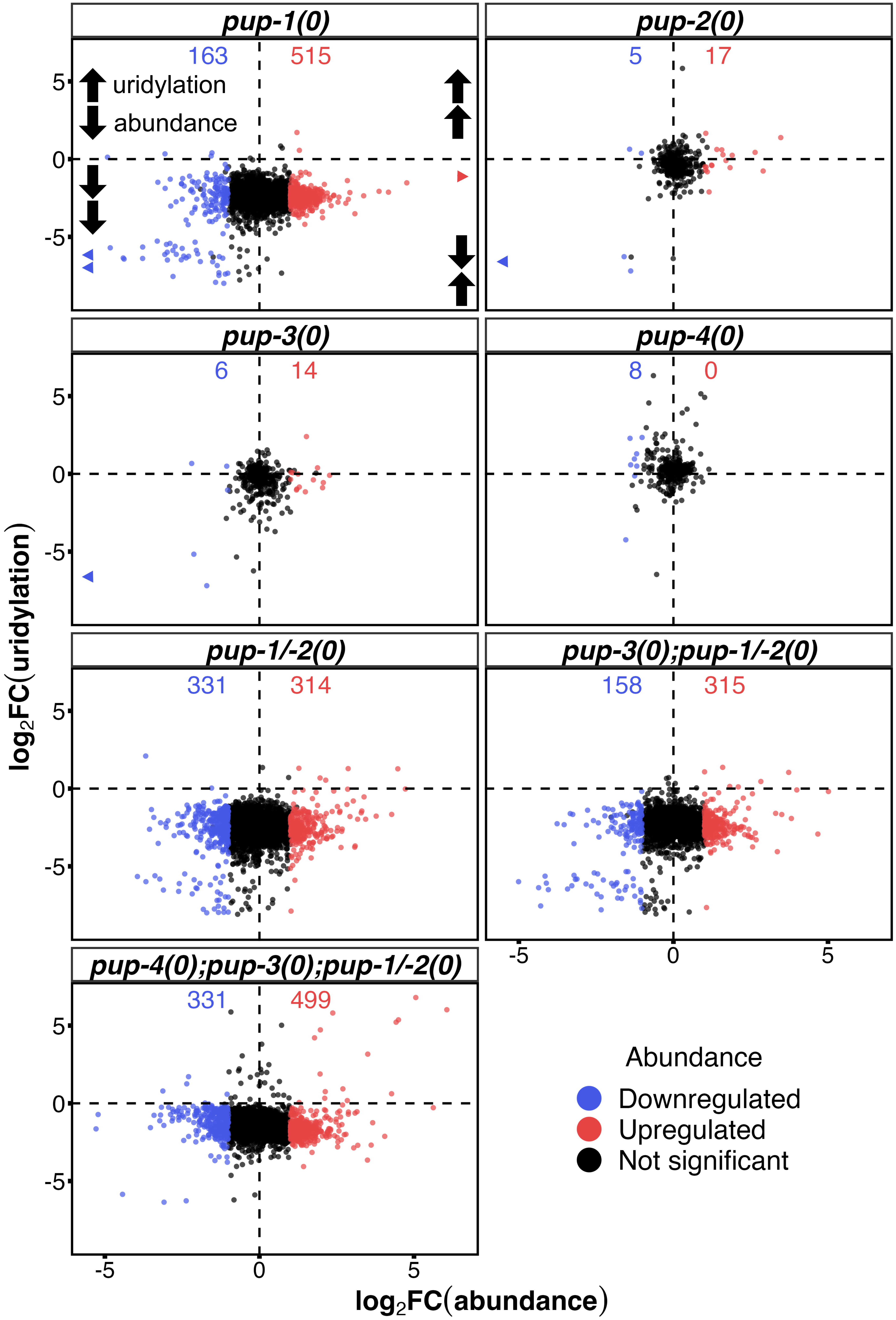

pup-1(0)
163
515
uridylation
abundance
pup-2(0)
5
17
pup-3(0)
6
14
pup-4(0)
8
0
pup-1/-2(0)
331
314
pup-3(0);pup-1/-2(0)
158
315
pup-4(0);pup-3(0);pup-1/-2(0)
331
499
Abundance
Downregulated
Upregulated
Not significant
log2FC(uridylation)
log2FC(abundance)
5
0
-5

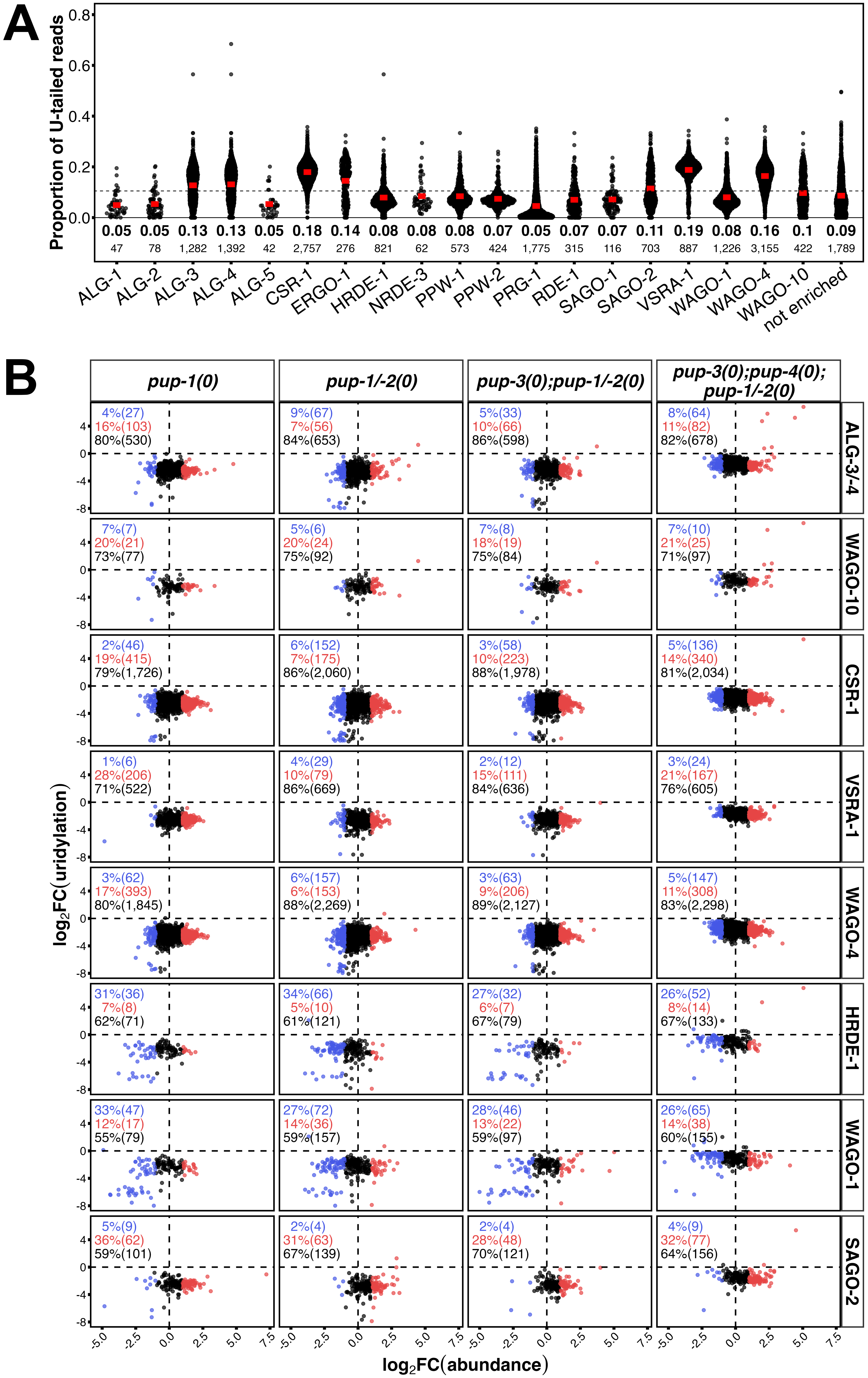
A
Proportion of U-tailed reads
0.05 0.05 0.13 0.13 0.05 0.18 0.14 0.08 0.08 0.08 0.07 0.05 0.07 0.07 0.11 0.19 0.08 0.16 0.1 0.09
47 78 1,282 1,392 42 2,757 276 821 62 573 424 1,775 315 116 703 887 1,226 3,155 422 1,789
ALG-1 ALG-2 ALG-3 ALG-4 ALG-5 CSR-1 ERGO-1 HRDE-1 NRDE-3 PPW-1 PPW-2 PRG-1 RDE-1 SAGO-1 SAGO-2 VSRA-1 WAGO-1 WAGO-4 WAGO-10 not enriched
B
pup-1(0)
pup-1/-2(0)
pup-3(0);pup-1/-2(0)
pup-3(0);pup-4(0); pup-1/-2(0)
ALG-3/4
4%(27) 16%(103) 80%(530)
9%(67) 7%(56) 84%(653)
5%(33) 10%(66) 86%(598)
8%(64) 11%(82) 82%(678)
WAGO-10
7%(7) 20%(21) 73%(77)
5%(6) 20%(24) 75%(92)
7%(8) 18%(19) 75%(84)
7%(10) 21%(25) 71%(97)
CSR-1
2%(46) 19%(415) 79%(1,726)
6%(152) 7%(175) 86%(2,060)
3%(58) 10%(223) 88%(1,978)
5%(136) 14%(340) 81%(2,034)
VSRA-1
1%(6) 28%(206) 71%(522)
4%(29) 10%(79) 86%(669)
2%(12) 15%(111) 84%(636)
3%(24) 21%(167) 76%(605)
WAGO-4
3%(62) 17%(393) 80%(1,845)
6%(157) 6%(153) 88%(2,269)
3%(63) 9%(206) 89%(2,127)
5%(147) 11%(308) 83%(2,298)
HRDE-1
31%(36) 7%(8) 62%(71)
34%(66) 5%(10) 61%(121)
27%(32) 6%(7) 67%(79)
26%(52) 8%(14) 67%(133)
WAGO-1
33%(47) 12%(17) 55%(79)
27%(72) 14%(36) 59%(157)
28%(46) 13%(22) 59%(97)
26%(65) 14%(38) 60%(155)
SAGO-2
5%(9) 36%(62) 59%(101)
2%(4) 31%(63) 67%(139)
2%(4) 28%(48) 70%(121)
4%(9) 32%(77) 64%(156)
log2FC(uridylation)
log2FC(abundance)

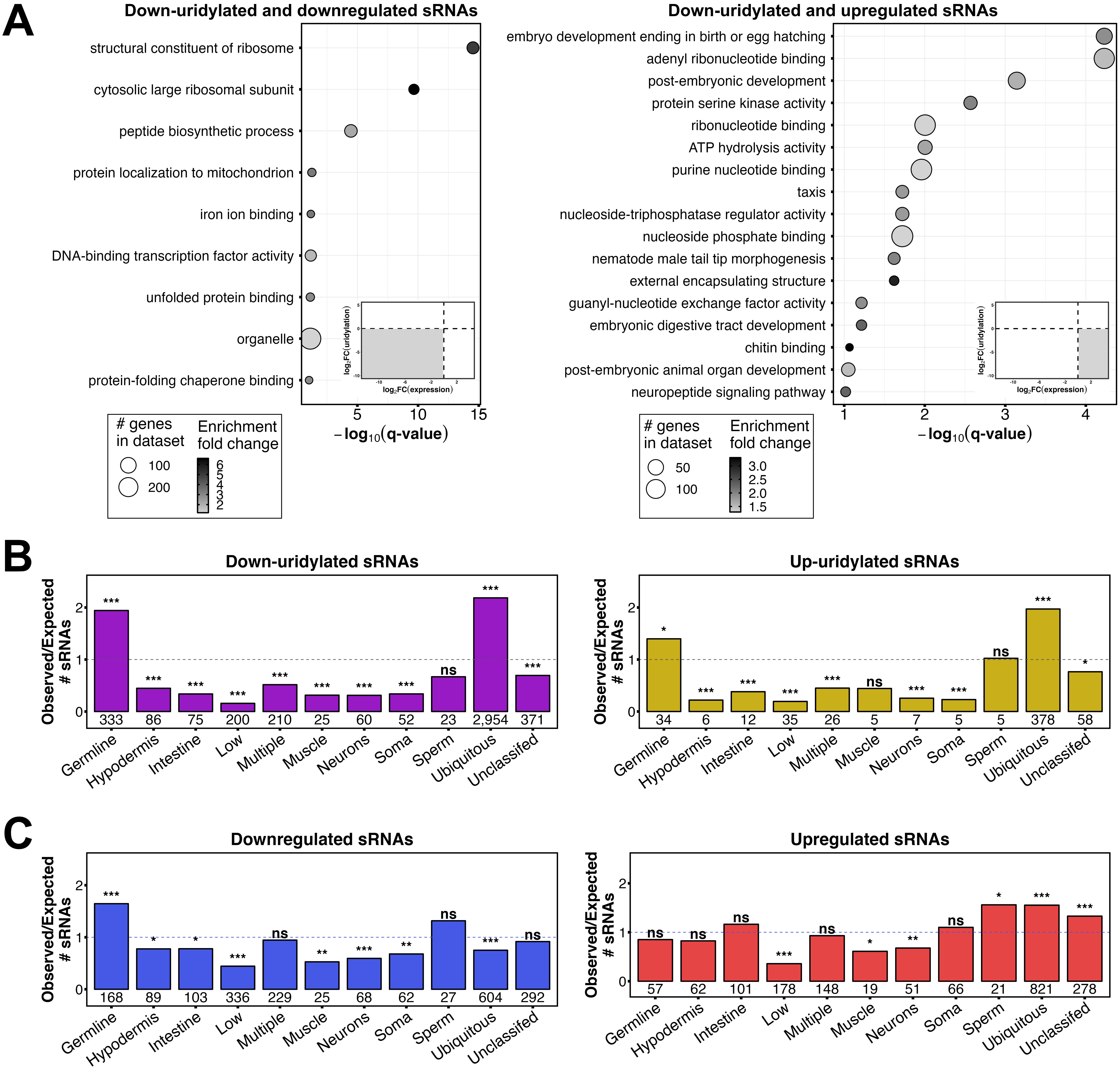

A
Down-uridylated and downregulated sRNAs
structural constituent of ribosome
cytosolic large ribosomal subunit
peptide biosynthetic process
protein localization to mitochondrion
iron ion binding
DNA-binding transcription factor activity
unfolded protein binding
organelle
protein-folding chaperone binding
−log10(q-value)
genes in dataset
Enrichment fold change
Down-uridylated and upregulated sRNAs
embryo development ending in birth or egg hatching
adenyl ribonucleotide binding
post-embryonic development
protein serine kinase activity
ribonucleotide binding
ATP hydrolysis activity
purine nucleotide binding
taxis
nucleoside-triphosphatase regulator activity
nucleoside phosphate binding
nematode male tail tip morphogenesis
external encapsulating structure
guanyl-nucleotide exchange factor activity
embryonic digestive tract development
chitin binding
post-embryonic animal organ development
neuropeptide signaling pathway
log2FC(uridylation)
log2FC(expression)
B
Down-uridylated sRNAs
Up-uridylated sRNAs
Observed/Expected # sRNAs
Germline
Hypodermis
Intestine
Low
Multiple
Muscle
Neurons
Soma
Sperm
Ubiquitous
Unclassifed
333 86 75 200 210 25 60 52 23 2,954 371
34 6 12 35 26 5 7 5 5 378 58
C
Downregulated sRNAs
Upregulated sRNAs
168 89 103 336 229 25 68 62 27 604 292
57 62 101 178 148 19 51 66 21 821 278

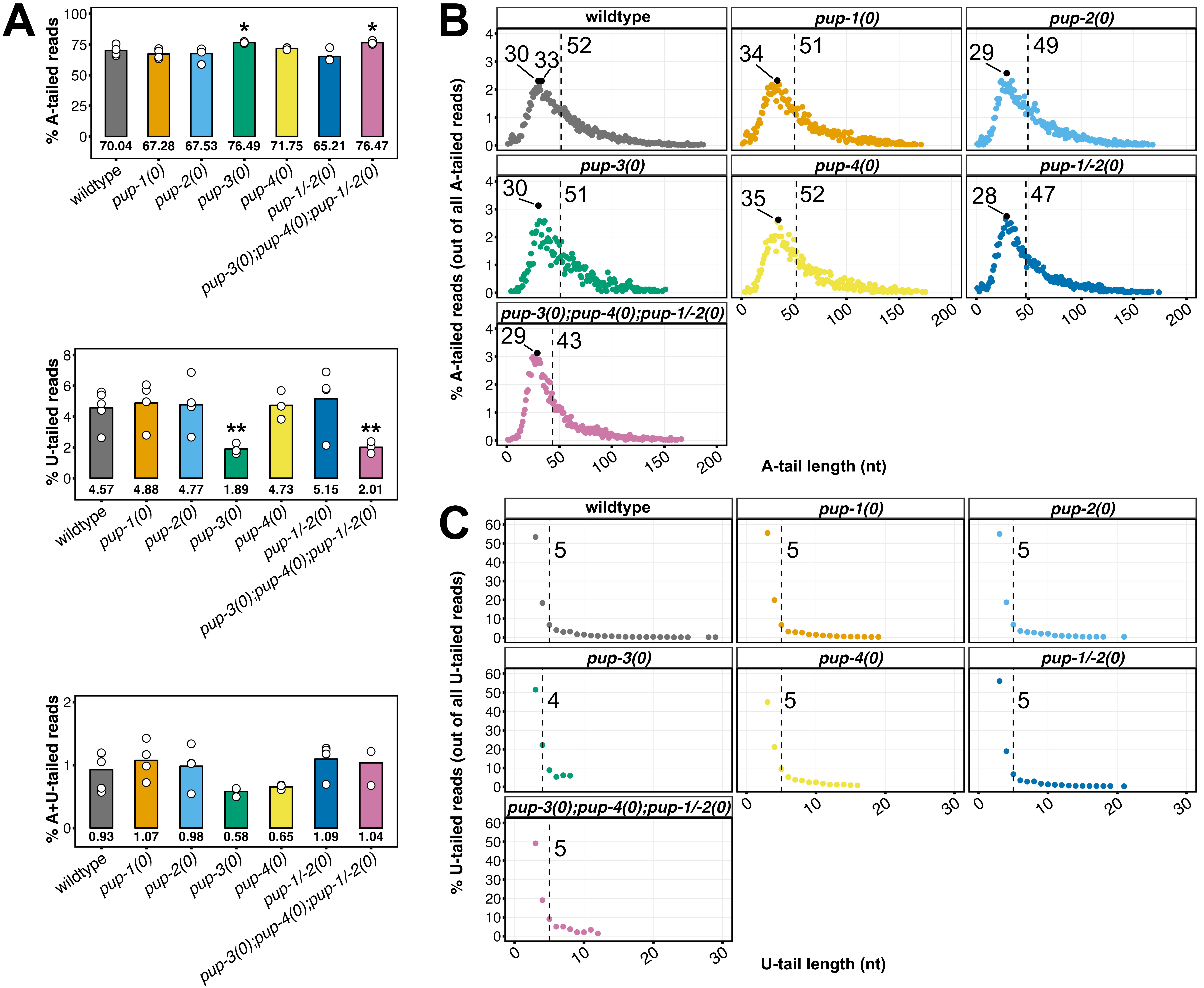

A
% A-tailed reads
70.04 67.28 67.53 76.49 71.75 65.21 76.47
wildtype
pup-1(0)
pup-2(0)
pup-3(0)
pup-4(0)
pup-1/-2(0)
pup-3(0);pup-4(0);pup-1/-2(0)
% U-tailed reads
4.57 4.88 4.77 1.89 4.73 5.15 2.01
% A+U-tailed reads
0.93 1.07 0.98 0.58 0.65 1.09 1.04
B
% A-tailed reads (out of all A-tailed reads)
A-tail length (nt)
C
% U-tailed reads (out of all U-tailed reads)
U-tail length (nt)

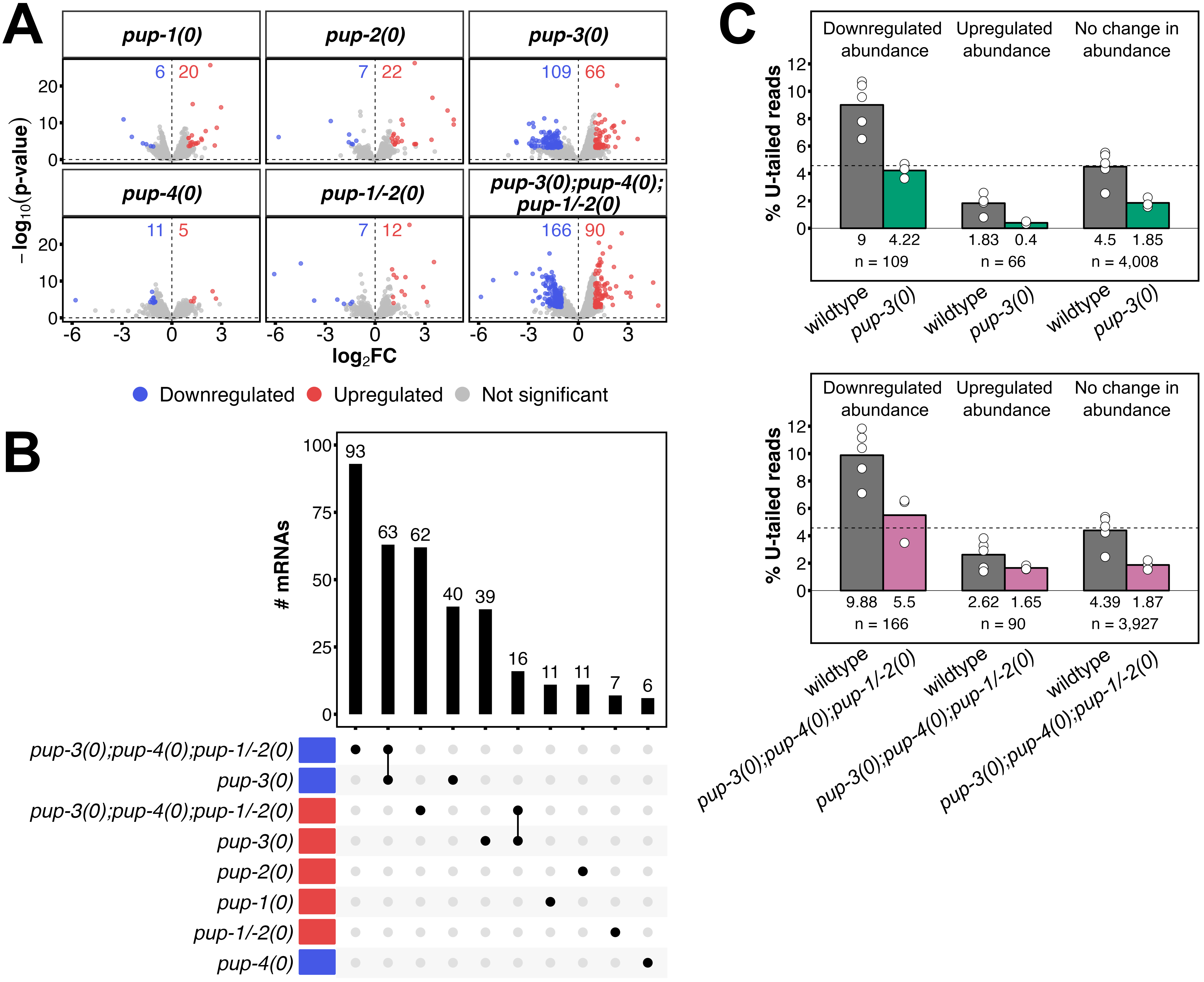
A
pup-1(0)
pup-2(0)
pup-3(0)
pup-4(0)
pup-1/-2(0)
pup-3(0);pup-4(0);
pup-1/-2(0)
6 20
7 22
109 66
11 5
7 12
166 90
−log10(p-value)
log2FC
Downregulated
Upregulated
Not significant
B
mRNAs
93
63
62
40
39
16
11
11
7
6
pup-3(0);pup-4(0);pup-1/-2(0)
pup-3(0)
pup-3(0);pup-4(0);pup-1/-2(0)
pup-3(0)
pup-2(0)
pup-1(0)
pup-1/-2(0)
pup-4(0)
C
Downregulated abundance
Upregulated abundance
No change in abundance
% U-tailed reads
9 4.22
n = 109
1.83 0.4
n = 66
4.5 1.85
n = 4,008
wildtype
pup-3(0)
9.88 5.5
n = 166
2.62 1.65
n = 90
4.39 1.87
n = 3,927
pup-3(0);pup-4(0);pup-1/-2(0)
