# Supplementary material for: Poly(U) polymerase activity in Caenorhabditis elegans regulates abundance and tailing of sRNA and mRNA
Source: Genetics. 2024 Jul 28;228(2):iyae120. doi: 10.1093/genetics/iyae120 (PMC11457939; doi:10.1093/genetics/iyae120)
Supplement: iyae120_Supplementary_Data [file iyae120_supplementary_data.zip › Supplemental_Figures_GENETICS-2024-307061.pdf]

Supplemental figures

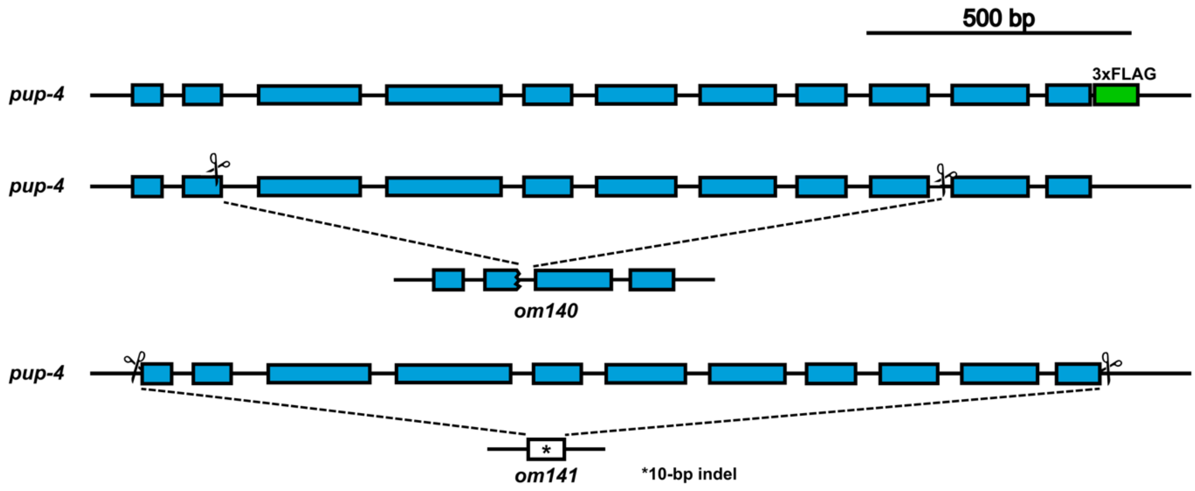

Fig. S1. Locations of CRISPR-Cas9 edits in the *pup-4* gene. *pup-4* intron/exon structure (5' left, 3' right), location of *3xflag* insertion in epitope-tagged allele, and location of deletion breakpoints in *om140* and *om141* alleles are shown. Boxes, exons; lines, introns and 5' and 3' untranslated regions. Breakpoints are indicated with a scissors symbol in the full-length gene illustration.

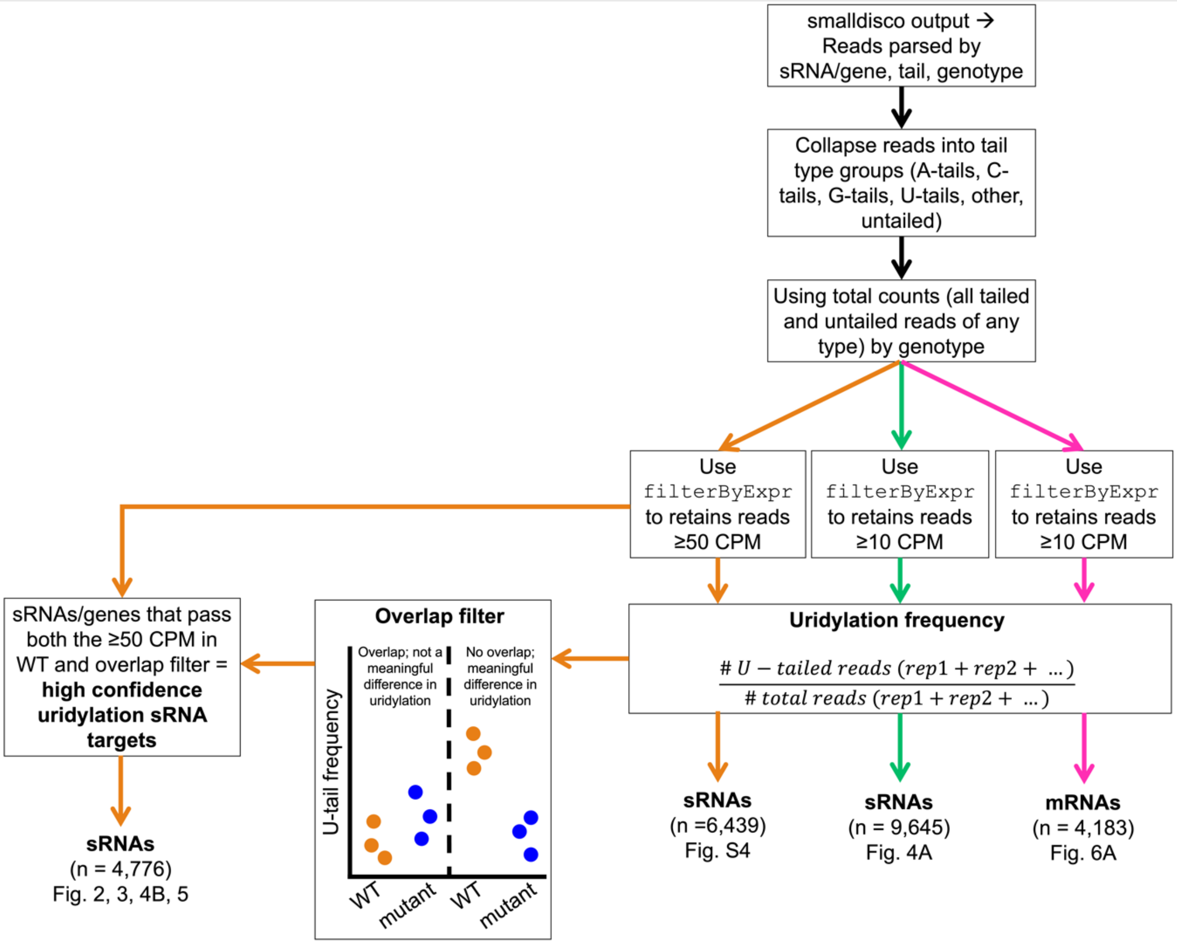

Fig. S2. Workflow used to identify sRNAs with consistently reduced U-tailing (PUP sRNA targets) in *pup* mutants compared to wildtype. See Materials and Methods for additional details. We refer to sRNAs that passed both the ≥50 CPM and overlap filter criteria as high confidence uridylation targets.

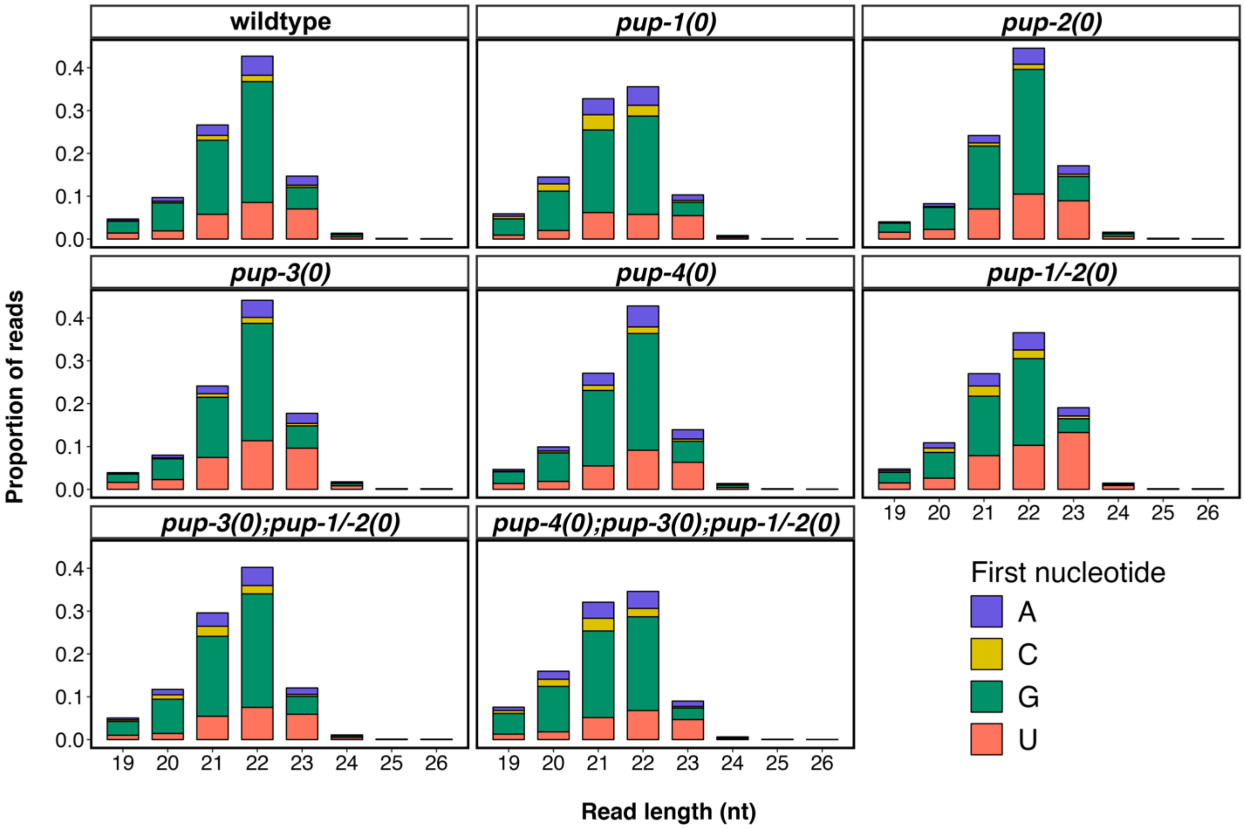

Fig. S3. Distribution of sRNA read length and first nucleotide. X-axis, proportion of reads out of all sRNA species detected for each genotype. Y-axis, read length (nt). Colors indicate the first nucleotide.

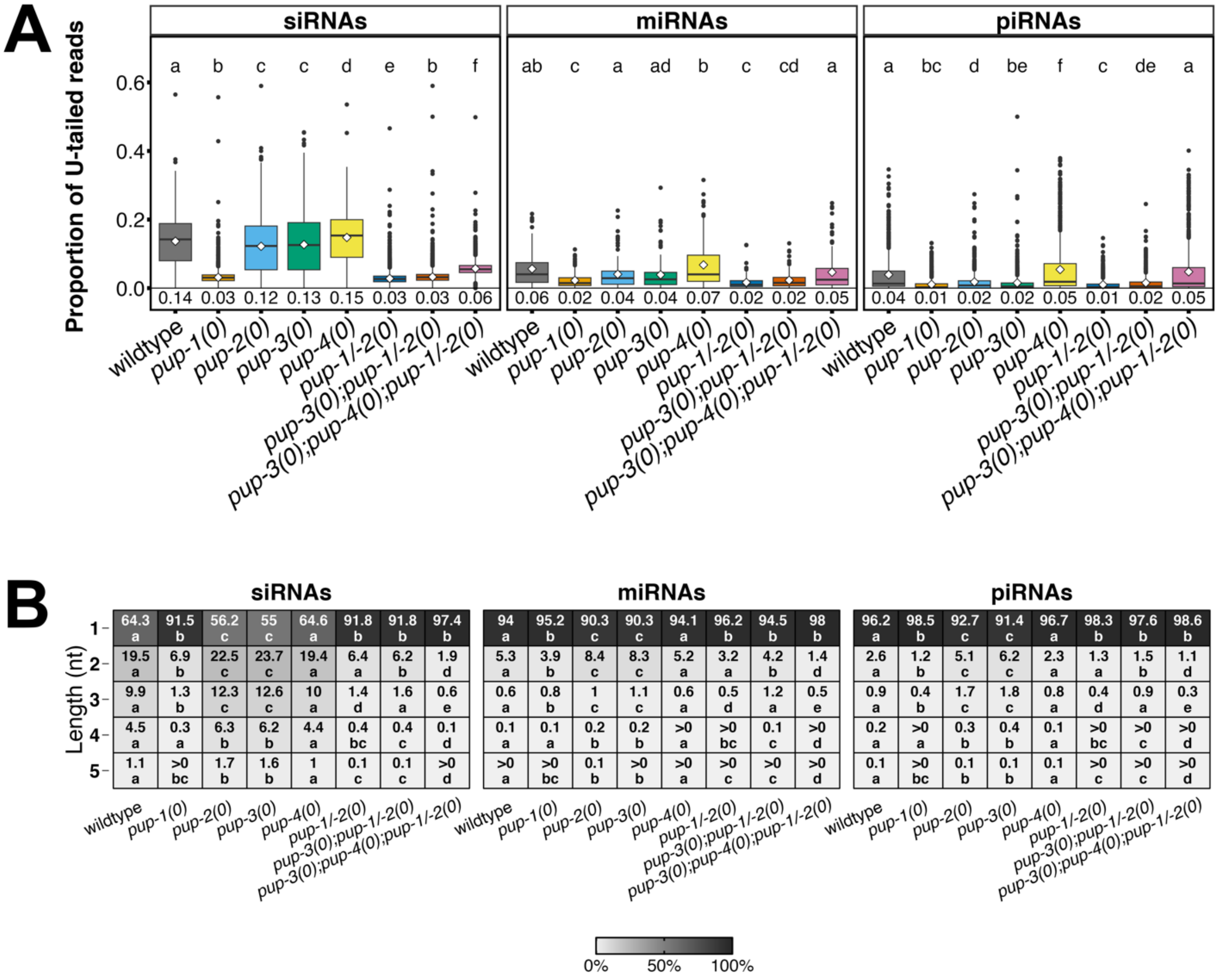

Fig. S4. Altered U-tailing of siRNA, miRNA, and piRNA in *pup* mutants. (A) Changes in the proportion of U-tailed reads for individual siRNAs, miRNAs, and piRNAs present at ≥10 CPM (see Fig. S2) in various *pup* mutants compared to wildtype. Genotypes are listed on the x-axis. Proportion of U-tailed reads for each siRNA, miRNA, and piRNA (number of U-tails/total number of reads) is indicated on the y-axis. Box, middle 50% of values; bar, median value; white diamond, average value, also indicated numerically below. Differences between genotypes were evaluated using a Kruskal-Wallis test followed by a Dunn's test with Bonferroni correction; genotypes that are not significantly different are indicated with a common letter. (B) Changes in the distribution of siRNA, miRNA, and piRNA U-tail lengths in *pup* mutants. Read counts for each sRNA type and tail length were compiled and divided

by the total number of U-tailed reads in the genotype/sRNA biotype dataset. Table lists the percentages of sRNA U-tailed reads by length (only 1-5 nt shown). Grayscale heatmap ranges from 100 (dark) to >0 (light). ">0" indicates <0.01% of reads. Within each length and sRNA biotype group, differences between genotypes were evaluated using a Kruskal-Wallis test followed by a Dunn's test with Bonferroni correction; genotypes that are not significantly different are indicated with a common letter.

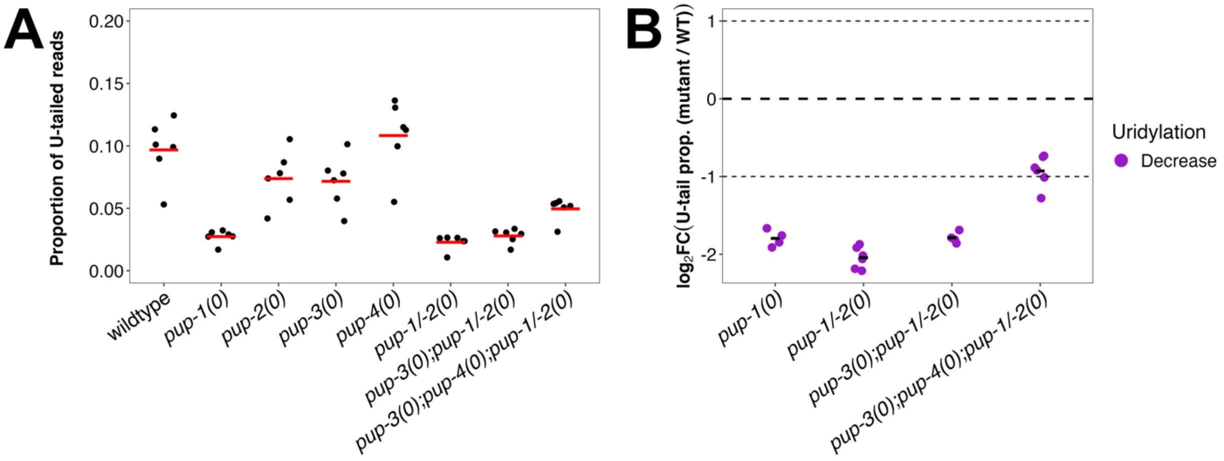

Fig. S5. Distribution of siRNAs mapping to transposable elements. (A) Average proportion of transposable element (TE) siRNAs that are U-tailed in wildtype and *pup* mutants, as indicated on the x-axis. Each dot represents a different TE type. Red line, average U-frequency. Y-axis, proportion of U-tailed reads for each TE type. (B) The $\log_2$FC in U-tail frequency in *pup* strains for TE siRNAs that are high confidence uridylation targets (detected only for PUP-1). X-axis, genotype. Y-axis, $\log_2$FC in the proportion of U-tailed TE siRNA reads in mutant compared to wildtype.

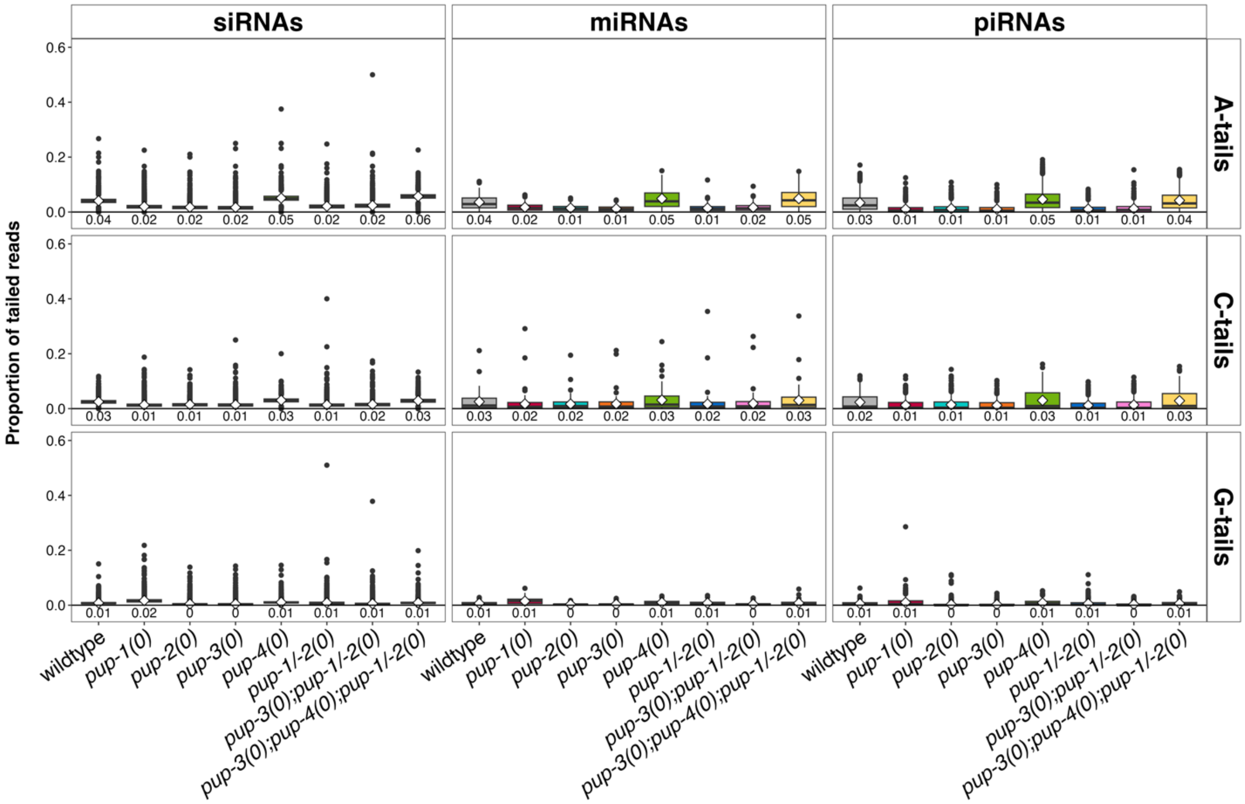

Fig. S6. The proportion of A-, C-, and G-tailed reads for individual siRNAs, miRNAs, and piRNAs in adult hermaphrodites is altered in some *pup* mutants compared to wildtype. All sRNAs present at ≥50 CPM are plotted. X-axis, genotypes. Y-axis, tail proportions were calculated as in Fig. S4A. Box, middle 50% of values; bar, median value; white diamond, average value, also listed numerically below.

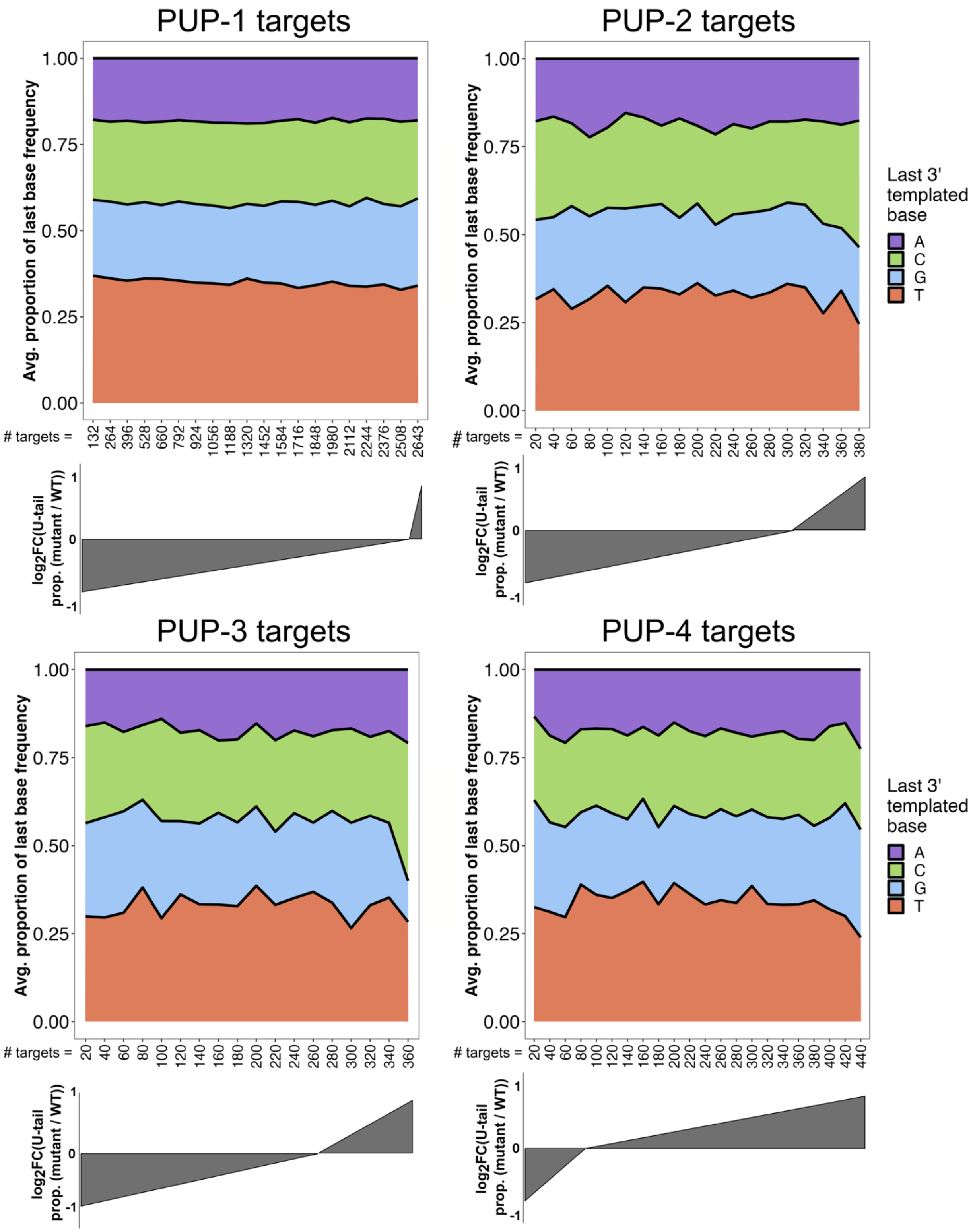
PUP-1 targets
PUP-2 targets
PUP-3 targets
PUP-4 targets
Avg. proportion of last base frequency
# targets =
log2FC(U-tail prop. (mutant / WT))
Last 3' templated base
A
C
G
T

Fig. S7. Distribution of 3' templated nucleotide among uridylated sRNAs targeted by PUP-1, PUP-2, PUP-3, and PUP-4. sRNAs are binned on the x-axis ordered by their log2 fold-change in proportion of U-tailed sRNAs in the indicated *pup* mutant compared to wildtype. Y-axis, average proportion of each 3' templated nucleotide for the corresponding bin of targets indicated on the x-axis. Each PUP-1 bin contains ~132 targets; each PUP-2, PUP-3, and PUP-4 bin contains ~20 targets. Binned targets are arranged from left to right in ascending order with respect to U-tailing. Values below 0 indicate reduced uridylation in *pup* mutant compared to wildtype; values above 0 indicate increased uridylation in *pup* mutant compared to wildtype. Colors indicate the 3' templated base.

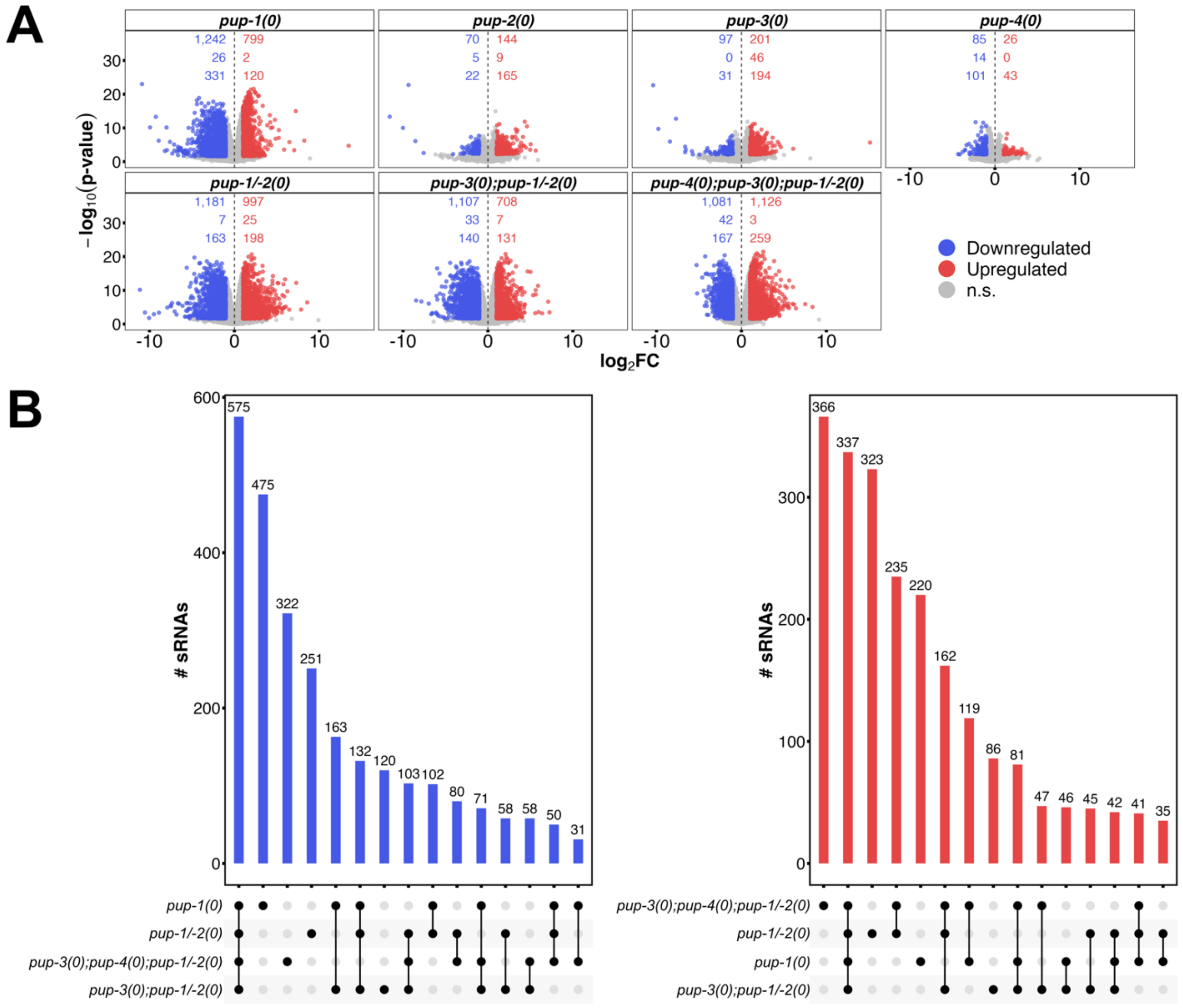

Fig. S8. Relationship among all sRNAs that are differentially abundant in one or more *pup* mutant strain compared to wildtype. (A) Volcano plots indicate all DA sRNAs in the indicated *pup* mutants regardless of U-tailing status. Blue points, downregulated sRNAs; red points, upregulated sRNAs; gray points, sRNAs without significantly altered abundance. Statistical significance was determined by a $|log_2FC| > 1$ and $FDR < 0.05$. (B) UpSet plots indicate downregulated (blue) and upregulated (red) sRNAs that are shared among and unique to different *pup* genotypes, as listed.

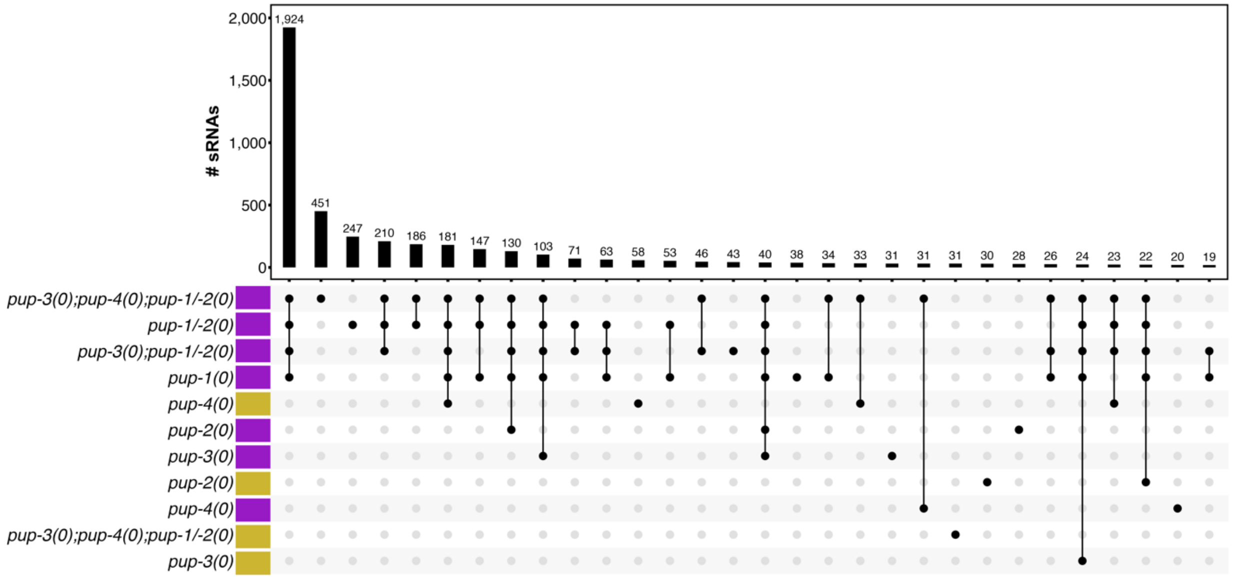

Fig. S9. Comparison of unique and shared PUP targets. This is an extension of the UpSet plot in Fig. 2B. Top 30 (out of 171) intersections are shown. Purple points, down-uridylated sRNAs; gold points, up-uridylated sRNAs.

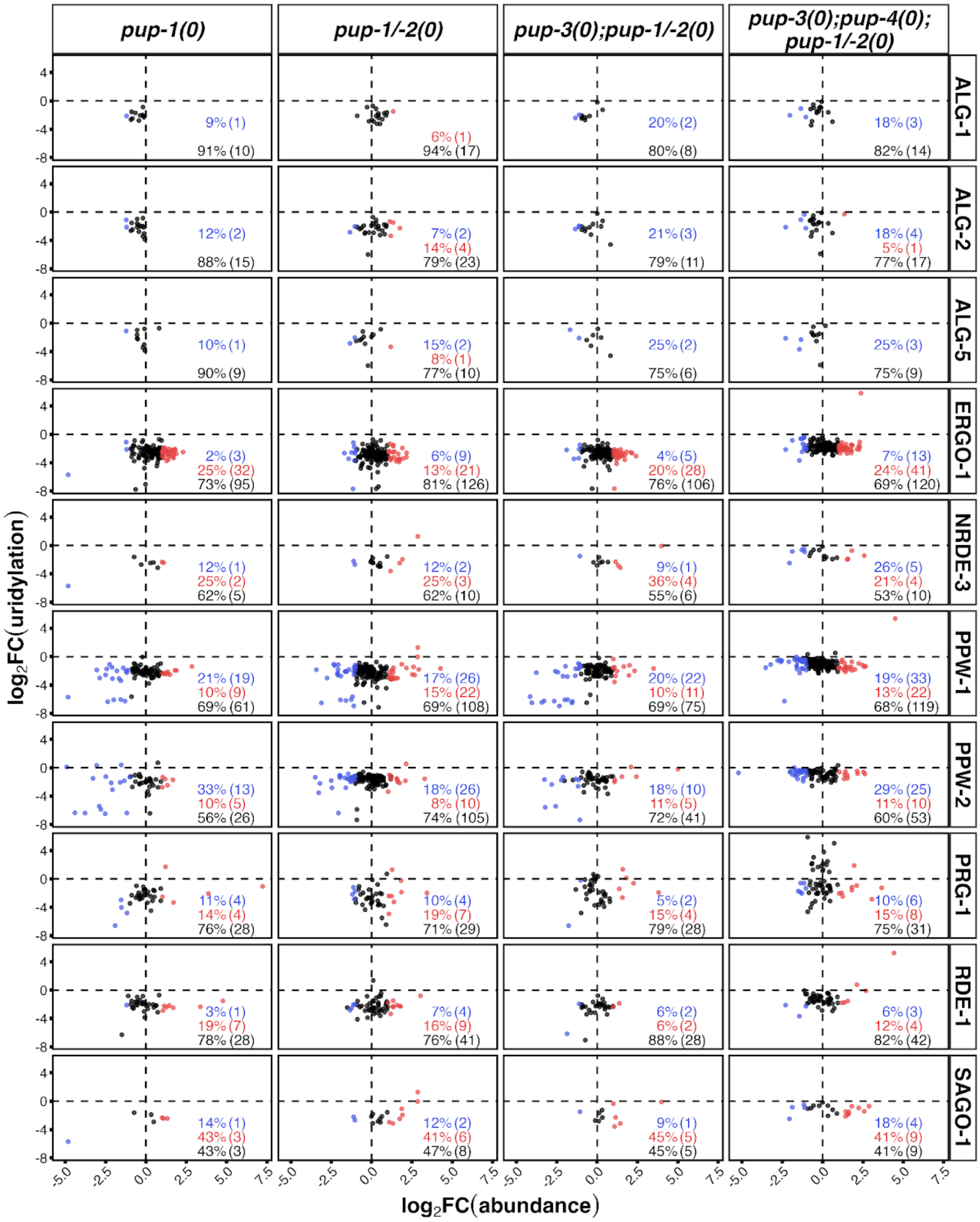

Fig. S10. Changes in sRNA abundance parsed by Argonaute association. These plots show Argonaute associations not included in Fig. 4B. See legend to Fig. 4.
