# Supplementary material for: Poly(U) polymerase activity in Caenorhabditis elegans regulates abundance and tailing of sRNA and mRNA
Source: Genetics. 2024 Jul 28;228(2):iyae120. doi: 10.1093/genetics/iyae120 (PMC11457939; doi:10.1093/genetics/iyae120)
Supplement: iyae120_Supplementary_Data [file iyae120_supplementary_data.zip › Supplemental_Tables_GENETICS-2024-307061.docx]

**Supplemental tables**

**Table S1. List of strains used in this study**

| **Strain Name** | **Genotype** |
| --- | --- |
| N2 | wildtype |
| EL602 | *pup-1/-2(om129)/qC1[dpy-19(e1259ts) glp-1(q339) nIs189[myo-2::gfp]]* |
| EL622 | *pup-1(tm1021)/qC1[dpy-19(e1259ts) glp-1(q339) nIs189[myo-2::gfp]]* |
| EL623 | *pup-2(tm4344)/qC1[dpy-19(e1259ts) glp-1(q339) nIs189[myo-2::gfp]]* |
| EL633 | *pup-3(tm5089);pup-1/-2(om129)/qC1[dpy-19(e1259ts) glp-1(q339) nIs189[myo-2::gfp]]* |
| EL694 | *pup-4(om140)* |
| EL713 | *pup-4(om141)* |
| EL716 | *omIs12[pup-4::3xflag]* |
| EL717 | *pup-3(tm5089);pup-4(om141);pup-1/-2(om129)/qC1[dpy-19(e1259ts) glp-1(q339) nIs189[myo-2::gfp]]* |
| EL725 | *omIs12[pup-4::3xflag];pup-1/-2(om129)/qC1[dpy-19(e1259ts) glp-1(q339) nIs189[myo-2::gfp]]* |
| EL733 | *pup-4(om141);omIs8[pup-1::3xmyc]omIs7[3xflag::pup-2]* |
| EL737 | *omIs10[3xflag::pup-3];pup-4(om141)* |
| EL738 | *omIs12[pup-4::3xflag];glp-1(q231ts)/hT2 [bli-4(e937) let-?(q272) qIs48]* |
| EL739 | *omIs12[pup-4::3xflag];glp-1(q231ts) pup-1/-2(om129)/hT2 [bli-4(e937) let-?(q272) qIs48]* |
| FX5089 | *pup-3(tm5089)* |

**Table S2. The *pup-4(0)* mutant has low penetrance germline defects**

A.

| Genotype | Gen | % fertile* (N) | % impaired germlines^  (# gonad arms) | % specific germline defects ** |
| --- | --- | --- | --- | --- |
| N2 wildtype | F2 | >99.5 (>500) | 0 (44) | --- |
|  | F3 | >99.5 (>500) | 0 (38) | --- |
|  | F4 | >99.5 (>500) | ND | ND |
| *pup-4(om141)* | F2 | >99 (>500) | 0 (36) | --- |
|  | F3 | >99.5 (766) | 13 (38) | 10 Emb(-), 3 Ooc(-) |
|  | F4 | >99.5 (749) | ND | ND |
| *pup-3(tm5089);pup-4(om141)* | F2 | >99.5 (>500) | 7 (30) | 7 Emo |
|  | F3 | >99.5 (771) | 30 (40) | 15 Emb(-), 10 Ooc(-), 5 Emo |
|  | F4 | >99.5 (462) | 48 (80) | 5 Gam(-), 1 Ooc(-), 41 Emo |

B.

| Genotype | Gen | # viable | % fertile | Most common germline defect(s) |
| --- | --- | --- | --- | --- |
| *pup-1(tm1021)* | F2 | 66 | 39 | ND |
|  | F3 | 20 | 4 | Gam(+/-), Gam(-) |
|  | F4 | 3 | 1 | Gam(-), GC(-) |
| *pup-1/-2(om129)* | F2 | 50 | 31 | GC(-) |
|  | F3 | 13 | 7 | GC(-) |
|  | F4 | 6 | 3 | GC(-) |
| *pup-4(om141);pup-1/-2(om129)* | F2 | 62 | 23 | Gam(-), GC(-) |
|  | F3 | 7 | 3 | GC(-) |
|  | F4 | 0 | 0 | GC(-) |
| *pup-3(tm5089);pup-4(om141);pup-1/-2(om129)* | F2 | 59 | 26 | GC(-) |
|  | F3 | 5 | 2 | Gam(+/-), Gam(-), GC(-) |
|  | F4 | 15 | 6 | Emb(-), Gam(-), GC(-) |

Animals were raised at 25°C. *, animals were scored at low magnification in a stereoscope for presence/absence of embryos in the uterus. ^DAPI-stained tissue was evaluated at high magnification; germline defects are described in the next column. **, defects are listed as a % of total germlines scored (including normal and impaired development). 3 trials were performed with wildtype and each genotype in parallel. Gen, generation. GC(-), no germ cells. Gam(-), few germ cells; no gametes. Gam(+/-), germ cells present; oocytes or sperm absent. Emb(-), germ cells, sperm, and oocytes present; embryos absent. Part A, impaired germline development is relatively rare; nearly all animals produced some viable offspring. Part B, fertility is severely impaired in all strains; the most common germline defect(s) observed in each mutant strain at each generation is (are) listed.

**Table S3. Most confidence sRNA targets of uridylation are siRNAs**

| **sRNA biotype** | **# High Confidence PUP targets** |
| --- | --- |
| siRNA | 4,520 |
| miRNA | 68 |
| piRNA | 188 |
